# Efficient Synthesis and Bioactivity of Novel Triazole Derivatives

**DOI:** 10.3390/molecules23040709

**Published:** 2018-03-21

**Authors:** Boyang Hu, Hanqing Zhao, Zili Chen, Chen Xu, Jianzhuang Zhao, Wenting Zhao

**Affiliations:** Key Laboratory of Urban Agriculture (North China), Ministry of Agriculture, College of Biological Science and Engineering, Beijing University of Agriculture, Beijing 102206, China; Huboyang_BUA@163.com (B.H.); chenzili1995@163.com (Z.C.); 13691458370@163.com (C.X.); zhaojianzhuang@263.net (J.Z.); wendyz0518@bua.edu.cn (W.Z.)

**Keywords:** triazole pesticide, carbohydrates, synthesis, fungicidal activity

## Abstract

Triazole pesticides are organic nitrogen-containing heterocyclic compounds, which contain 1,2,3-triazole ring. In order to develop potential glucosamine-6-phosphate synthase (GlmS) inhibitor fungicides, forty compounds of triazole derivatives were synthesized in an efficient way, thirty nine of them were new compounds. The structures of all the compounds were confirmed by high resolution mass spectrometer (HRMS), ^1^H-NMR and ^13^C-NMR. The fungicidal activities results based on means of mycelium growth rate method indicated that some of the compounds exhibited good fungicidal activities against *P. CapasiciLeonian*, *Sclerotinia sclerotiorum* (Lib.) de Bary, *Pyricularia oryzae* Cav. and *Fusarium oxysporum Schl. F.sp. vasinfectum* (Atk.) Snyd. & Hans. at the concentration of 50 µg/mL, especially the inhibitory rates of compounds **1-d** and **1-f** were over 80%. At the same time, the preliminary studies based on the Elson-Morgan method indicated that the compounds exhibited some inhibitory activity toward glucosamine-6-phosphate synthase (GlmS). These compounds will be further studied as potential antifungal lead compounds. The structure-activity relationships (SAR) were discussed in terms of the effects of the substituents on both the benzene and the sugar ring.

## 1. Introduction

Carbohydrates play an important role in the field of pesticide investigation, and many natural carbohydrate products used as pesticides have shown great vitality [1]. Carbohydrate compounds are easy to degrade, have good environmental compatibility [2], and are less resistant to resistance [3]. The new fungicides, which were developed based on carbohydrate compounds, have advantages of safety, high efficiency, low residue and not easy to generate insecticide resistance [4,5], not only ensuring the high yield of vegetables and other agricultural products [6], but also solve the problem of pesticide residues. 

Triazole pesticides are the most widely used variety of fungicides in agricultural production, which are mainly divided into fungicides, herbicides, pesticides and plant growth regulators [7]. At this stage, there are more than 30 varieties which have been produced and widely used, such as triazole alcohol [8], triazolone, propiconazole [9], hexaconazole [10] and fluorosilazole [11]. Triazole compounds have been extensively studied in the field of medicine and pesticides, and exhibit a variety of biological activities. In addition to a strong internal fungicidal activity, triazole compounds also have a regulatory role in the growth of plant [12], herbicidal [13], insecticidal [14] and antifungal [15,16]. Because of these multiple effect of the triazole structure, the research of these compounds have been in the ascendant so far. In order to obtain more efficient derivatives, the chemical structure modification of these compounds as well as the research as medicine and veterinary drugs have been frontier issues both at home and abroad for a long time.

**Figure 1:**
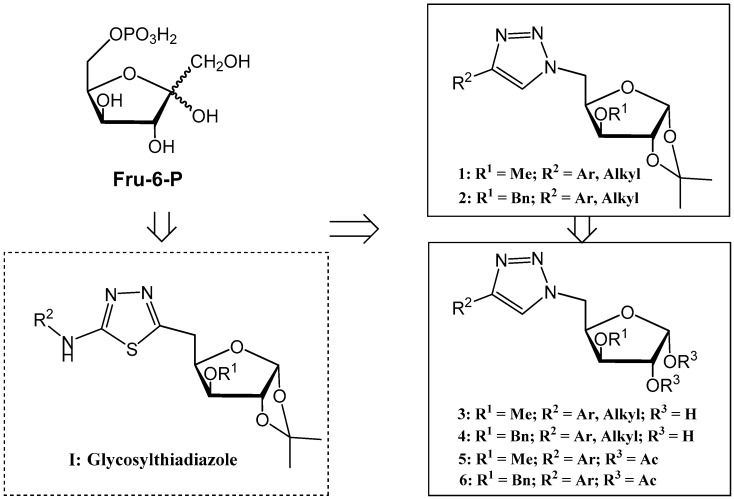
Structure of the thiadiazole (**I**) and triazole derivatives (**1**, **2**, **3**, **4**, **5**, **6**).

Biorational design based on specific target is one of the important means of international new pesticide creation. In the previous research in our laboratory, carbohydrate derivatives of five-membered ring thiadiazole derivatives (**I**, Figure 1) were synthesized and their fungicidal activities were studied as well [1]. The results of bioactivity determination showed that some compounds exhibited good fungicidal activities against *Sclerotinia sclerotiorum* (Lib.) de Bary and *Pyricularia oryzae* Cav. and consistent with their glucosamine-6-phosphate synthase (GlmS [17,18]; EC 2.6.1.16) inhibitory activities. 

**Scheme 1:**
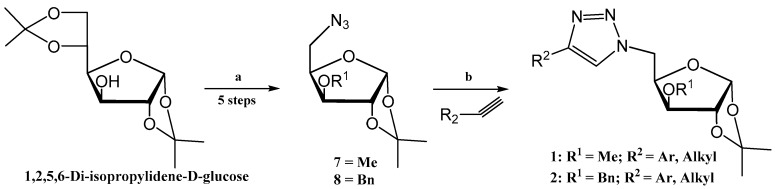

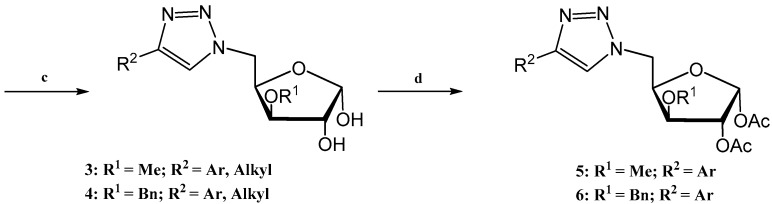
Synthesis of the target compounds **1**, **2**, **3**, **4**, **5** and **6**. *Reagents and conditions*: (**a**) MeI or BnBr, K_2_CO_3_, DMF, r.t.; then 70% AcOH, 70 °C, 2 h; NaIO_4_-SiO_2_, CH_2_Cl_2_, r.t.; NaBH_4_, EtOAc-H_2_O = 7:3, 0 °C to r.t., 15 min; Tf_2_O, pyridine, 0 °C to r.t., 15 min, then NaN_3_, DMF, 60 °C, 4 h; 62% for **7** (5 steps), 65% for **8** (5 steps); (**b**) CuSO_4_·5H_2_O, sodium ascorbate, CH_2_Cl_2_-CH_3_OH-H_2_O = 1:1:1, r.t., 1 h, 69–92% for **1**, 66–94% for **2**; (**c**) 90% CF_3_COOH, 0 °C to r.t., 2 h, 60–81% for **3**, 73–88% for **4**; (**d**) Ac_2_O, PDC, r.t., 2 h, 80–91% for **5**, 82–89% for **6**.

Inspired by these promising results, based on the structural characteristics of the substrate fructose-6-phosphate and the current research basis [1,19,20,21], we introduced the triazole group which possessed various important bioactivities instead of thiadiazole in this article, six series of novel furan glucosyl triazole compounds were designed and synthesized for the first time, their fungicidal activities against *P. CapasiciLeonian*, *Sclerotinia sclerotiorum* (Lib.) de Bary, *Botrytis cinerea Pers*, *Pyricularia oryzae* Cav., *Fusarium oxysporum Schl. F.sp. vasinfectum* (Atk.) Snyd. & Hans. and enzyme inhibitory activities against *Candida albicans* GlmS were evaluated. We would like to report their synthesis (Scheme 1) and bioactivities in much greater details, and also their structure-activity relationship studies. We report herein the preliminary results of the study.

## 2. Results and Discussion

### 2.1. Synthesis of the Title Compounds

As shown in Scheme 1, we envisioned that the target compounds triazole derivatives **1**, **2**, **3**, **4**, **5** and **6** could be synthesized from the intermediates **7** [22] and **8** [23] which could be prepared using 1,2,5,6-*di*-isopropylidene-d-glucose as the starting material for five steps according to the known methods [22,23]. Then cyclization of **7** and **8** with alkyne provided the desired triazole derivatives **1** and **2** under the conditions of copper sulfate and sodium ascorbate in high yields, respectively. Then the target compounds **3** or **4** were prepared by treating compounds **1** or **2** with 90% trifluoroacetic acid. Compounds **5** or **6** were obtained by acetylation of compound **3** or **4**. 

All the derivatives were synthesized according to the procedures described in Scheme 1 in good yields of 60–98%. The structures of all the synthesized compounds were confirmed from its ^1^H-NMR, ^13^C-NMR spectra and HRMS. The ^1^H-NMR experiments of compounds **1**/**2**/**5**/**6** were conducted in CDCl_3_ as the solvent. Nevertheless, because of the poor solubility of compounds **3**/**4**, so we had to switch the solvent to methanol-d_4_ or DMSO-d_6_. The physical data of the target compounds were given in Table 1.

### 2.2. Fungicidal Activity of Compounds ***1***, ***2***, ***3***, ***4***, ***5***, ***6*** against Five Fungus Species

Fungicidal activities of the target compounds **1**, **2**, **3**, **4**, **5**, **6** against five fungal species were evaluated as previously reported [25] and compared with the commercial fungicide chlorothalonil. The inhibition rates were given in Table 2. The determination results showed that, most of the tested compounds displayed a certain degree of fungicidal activity against the five species at the concentration of 50 µg/mL. 

In general, the following structure-activity relationships (SAR) in compounds **1**, **2**, **3**, **4**, **5** and **6** were observed: (1) As a whole, series **1** and **2** exhibited good fungicidal activities against *P. CapasiciLeonian*, *Sclerotinia sclerotiorum* (Lib.) de Bary, *Pyricularia oryzae* Cav. and *Fusarium oxysporum Schl. F.sp. vasinfectum* (Atk.) Snyd. & Hans. than the other series. (2) For the series **1** and **2**, the fungicidal activities were increased by improving the electron-withdrawing ability of substituents on the benzene ring such as compounds **1-d** (R^2^ = 4-F-C_6_H_4_-), **1-e** (R^2^ = 4-NO_2_-C_6_H_4_-), **1-f** (R^2^ = 4-Cl-C_6_H_4_-), **2-d** (R^2^ = 4-F-C_6_H_4_-), **2-e** (R^2^ = 4-NO_2_-C_6_H_4_-) and **2-f** (R^2^ = 4-Cl-C_6_H_4_-); When the substituent group (R^2^) were substituted phenyl, the fungicidal activities of series compounds were superior to that with substituent alkyl. (3) Compared series **1** and **2**, on an overall level the former (R^1^ = Me) displayed a better fungicidal activities than the latter (R^1^ = Bn). (4) Series **3** and **4** were obtained by deisopropylidenation of the compounds **1** and **2**, they had the better water-solubility, but the fungicidal activities of compounds **3** and **4** against five species were decreased obviously. (5) In order to improve the fat solubility, the compounds **5** and **6** were synthesized. The fungicidal activities of compounds **5** and **6** were better than compounds **3** and **4**, but lower than compounds **1** and **2**.

### 2.3. Bioassay of Enzyme Inhibitory Activities 

Inhibitory activities of all the synthesized compounds towards *Candida albicans* GlcN-6-P synthase were evaluated using the optimized Elson-Morgan method as previously reported [25]. The absorption value of the solution was measured at 585 nm, and then the concentration was counted by the specification curve which was determined thanks to the relation between the absorption value and the concentration of glucosamine-6-phosphate. The inhibition rates were given in Table 3 at 0.35 mm. 

As was shown, most of the tested compounds exhibited some enzyme inhibitory activities against glucosamine-6-phosphate synthase at 0.35 mm. On the whole, Although Series **1** and **2** displayed a better fungicidal activities against five species, they exhibited poor enzyme inhibitory activities. Series **3** and **4** without the OH-protection at both 1- and 2-position exhibited better enzyme inhibitory activities than the other series. By and large, the enzyme inhibitory activities of Series **5** and **6** with the OH-acetylation at 1 and 2-position were better than Series **1** and **2** but lower than Series **3** and **4**. Compounds **3-a**, **3-d**, **3-e** and **3-f** were more active against glucosamine-6-phosphate synthase than the other compounds.

## 3. Experimental Section

### 3.1. General Methods

All starting materials and reagents purchased from Sigma-Aldrich (Beijing, China) and Sinopharm Chemical Reagent Beijing Co., Ltd. (Beijing, China). Solvents were purified in the usual way. All reactions were carried out under a nitrogen atmosphere if necessary. All reactions were monitored by thin-layer chromatography (TLC) (the Silica gel thin plate purchased from Yantai Dexin Biological Technology Co., Ltd., Yantai, China) analysis and TLC was performed on silica gel HF with detection by charring with 30% (*v*/*v*) H_2_SO_4_ in CH_3_OH or by UV detection (254 nm). Column chromatography was conducted by elution of a column (8 × 100, 16 × 240, 18 × 300, 35 × 400 mm) of silica gel (200–300 mesh) with EtOAc–PE (b. p. 60–90 °C) as the eluent. Optical rotations were recorded using a Perkin-Elmer 241 polarimeter (Perkin-Elmer, Waltham, MA, USA). ^1^H-NMR (400 MHz) and ^13^C-NMR (100 MHz) spectra was recorded in CDCl_3_, Meth-d_4_ or DMSO-*d*_6_ with a Bruker DPX400 spectrometer (Brook (Beijing) science and Technology Co., Ltd., Beijing, China), using Tetramethyl silane (TMS) as internal standard; Mass spectra were obtained with Agilent 1100 series LC/MSD mass spectrometer (Agilent Technologies Inc., Beijing, China). High-resolution mass spectra (HRMS) were performed by the Peking University. Melting points were measured on a Yanagimoto melting-point apparatus (Yanagimoto MFG CO, Kyoto, Japan) and are uncorrected. Solutions were concentrated at a temperature <60 °C under diminished pressure.

### 3.2. Chemical Synthesis

*General procedure for the synthesis of title compounds* **1**/**2**. To a soln of compound **7** [22] or **8** [23] (1.5 g) in 1:1:1 CH_2_Cl_2_–CH_3_OH–H_2_O (30 mL) was added alkyne derivaticves (0.35 mL), CuSO_4_·5H_2_O (0.45 g) and sodium ascorbate (0.315 g). The mixture was stirred at 40 °C for 10 h, and TLC (6:1 petroleum ether–EtOAc) indicated that the reaction was complete. The aq. soln. was extracted with CH_2_Cl_2_ (3 × 50 mL), washed with saturated aq. sodium bicarbonate, dried (Na_2_SO_4_) and concentrated. Purification by silica gel chromatography with 7:1 petroleum ether–EtOAc as the eluent afforded **1** or **2**.

*General procedure for the synthesis of title compounds* **3**/**4**. Compound **1** or **2** (0.8 g) was dissolved in 90% aq trifluoroacetic acid (20 mL) and then stirred at 40 °C for 4 h, and TLC (1:1 petroleum ether–EtOAc) indicated that the reaction was complete. The trifluoroacetic acid was evaporated under reduced pressure, then the residue was diluted with CH_2_Cl_2_ (50 mL), washed with saturated aq. sodium bicarbonate, and dried over Na_2_SO_4_. The soln was concentrated, and the residue was subjected to column chromatography (2:1 petroleum ether–EtOAc) to give the desired product **3**/**4**. 

*General procedure for the synthesis of title compounds* **5**/**6**. To a stirred of compound **3** or **4** (0.4 g) in pyridine (5 mL) was added acetic anhydride (3 mL). The mixture was stirred for a further 3 h, at the end of which time TLC (eluent: 4:1 petroleum ether–EtOAc) indicated that the reaction was complete. The solvents were evaporated under reduced pressure to give a crude product, which was purified on silica gel column chromatography with 5:1 petroleum ether–EtOAc as the eluent to give the compounds **5/6**.

*Furan glucosyl-1,2,3-triazole* (**1-a**). Yield: 79.4%. White solid, m.p. 159.9–160.4 °C. ^1^H-NMR (CDCl_3_): δ 7.89–7.31 (m, 6H, ArH, CCHN), 5.95 (s, 1H, H-1), 4.77–3.76 (m, 5H, H-2, H-3, H-4, H-5, H-6), 3.44 (s, 3H, CH_3_O), 1.43, 1.30 (2s, 6H, Me_2_C); ^13^C-NMR (CDCl_3_): δ 147.44, 130.53, 128.56, 127.81, 125.48, 120.69, 111.74, 105.01, 83.76, 81.16, 78.65, 57.53, 48.82, 26.52, 25.98. ESI-MS *m/z* calcd. for C_17_H_22_O_4_N_3_ [M + H]^+^ 332.1. Found: 332.1. HRMS for C_17_H_22_O_4_N_3_ [M + H]^+^ 332.1610. Found: 332.1602.

*Furan glucosyl-1,2,3-triazole* (**1-b**). Yield: 79.6%. Yellow solid, m.p. 108.0–112.7 °C. ^1^H-NMR (CDCl_3_): δ 7.88–7.12 (m, 5H, ArH, CCHN), 5.95 (s, 1H, H-1), 4.76–3.75 (m, 5H, H-2, H-3, H-4, H-5, H-6), 3.43 (s, 3H, CH_3_O), 2.38 (s, 3H, Ar-Me), 1.43, 1.30 (2s, 6H, Me_2_C); ^13^C-NMR (CDCl_3_): δ 138.34, 130.51, 128.77, 128.61, 126.33, 122.78, 120.71, 111.98, 105.17, 83.97, 81.32, 78.82, 57.73, 48.98, 26.68, 26.14, 21.32. ESI-MS *m/z* calcd. for C_18_H_24_O_4_N_3_ [M + H]^+^ 347.1. Found: 347.1. HRMS for C_18_H_24_O_4_N_3_ [M + H]^+^: 347.1719. Found: 347.1718.

*Furan glucosyl-1,2,3-triazole* (**1-c**). Yield: 69.3%. White solid, m.p. 156.6–157.5 °C. ^1^H-NMR (CDCl_3_): δ 7.82–6.93 (m, 5H, ArH, CCHN), 5.96 (d, 1H, *J* = 4.0 Hz, H-1), 4.77–4.50 (m, 4H), 3.83–3.76 (m, 4H), 3.44 (s, 3H, CH_3_O) 1.43, 1.32 (2s, 6H, Me_2_C); ^13^C-NMR (CDCl_3_): δ 159.47, 147.49, 126.90, 123.35, 119.92, 114.12, 111.89, 105.12, 83.91, 81.28, 78.79, 57.67, 55.16, 48.88, 26.63, 26.09. ESI-MS *m/z* calcd. for C_18_H_24_O_5_N_3_ [M + H]^+^ 362.1. Found: 362.1. HRMS for C_18_H_24_O_5_N_3_ [M + H]^+^ 362.1716. Found: 362.1710.

*Furan glucosyl-1,2,3-triazole* (**1-d**). Yield: 83.0%. White solid, m.p. 121.4–122.3 °C. ^1^H-NMR (CDCl_3_): δ 7.88–7.08 (m, 5H, ArH, CCHN), 5.97 (s, 1H, H-1), 4.78–3.79 (m, 5H, H-2, H-3, H-4, H-5, H-6), 3.45 (s, 3H, CH_3_O), 1.43, 1.31 (2s, 6H, Me_2_C); ^13^C-NMR (CDCl_3_): δ 163.77, 161.31, 146.83, 127.45, 127.37, 127.07, 126.97, 126.94, 120.57, 115.77, 115.55, 112.00, 105.19, 84.03, 81.33, 78.82, 77.48, 57.73, 49.12, 26.27, 26.12. ESI-MS *m/z* calcd. for C_17_H_21_O_4_N_3_F [M + H]^+^ 350.1. Found: 350.1. HRMS for C_17_H_21_O_4_N_3_F [M + H]^+^ 350.1516. Found: 350.1515.

*Furan glucosyl-1,2,3-triazole* (**1-e**). Yield: 77.1%. White solid, m.p. 126.5–126.8 °C. ^1^H-NMR (CDCl_3_): δ 8.28–8.01 (m, 5H, ArH, CCHN), 5.99 (s, 1H, H-1), 4.84–3.85 (m, 5H, H-2, H-3, H-4, H-5, H-6), 3.49 (s, 3H, CH_3_O), 1.44, 1.33 (2s, 6H, Me_2_C); ^13^C-NMR (CDCl_3_) δ: 147.25, 145.61, 137.04, 126.15, 124.22, 122.49, 112.13, 105.26, 84.16, 81.38, 78.77, 57.85, 49.59, 26.71, 26.15. ESI-MS *m/z* calcd. for C_17_H_21_O_6_N_4_ [M + H]^+^ 377.1. Found: 377.1. HRMS for C_17_H_21_O_6_N_4_ [M + H]^+^ 377.1461. Found: 377.1461.

*Furan glucosyl-1,2,3-triazole* (**1-f**). Yield: 79.3%. White solid, m.p. 135.9–136.7 °C. ^1^H-NMR (CDCl_3_): δ 7.90–7.37 (m, 5H, ArH, CCHN), 5.97 (s, 1H, H-1), 4.78–3.79 (m, 5H, H-2, H-3, H-4, H-5, H-6) 3.46 (s, 3H, CH_3_O) 1.43, 1.31 (2s, 6H, Me_2_C); ^13^C-NMR (CDCl_3_): δ 146.74, 133.75, 129.27, 128.98, 127.00, 120.93, 112.10, 105.25, 84.11, 81.38, 78.85, 57.83, 49.27, 26.74, 26.20. ESI-MS *m/z* calcd. for C_17_H_21_O_4_N_3_Cl [M + H]^+^ 366.1. Found: 366.1. HRMS for C_17_H_21_O_4_N_3_Cl [M + H]^+^ 366.1221. Found: 366.1219.

*Furan glucosyl-1,2,3-triazole* (**1-g**). Yield: 92.3%. Yellow solid, m.p. 90.2–90.9 °C. ^1^H-NMR (CDCl_3_): δ 7.61 (d, *J* = 4.0 Hz, 1H, CCHN), 5.94 (s, 1H, H-1), 5.09–5.04 (m, 1H, CH_3_CHOH), 4.68–4.47 (m, 4H), 3.75 (s, 1H), 3.44 (s, 3H, CH_3_O), 1.58 (s, 3H, OH-C-CH_3_)1.43,1.30 (2s, 6H, Me_2_C); ^13^C-NMR (CDCl_3_): δ 162.75, 152.52, 152.46, 121.43, 112.08, 105.21, 84.00, 81.36, 78.80, 62.83, 62.77, 57.79, 48.99, 29.67, 26.73, 26.19, 23.09, 23.00, 20.96. ESI-MS *m/z* calcd. for C_13_H_23_O_5_N_3_ [M + H]^+^ 300.1. Found: 300.1. HRMS for C_13_H_23_O_5_N_3_ [M + H]^+^ 300.1559. Found: 300.1555.

*Furan glucosyl-1,2,3-triazole* (**2-a**) [24]. Yield: 66.0%. White solid, m.p. 133.2–134.1 °C. ^1^H-NMR (CDCl_3_): δ 7.79–7.25(m, 11H, ArH, CCHN), 5.96 (s, 1H, H-1), 4.69–4.42 (m, 6H, H-2, H-3, H-4, H-5, *C*H_2_Ar), 3.97 (s, 1H, H-6), 1.40, 1.27 (2s, 6H, Me_2_C); ^13^C-NMR (CDCl_3_): δ 147.53, 136.84, 128.60, 128.52, 128.13, 127.86, 127.79, 125.57, 120.73, 111.92, 111.90, 81.86, 81.61, 78.76, 77.48, 76.84, 76.81, 71.87, 49.09, 26.62, 26.08. ESI-MS *m/z* calcd. for C_23_H_26_O_4_N_3_ [M + H]^+^ 408.1 Found: 408.1. HRMS for C_23_H_26_O_4_N_3_ [M + H]^+^ 408.1923. Found: 408.1922.

*Furan glucosyl-1,2,3-triazole* (**2-b**). Yield: 79.4%. White solid, m.p. 108.8–110.2 °C. ^1^H-NMR (CDCl_3_): δ 7.77–7.09 (m, 11H, ArH, CCHN), 5.98 (s, 1H, H-1), 4.72–4.44 (m, 6H, H-2, H-3, H-4, H-5, CH_2_Ar), 3.98 (s, 1H, H-6), 2.36 (s, 3H, CH_3_Ar), 1.41,1.29 (2s, 6H, Me_2_C); ^13^C-NMR (CDCl_3_): δ 147.72, 138.24, 136.92, 130.51, 128.69, 128.57, 128.17, 127.83, 126.30, 122.77, 120.68, 111.98, 105.18, 81.96, 81.74, 78.84, 71.96, 49.12, 26.67, 26.14, 21.27. ESI-MS *m/z* calcd. for C_24_H_28_O_4_N_3_ [M + H]^+^ 422.2. Found: 422.2. HRMS for C_24_H_28_O_4_N_3_ [M + H]^+^ 422.2080. Found: 422.2078.

*Furan glucosyl-1,2,3-triazole* (**2-c**). Yield: 73.6%. White solid, m.p. 134.6–135.1 °C. ^1^H-NMR (CDCl_3_): δ 7.73–6.92 (m, 11H, ArH, CCHN), 6.00 (s, 1H, H-1), 4.75–4.48 (m, 6H, H-2, H-3, H-4, H-5, CH_2_Ar), 4.01–3.81 (m, 4H, CH_3_O, H-6), 1.42, 1.30 (2s, 6H, Me_2_C); ^13^C-NMR (CDCl_3_): δ 159.58, 147.64, 136.96, 128.71, 128.32, 127.96, 127.04, 123.42, 120.03, 114.21, 112.13, 105.28, 82.03, 81.78, 78.97, 72.07, 55.29, 49.23, 26.77, 26.24. ESI-MS *m/z* calcd. for C_24_H_28_O_5_N_3_ [M + H]^+^ 438.2. Found: 438.2. HRMS for C_24_H_28_O_5_N_3_ [M + H]^+^ 438.2029. Found: 438.2044.

*Furan glucosyl-1,2,3-triazole* (**2-d**). Yield: 90.4%. White solid, m.p. 164.3–164.7 °C. ^1^H-NMR (CDCl_3_): δ 7.77–7.06 (m, 10H, ArH, CCHN), 6.00 (s, 1H, H-1), 4.75–4.48 (m, 6H, H-2, H-3, H-4, H-5, CH_2_Ar), 4.02 (s, 1H, H-6), 1.42, 1.31 (2s, 6H, Me_2_C); ^13^C-NMR (CDCl_3_): δ 146.92, 136.95, 128.73, 128.36, 127.98, 127.53, 127.45, 126.99, 120.63, 115.82, 115.61, 112.18, 105.31, 82.07, 81.83, 78.94, 72.09, 49.40, 26.77, 26.24. ESI-MS *m/z* calcd. for C_23_H_25_O_4_N_3_F [M + H]^+^ 426.1. Found: 426.1. HRMS for C_23_H_25_O_4_N_3_F [M + H]^+^ 426.1829. Found: 426.1830.

*Furan glucosyl-1,2,3-triazole* (**2-e**). Yield: 90.2%. White solid, m.p. 182.6–184.6 °C. ^1^H-NMR (CDCl_3_): δ 8.28–7.33 (m, 11H, ArH, CCHN), 6.03 (s, 1H, H-1), 4.80–4.50 (m, 6H, H-2, H-3, H-4, H-5, CH_2_Ar), 4.07 (s, 1H, H-6), 1.43, 1.33 (2s, 6H, Me_2_C); ^13^C-NMR (CDCl_3_): δ 147.33, 145.67, 137.02, 136.90, 128.81, 128.46, 128.03, 126.21, 124.27, 122.51, 112.29, 105.37, 82.08, 81.83, 78.84, 72.12, 49.80, 26.79, 26.25. ESI-MS *m/z* calcd. for C_23_H_24_O_6_N_4_ [M + H] 453.1. Found: 453.1. HRMS for C_23_H_24_O_6_N_4_ [M + H]^+^ 453.1774. Found: 453.1773.

*Furan glucosyl-1,2,3-triazole* (**2-f**). Yield: 93.7%. White solid, m.p. 136.2–136.5 °C. ^1^H-NMR (CDCl_3_): δ 7.79–7.33 (m, 11H, ArH, CCHN), 6.01 (s, 1H, H-1), 4.77–4.48 (m, 6H, H-2, H-3, H-4, H-5, CH_2_Ar), 4.03 (s, 1H, H-6), 1.43, 1.31 (2s, 6H, Me_2_C); ^13^C-NMR (CDCl_3_): δ 146.80, 136.94, 133.82, 129.28, 129.02, 128.80, 128.44, 128.03, 127.06, 120.98, 112.24, 105.35, 82.08, 81.82, 78.95, 77.16, 76.84, 72.13, 49.51, 26.82, 26.29. ESI-MS *m/z* calcd. for C_23_H_25_O_4_N_3_Cl [M + H]^+^ 442.1. Found: 442.1. HRMS for C_23_H_25_O_4_N_3_Cl [M + H]^+^ 442.1534. Found: 442.1533.

*Furan glucosyl-1,2,3-triazole* (**2-g**). Yield: 87.6%. White solid, m.p. 90.6–90.9 °C. ^1^H-NMR (CDCl_3_): δ 7.46–7.23 (m, 6H, ArH, CCHN), 5.86 (s, 1H, H-1), 4.93–4.92 (s, 1H), 4.63–4.39 (m, 6H, H-2, H-3, H-4, H-5, CH_2_Ar), 3.89 (s, 1H, H-6), 1.45 (d, *J* = 4.0 Hz, 3H, OH-C-CH_3_) 1.31,1.20 (2s, 6H, Me_2_C); ^13^C-NMR (CDCl_3_): δ 136.84, 128.51, 128.11, 127.79, 121.33, 111.90, 105.06, 81.87, 81.57, 78.73, 71.87, 62.62, 62.57, 48.99, 26.57, 26.05, 23.04, 22.96. ESI-MS *m/z* calcd. for C_19_H_27_O_5_N_3_ [M + H]^+^ 376.1. Found: 376.1. HRMS for C_19_H_27_O_5_N_3_ [M + H]^+^ 376.1872. Found: 376.1875.

*Furan glucosyl-1,2,3-triazole* (**3-a**). Yield: 78.5%. White solid, m.p. 101.1–102.1 °C. ^1^H-NMR (Meth-d_4_): δ 8.18–7.21 (m, 5H, ArH, CCHN), 5.26–5.08 (m, 1H, H-1, α and β), 4.82–3.71 (m, 5H, H-2, H-3, H-4, H-5, H-6, α and β), 3.36 (m, 3H, OMe); ^13^C-NMR (Meth-d_4_): δ 148.60, 148.54, 131.65, 131.63, 129.92, 129.27, 126.63, 123.26, 123.09, 104.80, 97.85, 87.13, 86.70, 80.54, 79.71, 77.50, 76.00, 58.42, 52.55, 51.73. ESI-MS *m/z* calcd. for C_14_H_18_O_4_N_3_ [M + H]^+^ 292.1. Found: 292.1. HRMS for C_14_H_18_O_4_N_3_ [M + H]^+^ 292.1297. Found: 292.1293.

*Furan glucosyl-1,2,3-triazole* (**3-b**). Yield: 77.4%. Oily. ^1^H-NMR (Meth-d_4_): δ 8.19–6.98 (m, 5H, ArH, CCHN), 5.27–5.10 (m, 1H, H-1, α and β), 4.60–3.69 (m, 5H, H-2, H-3, H-4, H-5, H-6, α and β), 3.32 (s, 3H, CH_3_O), 2.20 (s, 3H, Ar-CH_3_); ^13^C-NMR (Meth-d_4_): δ 148.52, 148.45, 139.68, 131.23, 130.00, 129.80, 127.16, 123.74, 123.24, 123.04, 104.73, 97.80, 87.02, 86.61, 80.44, 79.61, 77.43, 75.84, 58.41, 58.31, 52.55, 51.71, 21.44. ESI-MS *m/z* calcd. for C_15_H_20_O_4_N_3_ [M + H]^+^ 306.1. Found: 306.1. HRMS for C_15_H_20_O_4_N_3_ [M + H]^+^ 306.1454. Found: 306.1456.

*Furan glucosyl-1,2,3-triazole* (**3-c**). Yield: 79.4%. White solid, m.p. 130.3–130.8 °C. ^1^H-NMR (Meth-d_4_): δ 8.06–6.83 (m, 5H, ArH, CCHN), 5.26–5.07 (m, 1H, H-1, α and β), 4.79–3.98 (m, 5H, H-2, H-3, H-4, H-5, H-6, α and β), 3.78–3.76 (m, 3H, CH_3_O-Ar), 3.20 (m, 3H, OMe); ^13^C-NMR (Meth-d4): δ 160.20, 148.54, 148.48, 127.97, 124.22, 124.19, 122.39, 122.22, 115.33, 104.79, 97.84, 87.11, 86.69, 80.55, 79.69, 77.52, 75.98, 58.41, 58.34, 55.77, 52.47, 51.66. ESI-MS *m/z* calcd. for C_15_H_30_O_5_N_3_ [M + H]^+^ 322.1. Found: 322.1. HRMS for C_15_H_30_O_5_N_3_ [M + H]^+^ 322.1403. Found: 322.1399.

*Furan glucosyl-1,2,3-triazole* (**3-d**). Yield: 64.9%. White solid, m.p. 136.5–137.1 °C. ^1^H-NMR (Meth-d_4_): δ 8.15–7.00 (m, 5H, ArH, CCHN), 5.27–5.08 (m, 1H, H-1, α and β), 4.76–4.50 (m, 5H, H-2, H-3, H-4, H-5, H-6, α and β), 3.45–3.20 (m, 3H, OMe); ^13^C-NMR (Meth-d4): δ 163.91, 161.47, 146.40, 146.33, 127.29, 127.21, 126.85, 126.81, 126.78, 121.78, 121.62, 115.48, 115.27, 103.49, 96.55, 85.80, 85.39, 79.20, 78.39, 76.20, 74.66, 57.11, 57.03, 51.21, 50.40. ESI-MS *m/z* calcd. for C_14_H_17_O_4_N_3_F [M + H]^+^ 310.1. Found: 310.1. HRMS for C_14_H_17_O_4_N_3_F [M + H]^+^ 310.1203. Found: 310.1204.

*Furan glucosyl-1,2,3-triazole* (**3-e**). Yield: 74.3%. White solid, m.p. 149.2–150.1 °C. ^1^H-NMR (DMSO): δ 8.81–8.14 (m, 5H, ArH, CCHN), 6.19–6.11 (m, 1H, H-1, α and β), 5.00–4.56 (m, 4H), 4.06–3.99 (m, 1H), 3.44–3.37 (m, 3H, OMe); ^13^C-NMR (DMSO): δ 146.69, 146.59, 144.42, 144.20, 137.05, 137.05, 125.98, 125.86, 124.35, 103.29, 96.29, 85.92, 85.07, 78.89, 77.73, 75.39, 74.31, 57.50, 57.44, 51.06, 50.39. ESI-MS *m/z* calcd. for C_14_H_17_O_6_N_4_ [M + H]^+^ 337.1. Found: 337.1. HRMS for C_14_H_17_O_6_N_4_ [M + H]^+^ 337.1148. Found: 337.1146.

*Furan glucosyl-1,2,3-triazole* (**3-f**). Yield: 80.7%. White solid, m.p. 109.4–110.2 °C. ^1^H-NMR (Meth-d_4_): δ 8.19–7.25 (m, 5H, ArH, CCHN), 5.29–5.10 (m, 1H, H-1, α and β), 5.07–3.72 (m, 5H, H-2, H-3, H-4, H-5, H-6, α and β), 3.46–3.21 (m, 3H, OMe); ^13^C-NMR (Meth-d_4_): δ 133.62, 129.00, 128.98, 128.74, 126.75, 122.20, 122.01, 103.46, 96.56, 85.76, 85.37, 79.21, 78.32, 76.22, 74.59, 57.18, 57.08, 51.32, 50.48. ESI-MS *m/z* calcd. for C_14_H_17_O_4_N_3_Cl [M + H]^+^ 326.0. Found: 326.0. HRMS for C_14_H_17_O_4_N_3_Cl [M + H]^+^ 326.0908. Found: 326.0906.

*Furan glucosyl-1,2,3-triazole* (**3-g**). Yield: 79.1%. Oily. ^1^H-NMR (Meth-d_4_): δ 7.86–7.83 (m, 1H, CCHN), 5.25–5.05 (m, 1H, H-1, α and β),4.97–4.46 (m, 4H), 4.05–3.69 (m, 2H), 3.36–3.33 (m, 3H, OMe), 1.44–1.42 (m, 3H, CH-CH_3_); ^13^C-NMR (Meth-d_4_): δ 153.54, 153.49, 123.81, 123.59, 104.76, 97.86, 87.01, 86.63, 80.50, 80.39, 78.80, 79.55, 77.47, 75.84, 63.41, 58.39, 58.31, 58.11, 52.68, 51.82, 23.63. ESI-MS *m/z* calcd. for C_10_H_18_O_5_N_3_ [M + H]^+^ 260.1. Found: 260.1. HRMS for C_10_H_18_O_5_N_3_ [M + H]^+^ 260.1403. Found: 260.1247.

*Furan glucosyl-1,2,3-triazole* (**4-a**). Yield: 80.3%. White solid, m.p. 98.7–99.2 °C. ^1^H-NMR (Meth-d_4_): δ 8.07–7.14 (m, 11H, ArH, CCHN), 5.29–5.10 (m, 1H, H-1, α and β), 4.77–3.92 (m, 7H, H-2, H-3, H-4, H-5, H-6, Ar-CH_2_, α and β); ^13^C-NMR (Meth-d_4_): δ 148.52, 148.46, 139.16, 139.00, 131.65, 131.61, 129.89, 129.47, 129.46, 129.23, 129.10, 129.01, 128.91, 128.88, 126.61, 123.28, 123.11, 104.79, 97.85, 84.96, 84.56, 80.37, 77.46, 76.44, 73.24, 73.15, 52.65, 51.84. ESI-MS *m/z* calcd. for C_20_H_22_O_4_N_3_ [M + H]^+^ 368.1. Found: 368.1. HRMS for C_20_H_22_O_4_N_3_ [M + H]^+^ 368.1610. Found: 368.1608.

*Furan glucosyl-1,2,3-triazole* (**4-b**). Yield: 74.2%. White solid, m.p. 90.2–90.6 °C. ^1^H-NMR (Meth-d_4_): δ 8.03–6.98 (m, 10H, ArH, CCHN), 5.28–5.10 (m, 1H, H-1, α and β), 4.75–3.91 (m, 7H, H-2, H-3, H-4, H-5, H-6, Ar-CH_2_, α and β), 2.22 (s, 3H, Ar-Me); ^13^C-NMR (Meth-d_4_): δ 147.33, 147.27, 138.38, 137.87, 137.71, 130.20, 128.63, 128.50, 128.17, 128.16, 127.79, 127.70, 127.60, 127.58, 125.88, 122.46, 121.94, 121.75, 103.49, 96.55, 83.68, 83.28, 79.07, 76.16, 75.14, 71.94, 71.84, 51.32, 50.51, 20.17. ESI-MS *m/z* calcd. for C_23_H_22_O_4_N_3_ [M + H]^+^ 382.1. Found: 382.1. HRMS for C_23_H_22_O_4_N_3_ [M + H]^+^ 382.1767. Found: 382.1766.

*Furan glucosyl-1,2,3-triazole* (**4-c**). Yield: 76.8%. Yellow solid, m.p. 121.6–123.0 °C. ^1^H-NMR (Meth-d_4_): δ 7.98–6.79 (m, 10H, ArH, CCHN), 5.30–5.11 (m, 1H, H-1, α and β), 4.60–3.92 (m, 7H, H-2, H-3, H-4, H-5, H-6, Ar-CH_2_, α and β), 3.63(s, 3H, Ar-O-Me); ^13^C-NMR (Meth-d_4_): δ 161.23, 139.11, 138.96, 129.45, 129.06, 128.97, 128.89, 128.87, 128.02, 122.42, 115.34, 104.78, 97.88, 84.94, 84.57, 80.31, 77.44, 76.32, 73.22, 73.12, 55.77, 52.76, 51.93. ESI-MS *m/z* calcd. for C_21_H_24_O_5_N_3_ [M + H]^+^ 398.1. Found: 398.1. HRMS for C_21_H_24_O_5_N_3_ [M + H]^+^ 398.1716. Found: 398.1715.

*Furan glucosyl-1,2,3-triazole* (**4-d**). Yield: 80.5%.White solid, m.p. 132.7–134.2 °C. ^1^H-NMR (Meth-d_4_): δ 8.07–6.98 (m, 10H, ArH, CCHN), 5.30–5.10 (m, 1H, H-1, α and β), 5.02–4.04 (m, 7H, H-2, H-3, H-4, H-5, H-6, Ar-CH_2_, α and β); ^13^C-NMR (Meth-d_4_): δ 164.27, 162.82, 139.13, 138.97, 129.48, 129.47, 129.11, 129.01, 128.64, 123.29, 123.10, 116.82, 116.60, 104.80, 97.90, 84.95, 84.57, 80.35, 77.46, 76.38, 73.27, 73.17, 52.78, 51.96. ESI-MS *m/z* calcd. for C_20_H_21_O_4_N_3_F [M + H]^+^ 386.1. Found: 386.1. HRMS for C_20_H_21_O_4_N_3_F [M + H]^+^ 386.1516. Found: 386.1515.

*Furan glucosyl-1,2,3-triazole* (**4-e**). Yield: 81.6%. White solid, m.p. 156.2–156.9 °C. ^1^H-NMR (DMSO): δ 8.80–7.30 (m, 10H, ArH, CCHN), 6.56–6.33 (m, 1H, H-1, α and β), 4.76–4.01 (m, 9H); ^13^C-NMR (DMSO): δ 146.54, 144.21, 138.09, 137.18, 128.26, 127.57, 127.53, 125.82, 124.32, 124.11, 96.30, 96.20, 83.10, 75.34, 74.71, 71.17, 50.53. ESI-MS *m/z* calcd. for C_20_H_21_O_6_N_4_ [M + H]^+^ 413.1. Found: 413.1. HRMS for C_20_H_21_O_6_N_4_ [M + H]^+^ 413.1461. Found: 413.1460.

*Furan glucosyl-1,2,3-triazole* (**4-f**).Yield: 73.4%. White solid, m.p. 103.7–104.4 °C. ^1^H-NMR (Meth-d_4_): δ 8.08–7.15 (m, 10H, ArH, CCHN), 5.29–5.09 (m, 1H, H-1, α and β), 4.74–3.92 (m, 7H, H-2, H-3, H-4, H-5, H-6, Ar-CH_2_, α and β); ^13^C-NMR (Meth-d_4_): δ 147.39, 147.32, 139.17, 139.01, 134.86, 130.01, 129.48, 129.47, 128.03, 123.49, 123.32, 104.80, 97.86, 84.99, 84.58, 80.39, 80.34, 77.44, 76.45, 73.26, 73.16, 52.71, 51.90. ESI-MS *m/z* calcd. for C_20_H_21_O_4_N_3_Cl [M + H]^+^ 367.1. Found: 367.1. HRMS for C_20_H_21_O_4_N_3_Cl [M + H]^+^ 402.1221. Found: 402.1220.

*Furan glucosyl-1,2,3-triazole* (**4-g**). Yield: 79.7%. White solid, m.p. 105.7–106.1 °C. ^1^H-NMR (Meth-d_4_): δ 7.75–7.14 (m, 6H, ArH, CCHN), 5.28–5.08 (m, 1H, H-1, α and β), 4.64–4.05 (m, 8H), 1.59–1.56 (m, 3H, CH-CH_3_); ^13^C-NMR (Meth-d_4_): δ 153.35, 153.26, 139.08, 138.93, 129.41, 128.99, 128.91, 128.89, 123.61, 123.41, 104.65, 97.75, 84.80, 84.78, 84.47, 80.38, 80.20, 77.45, 76.20, 73.13, 73.06, 63.47, 52.52, 51.68, 23.61. ESI-MS *m/z* calcd. for C_16_H_22_O_5_N_3_ [M + H]^+^ 353.1. Found: 353.1. HRMS for C_16_H_22_O_5_N_3_ [M + H]^+^ 353.1907. Found: 353.1906.

*Furan glucosyl-1,2,3-triazole* (**5-a**). Yield: 88.2%. White solid, m.p. 140.2–141 °C. ^1^H-NMR (CDCl_3_): δ 7.87–7.26 (m, 6H, ArH, CCHN), 6.45–6.14 (m, 1H, H-1, α and β), 5.26–4.42 (m, 5H, H-2, H-3, H-4, H-5, H-6, α and β) 3.51–3.45 (m, 3H, OMe, α and β) 2.14–2.03 (m, 6H, Me_2_-CO); ^13^C-NMR (CDCl_3_): δ 169.61, 169.27, 147.96, 130.62, 128.93, 128.27, 127.70, 125.86, 121.15, 120.89, 99.80, 94.06, 82.77, 82.40, 81.79, 78.62, 78.25, 76.16, 58.45, 58.27, 50.36, 49.88, 21.27, 20.91, 20.82, 20.54. ESI-MS *m/z* calcd. for C_18_H_22_O_6_N_3_ [M + H]^+^ 376.1. Found: 376.1. HRMS for C_18_H_22_O_6_N_3_ [M + H]^+^ 376.1509. Found: 376.1507.

*Furan glucosyl-1,2,3-triazole* (**5-b**). Yield: 79.4%. White solid, m.p. 116.7–118.1 °C. ^1^H-NMR (CDCl_3_): δ 7.78–7.04 (m, 5H, ArH, CCHN), 6.38–6.08 (m, 1H, H-1, α and β), 5.19–3.79 (m, 5H, H-2, H-3, H-4, H-5, H-6, α and β) 3.42–3.37 (m, 3H, OMe, α and β) 2.30 (s, 3H, Ar-CH_3_) 2.06–1.95 (m, 6H, Me_2_-CO, α and β); ^13^C-NMR (CDCl_3_): δ 169.52, 169.16, 147.90, 138.46, 130.42, 128.90, 128.71, 126.39, 122.85, 121.04, 120.73, 99.67, 93.93, 82.63, 82.26, 81.72, 78.51, 78.13, 76.09, 58.32, 58.15, 50.22, 49.73, 21.39, 21.15, 20.79, 20.69, 20.43. ESI-MS *m/z* calcd. for C_19_H_24_O_6_N_3_ [M + H]^+^ 390.1. Found: 390.1. HRMS for C_19_H_24_O_6_N_3_ [M + H]^+^ 390.1665. Found: 390.1668.

*Furan glucosyl-1,2,3-triazole* (**5-c**). Yield: 91.9%. White solid, m.p. 156.7–157.9 °C. ^1^H-NMR (CDCl_3_): δ 7.78–6.93 (m, 5H, ArH, CCHN), 6.45–6.15 (m, 1H, H-1, α and β), 5.26–4.40 (m, 5H, H-2, H-3, H-4, H-5, H-6, α and β), 3.82 (m, 3H, Ar-OMe), 3.50–3.45 (m, 3H, OMe), 2.14–2.03 (m, 6H, Me_2_-CO, α and β); ^13^C-NMR (CDCl_3_): δ 169.61, 169.26, 159.74, 147.84, 127.16, 123.40, 120.31, 120.04, 114.36, 99.81, 94.10, 82.74, 82.44, 81.85, 78.62, 78.33, 76.16, 58.43, 58.25, 55.41, 50.25, 49.79, 21.26, 20.90, 20.81, 20.54. ESI-MS *m/z* calcd. for C_18_H_21_O_7_N_3_ [M + H]^+^ 406.1. Found: 406.1. HRMS for C_18_H_21_O_7_N_3_ [M + H]^+^ 406.1614. Found: 406.1612.

*Furan glucosyl-1,2,3-triazole* (**5-d**). Yield: 90.7%. White solid, m.p. 141.6–143.6 °C. ^1^H-NMR (CDCl_3_): δ 7.78–7.00 (m, 5H, ArH, CCHN), 6.38–6.08 (m, 1H, H-1, α and β), 5.20–3.81 (m, 5H, H-2, H-3, H-4, H-5, H-6, α and β), 3.44–3.38 (m, 3H, OMe), 2.07–1.96 (m, 6H, Me_2_-CO, α and β); ^13^C-NMR (CDCl_3_): δ 169.48, 169.43, 169.12, 163.85, 161.39, 146.92, 127.49, 127.41, 120.83, 120.53, 115.84, 115.62, 99.66, 93.88, 82.65, 82.22, 81.66, 78.49, 78.04, 76.06, 58.30, 58.14, 50.32, 49.82, 21.10, 20.74, 20.65, 20.38. ESI-MS *m/z* calcd. for C_18_H_20_O_6_N_3_F [M + H]^+^ 394.1. Found: 394.1. HRMS for C_18_H_20_O_6_N_3_F [M + H]^+^ 394.1414. Found: 394.1414.

*Furan glucosyl-1,2,3-triazole* (**5-e**). Yield: 84.7%. Oily.^1^H-NMR (CDCl_3_): δ 8.27–7.26 (m, 5H, ArH, CCHN), 6.45–6.15 (m, 1H, H-1, α and β), 5.28–3.92 (m, 5H, H-2, H-3, H-4, H-5, H-6, α and β), 3.53–3.47 (m, 3H, OMe, α and β), 2.15–2.03 (m, 6H, Me_2_-CO, α and β); ^13^C-NMR (CDCl_3_): δ 169.61, 169.24, 147.46, 145.81, 136.94, 126.29, 124.38, 122.70, 122.39, 99.82, 93.95, 82.86, 82.31, 81.71, 78.55, 78.00, 76.20, 58.48, 58.33, 50.86, 50.32, 21.26, 20.89, 20.79, 20.53. ESI-MS *m/z* calcd. for C_18_H_21_O_8_N_4_ [M + H]^+^ 421.1. Found: 421.1. HRMS for C_18_H_21_O_8_N_4_ [M + H]^+^ 421.1359. Found: 421.1357.

*Furan glucosyl-1,2,3-triazole* (**5-f**). Yield: 82.9%. White solid, m.p. 131.3–133.0 °C. ^1^H-NMR (CDCl_3_): δ 7.87–7.27 (m, 5H, ArH, CCHN), 6.45–6.15 (m, 1H, H-1, α and β), 5.27–3.90 (m, 5H, H-2, H-3, H-4, H-5, H-6, α and β), 3.52–3.46 (m, 3H, OMe, α and β), 2.15–2.04 (m, 6H, Me_2_-CO, α and β); ^13^C-NMR (CDCl_3_): δ 169.61, 169.56, 169.25, 146.95, 134.02, 129.21, 129.14, 127.12, 121.21, 120.92, 99.82, 94.05, 82.81, 82.40, 81.81, 78.59, 78.21, 76.18, 58.47, 58.29, 50.53, 50.03, 21.28, 20.92, 20.82, 20.55. ESI-MS *m/z* calcd. for C_18_H_21_O_6_N_3_Cl [M + H]^+^ 410.1. Found: 410.1. HRMS for C_18_H_21_O_6_N_3_Cl [M + H]^+^ 410.1119. Found: 410.1120.

*Furan glucosyl-1,2,3-triazole* (**F-a**). Yield: 90.3%. White solid, m.p. 148.0–149.3 °C. ^1^H-NMR (CDCl_3_): δ 7.70–7.21 (m, 11H, ArH, CCHN), 6.40–6.10 (m, 1H, H-1, α and β), 5.25–4.00 (m, 7H, H-2, H-3, H-4, H-5, H-6, Ar-CH_2_, α and β), 2.04–1.93 (m, 6H, Me_2_-CO); ^13^C-NMR (CDCl_3_): δ 169.50, 169.16, 147.71, 136.93, 136.73, 130.54, 128.77, 128.67, 128.61, 128.33, 128.24, 128.10, 128.01, 127.98, 125.72, 121.16, 120.89, 99.70, 94.03, 81.67, 80.12, 80.05, 78.92, 78.12, 76.48, 72.43, 72.18, 50.35, 49.86, 21.13, 20.78, 20.69, 20.42. ESI-MS *m/z* calcd. for C_24_H_26_O_6_N_3_ [M + H]^+^ 452.1. Found: 452.1. HRMS for C_24_H_26_O_6_N_3_ [M + H]^+^ 452.1822. Found: 452.1821.

*Furan glucosyl-1,2,3-triazole* (**6-b**). Yield: 91.6%. White solid, m.p. 110.7–112.5 °C. ^1^H-NMR (CDCl_3_): δ 7.67–6.95 (m, 10H, ArH, CCHN), 6.35–6.06 (m, 1H, H-1, α and β), 5.20–3.96 (m, 7H, H-2, H-3, H-4, H-5, H-6, Ar-CH_2_, α and β) 2.22 (s, 3H, Ar-CH_3_), 1.97–1.86 (m, 6H, Me_2_-CO, α and β); ^13^C-NMR (CDCl_3_): δ 169.25, 168.90, 147.43, 138.12, 136.84, 136.65, 130.28, 128.58, 128.44, 128.38, 128.33, 128.00, 127.92, 127.75, 127.70, 126.08, 122.58, 120.98, 120.68, 99.40, 93.72, 81.43, 79.97, 79.79, 78.70, 76.84, 76.30, 72.16, 71.91, 50.09, 49.58, 21.13, 20.84, 20.49, 20.40, 20.14. ESI-MS *m/z* calcd. for C_25_H_28_O_6_N_3_ [M + H]^+^ 466.1. Found: 466.1. HRMS for C_25_H_28_O_6_N_3_ [M + H]^+^ 466.1978. Found: 466.1980.

*Furan glucosyl-1,2,3-triazole* (**6-c**). Yield: 92.5%. Yellow solid, m.p. 140.6–141.9 °C. ^1^H-NMR (CDCl_3_): δ 7.89–6.85 (m, 10H, ArH, CCHN), 6.49–6.19 (m, 1H, H-1, α and β), 5.24–4.03 (m, 7H, H-2, H-3, H-4, H-5, H-6, Ar-CH_2_, α and β), 3.83 (s, 3H, Ar-CH_3_), 2.11–2.04 (m, 6H, Me_2_-CO, α and β); ^13^C-NMR (CDCl_3_): δ 170.24, 169.87, 159.71, 159.66, 147.61, 147.55, 137.09, 137.06, 128.78, 128.72, 128.69, 128.31, 128.28, 128.06, 127.11, 123.51, 120.28, 120.12, 114.32, 114.29, 108.00, 100.77, 94.18, 80.80, 80.40, 80.34, 79.89, 78.62, 75.68, 72.51, 72.28, 55.40, 50.33, 49.90, 20.92, 20.81, 20.74, 20.54. ESI-MS *m/z* calcd. for C_25_H_28_O_7_N_3_ [M + H]^+^ 482.1. Found: 482.1. HRMS for C_25_H_28_O_7_N_3_ [M + H]^+^ 482.1927. Found: 482.1927.

*Furan glucosyl-1,2,3-triazole* (**6-d**). Yield: 87.1%. White solid, m.p. 123.8–124.9 °C. ^1^H-NMR (CDCl_3_): δ 7.68–6.98 (m, 10H, ArH, CCHN), 6.41–6.11 (m, 1H, H-1, α and β), 5.26–4.01 (m, 7H, H-2, H-3, H-4, H-5, H-6, Ar-CH_2_, α and β), 2.06–1.95 (m, 6H, Me_2_-CO); ^13^C-NMR (CDCl_3_): δ 169.66, 169.58, 169.49, 169.24, 163.93, 161.47, 146.96, 146.94, 136.94, 136.74, 128.75, 128.70, 128.43, 128.34, 128.08, 128.06, 127.57, 127.48, 120.97, 120.71, 115.91, 115.69, 99.77, 94.08, 81.74, 80.15, 80.08, 78.96, 78.16, 76.53, 72.51, 72.24, 50.51, 50.02, 21.20, 20.85, 20.77, 20.49. ESI-MS *m/z* calcd. for C_24_H_25_O_6_N_3_F [M + H]^+^ 470.1. Found: 470.1. HRMS for C_24_H_25_O_6_N_3_F [M + H]^+^: 470.1727. Found: 470.1736.

*Furan glucosyl-1,2,3-triazole* (**6-e**). Yield: 89.4%. Oily. ^1^H-NMR (CDCl_3_): δ 8.18–7.26 (m, 10H, ArH, CCHN), 6.42–6.12 (m, 1H, H-1, α and β), 5.28–4.06 (m, 7H, H-2, H-3, H-4, H-5, H-6, Ar-CH_2_, α and β) 2.07–1.96 (m, 6H, Me_2_-CO); ^13^C-NMR (CDCl_3_): δ 169.61, 169.25, 147.36, 145.63, 136.89, 136.86, 136.69, 128.79, 128.73, 128.49, 128.40, 128.09, 128.07, 126.22, 126.20, 124.28, 122.82, 122.53, 99.78, 93.98, 81.61, 80.21, 80.00, 78.94, 77.90, 76.54, 72.57, 72.28, 50.85, 50.34, 21.21, 20.86, 20.77, 20.49. ESI-MS *m/z* calcd. for C_24_H_25_O_8_N_4_ [M + H]^+^ 497.1. Found: 497.1. HRMS for C_24_H_25_O_8_N_4_ [M + H]^+^: 497.1672. Found: 497.1684.

*Furan glucosyl-1,2,3-triazole* (**6-f**). Yield: 92.8%. White solid, m.p. 140.6–141.4 °C. ^1^H-NMR (CDCl_3_): δ 7.78–7.31 (m, 10H, ArH, CCHN), 6.47–6.18 (m, 1H, H-1, α and β), 5.33–4.09 (m, 7H, H-2, H-3, H-4,H-5,H-6, Ar-CH_2_, α and β), 2.11–2.01 (m, 6H, Me_2_-CO); ^13^C-NMR (CDCl_3_): δ 169.49, 169.14, 149.62, 146.67, 136.91, 136.71, 136.11, 133.79, 129.15, 128.97, 128.70, 128.64, 128.37, 128.28, 128.02, 128.00, 126.98, 123.79, 121.25, 120.97, 99.71, 94.00, 81.65, 80.15, 80.03, 78.92, 78.04, 76.49, 72.47, 72.20, 50.48, 49.99, 21.14, 20.79, 20.70, 20.43. ESI-MS *m/z* calcd. for C_24_H_25_O_6_N_3_Cl [M + H]^+^ 486.1. Found: 486.1. HRMS for C_24_H_25_O_6_N_3_Cl [M + H]^+^ 486.1432. Found: 486.1437.

### 3.3. Fungicidal Assays

The fungicidal activity was determined by mycelium growth rate test as previously reported [25]. Each of the test compounds was dissolved in DMSO (10 mL). The culture media, with known concentration of the test compounds, were obtained by mixing the soln of compounds **1**–**6** in DMSO with potato dextrose agar (PDA), on which fungus cakes were placed. The blank test was made using DMSO. The commercial fungicide chlorothalonil was used as a control in the above bioassay. The culture was carried out at 24 ± 0.5 °C. Three replicates were performed. Inhibition rates of compounds **1**–**6** against *P. CapasiciLeonian*, *Sclerotinia sclerotiorum* (Lib.) de Bary, *B. cinerea, Pyricularia oryzae* Cav. and *Fusarium oxysporum Schl. F.sp. vasinfectum* (Atk.) Snyd. & Hans. at 50 μg/mL were given in Table 2.

### 3.4. Enzyme Inhibitory Activities Bioassay

Inhibitory activity of all the synthesized compounds towards Candida albicans GlcN-6-P synthase was determined using the optimized Elson-Morgan method as previously reported [25]. Absorbance at λ = 585 nm was measured and GlcN-6-P concentration in the sample was read from the standard curve (Solutions of glucosamine-HCl (0.1–1 mM) were assayed simultaneously, to obtain a standard line from the plot of extinction against concentration of glucosamine). In each experiment, two control samples, one without enzyme and one without substrates, were assayed in the same way. Three replicates were performed. The inhibition rates were given in Table 3 at 0.35 mm.

## 4. Conclusions

In summary, forty compounds of triazole were synthesized in an efficient and practical way, thirty-nine of them were new compounds, and the bioactivities of all the compounds were evaluated. The bioassays showed that they had the inhibitory activities against glucosamine-6-phosphate synthase, at the same times, most of them also exhibited good fungicidal activity against *P. CapasiciLeonian*, *Sclerotinia sclerotiorum* (Lib.) de Bary, *Pyricularia oryzae* Cav. and *Fusarium oxysporum Schl. F.sp. vasinfectum* (Atk.) Snyd. & Hans. Especially the compounds **1-d** and **1-f** displayed good fungicidal activities against *Sclerotinia sclerotiorum* (Lib.) de Bary and *Fusarium oxysporum Schl. F.sp. vasinfectum* (Atk.) Snyd. & Hans. They were close to the commercial fungicide chlorothalonil. In the same series, the compounds which exhibited good fungicidal activities were consistent with their glucosamine-6-phosphate synthase inhibitory activities, and the benzene ring with electron-withdrawing substituents (such as fluorine, chlorine, nitro) made the activity increased. The compounds with the OH-protection at both 1 and 2-position in sugar ring exhibited better fungicidal activities than those without protection, but had less inhibitory activities against Glms, which may be associated with a better structural similarity between fructose-6-phosphate and the compounds without protection. Further studies are in progress. 

## Figures and Tables

**Table 1:** Physical Data of Compounds **1**, **2**, **3**, **4**, **5** and **6**.

Compd.	R^1^	R^2^	Formula	Status	m.p./°C	Yield (%)
**1-a**	Me	C_6_H_5_-	C_17_H_21_O_4_N_3_	White foamy solid	159.9–160.4	79.4
**1-b**	Me	3-CH_3_-C_6_H_4_-	C_18_H_23_O_4_N_3_	Yellow foamy solid	108.0–109.7	79.6
**1-c**	Me	4-CH_3_O-C_6_H_4_-	C_18_H_23_O_5_N_3_	White foamy solid	156.6–157.5	69.3
**1-d**	Me	4-F-C_6_H_4_-	C_17_H_20_O_4_N_3_F	White foamy solid	121.4–122.3	83.0
**1-e**	Me	4-NO_2_-C_6_H_4_-	C_17_H_20_O_6_N_4_	White foamy solid	126.5–126.8	77.1
**1-f**	Me	4-Cl-C_6_H_4_-	C_17_H_20_O_4_N_3_Cl	White foamy solid	135.9–136.7	79.3
**1-g**	Me	CH_3_-CH(OH)-	C_13_H_21_O_5_N_3_	Yellow foamy solid	90.2–90.9	92.3
**2-a** [24]	Bn	C_6_H_5_-	C_23_H_25_O_4_N_3_	White foamy solid	133.2–134.1	66.0
**2-b**	Bn	3-CH_3_-C_6_H_4_-	C_24_H_27_O_4_N_3_	White foamy solid	108.8–110.2	79.4
**2-c**	Bn	4-CH_3_O-C_6_H_4_-	C_24_H_27_O_5_N_3_	White foamy solid	134.6–135.7	73.6
**2-d**	Bn	4-F-C_6_H_4_-	C_23_H_24_O_4_N_3_F	White foamy solid	164.3–164.7	90.4
**2-e**	Bn	4-NO_2_-C_6_H_4_-	C_23_H_24_O_6_N_4_	Yellow foamy solid	182.6–183.6	90.3
**2-f**	Bn	4-Cl-C_6_H_4_-	C_23_H_24_O_4_N_3_Cl	White foamy solid	136.2–136.5	93.7
**2-g**	Bn	CH_3_-CH(OH)-	C_19_H_25_O_5_N_3_	White foamy solid	90.6–90.9	87.6
**3-a**	Me	C_6_H_5_-	C_14_H_17_O_4_N_3_	White foamy solid	101.1–102.1	78.5
**3-b**	Me	3-CH_3_-C_6_H_4_-	C_15_H_19_O_4_N_3_	Oily	——	77.4
**3-c**	Me	4-CH_3_O-C_6_H_4_-	C_15_H_29_O_5_N_3_	White foamy solid	130.3–130.8	79.4
**3-d**	Me	4-F-C_6_H_4_-	C_14_H_16_O_4_N_3_F	White foamy solid	136.5–137.1	64.9
**3-e**	Me	4-NO_2_-C_6_H_4_-	C_14_H_16_O_6_N_4_	White foamy solid	149.2–150.1	74.3
**3-f**	Me	4-Cl-C_6_H_4_-	C_14_H_16_O_4_N_3_Cl	White foamy solid	109.4–110.2	80.7
**3-g**	Me	CH_3_-CH(OH)-	C_10_H_16_O_5_N_3_	Oily	——	60.2
**4-a**	Bn	C_6_H_5_-	C_20_H_21_O_4_N_3_	White foamy solid	98.7–99.6	80.3
**4-b**	Bn	3-CH_3_-C_6_H_4_-	C_23_H_21_O_4_N_3_	White foamy solid	90.2–90.6	74.2
**4-c**	Bn	4-CH_3_O-C_6_H_4_-	C_21_H_23_O_5_N_3_	Yellow foamy solid	121.6–123.0	76.8
**4-d**	Bn	4-F-C_6_H_4_-	C_20_H_20_O_4_N_3_F	White foamy solid	132.7–134.2	80.5
**4-e**	Bn	4-NO_2_-C_6_H_4_-	C_20_H_20_O_6_N_4_	White foamy solid	156.2–156.9	81.6
**4-f**	Bn	4-Cl-C_6_H_4_-	C_20_H_20_O_4_N_3_Cl	Yellow foamy solid	103.7–104.4	73.4
**4-g**	Bn	CH_3_-CH(OH)-	C_16_H_21_O_5_N_3_	White foamy solid	105.7–106.1	87.6
**5-a**	Me	C_6_H_5_-	C_18_H_21_O_6_N_3_	White foamy solid	140.2–141.1	88.9
**5-b**	Me	3-CH_3_-C_6_H_4_-	C_19_H_23_O_6_N_3_	White foamy solid	116.7–118.1	87.4
**5-c**	Me	4-CH_3_O-C_6_H_4_-	C_18_H_20_O_7_N_3_	White foamy solid	156.7–157.9	89.4
**5-d**	Me	4-F-C_6_H_4_-	C_18_H_20_O_6_N_3_F	White foamy solid	141.6–143.6	85.4
**5-e**	Me	4-NO_2_-C_6_H_4_-	C_18_H_20_O_8_N_4_	Oily	——	80.3
**5-f**	Me	4-Cl-C_6_H_4_-	C_18_H_20_O_6_N_3_Cl	White foamy solid	131.3–133.0	90.7
**6-a**	Bn	C_6_H_5_-	C_24_H_25_O_6_N_3_	White foamy solid	148.0–149.3	87.2
**6-b**	Bn	3-CH_3_-C_6_H_4_-	C_25_H_27_O_6_N_3_	White foamy solid	110.7–112.5	82.4
**6-c**	Bn	4-CH_3_O-C_6_H_4_-	C_25_H_27_O_7_N_3_	Yellow foamy solid	140.6–141.9	86.7
**6-d**	Bn	4-F-C_6_H_4_-	C_24_H_24_O_6_N_3_F	White foamy solid	123.8–124.9	85.3
**6-e**	Bn	4-NO_2_-C_6_H_4_-	C_24_H_24_O_8_N_4_	Oily	——	86.1
**6-f**	Bn	4-Cl-C_6_H_4_-	C_24_H_24_O_6_N_3_Cl	White foamy solid	140.6–141.4	88.7

**Table 2:** Inhibition rate of target compounds against five fungus species (% control at 50 µg/mL).

Compd.	Inhibition Ratio (%)				
	*P. CapasiciLeonian*	*S. sclerotiorum*	*B. cinerea*	*Pyricularia oryzae* Cav.	*Fusarium oxysporum Schl. F.sp. vasinfectum* (Atk.) Snyd. & Hans.
**1-a**	60.7	70.2	45.2	72.6	70.1
**1-b**	61.3	69.4	50.2	55.7	50.4
**1-c**	60.7	71.5	49.1	67.2	65.1
**1-d**	80.1	87.6	62.9	81.7	85.6
**1-e**	82.5	83.9	56.2	51.7	65.8
**1-f**	81.2	85.1	71.0	82.4	89.0
**1-g**	40.2	49.2	48.7	43.6	52.7
**2-a** [24]	48.2	66.6	69.6	73.1	68.1
**2-b**	65.3	69.0	53.1	59.6	46.8
**2-c**	59.8	62.8	45.5	60.4	68.5
**2-d**	76.6	88.1	65.9	57.2	67.3
**2-e**	84.9	82.5	60.3	52.2	64.1
**2-f**	86.5	85.3	67.9	60.2	67.9
**2-g**	50.1	43.9	41.3	51.2	46.7
**3-a**	40.1	30.3	33.3	61.2	58.7
**3-b**	35.6	28.4	15.3	35.4	34.1
**3-c**	38.9	31.5	10.4	30.1	29.3
**3-d**	50.2	38.4	19.1	47.8	40.2
**3-e**	44.7	39.6	17.1	23.1	25.6
**3-f**	46.8	25.3	20.5	50.7	57.6
**3-g**	23.1	13.7	11.5	21.5	23.4
**4-a**	21.2	26.4	19.5	37.8	35.4
**4-b**	29.6	29.9	10.9	27.8	27.1
**4-c**	30.3	30.5	11.2	32.3	33.1
**4-d**	35.4	34.7	20.3	35.6	37.8
**4-e**	39.1	32.1	21.1	30.0	31.9
**4-f**	38.7	31.7	22.2	40.2	43.1
**4-g**	24.5	13.5	12.1	21.3	20.9
**5-a**	42.3	57.6	36.1	62.7	61.3
**5-b**	37.6	48.5	23.7	42.3	39.2
**5-c**	47.2	43.1	19.2	40.8	30.0
**5-d**	65.2	67.6	23.2	69.2	70.9
**5-e**	67.7	64.9	22.1	56.9	69.5
**5-f**	68.2	56.1	27.6	60.5	71.5
**6-a**	23.1	46.7	23.5	32.0	35.9
**6-b**	30.0	39.8	17.1	42.3	40.8
**6-c**	31.2	42.8	18.6	28.7	37.6
**6-d**	57.1	64.7	26.7	50.2	58.6
**6-e**	50.3	56.3	27.4	48.9	59.3
**6-f**	51.6	41.2	29.1	40.8	57.8
Chlorothalonil	94.2	95.5	98.0	89.2	94.2

**Table 3:** Enzyme inhibition Rate of Compounds **1**, **2**, **3**, **4**, **5** and **6** at 0.35 mm.

Compd.	Inhibition Rate (%)	Compd.	Inhibition Rate (%)	Compd.	Inhibition Rate (%)	Compd.	Inhibition Rate (%)
**1-a**	15.2	**2-d**	15.5	**3-g**	31.1	**5-c**	29.7
**1-b**	16.1	**2-e**	18.3	**4-a**	26.1	**5-d**	27.9
**1-c**	11.6	**2-f**	12.8	**4-b**	30.5	**5-e**	24.0
**1-d**	17.5	**2-g**	17.8	**4-c**	19.4	**5-f**	30.3
**1-e**	16.0	**3-a**	35.8	**4-d**	27.9	**6-a**	11.2
**1-f**	26.3	**3-b**	27.4	**4-e**	21.4	**6-b**	21.1
**1-g**	10.3	**3-c**	28.1	**4-f**	28.7	**6-c**	19.4
**2-a**	11.1	**3-d**	33.6	**4-g**	29.6	**6-d**	23.5
**2-b**	14.9	**3-e**	37.9	**5-a**	25.7	**6-e**	25.3
**2-c**	12.4	**3-f**	44.1	**5-b**	23.2	**6-f**	20.1

## Supplementary Materials

Supplementary File 1
