# Supplementary material for: Efficient Synthesis and Bioactivity of Novel Triazole Derivatives
Source: Molecules. 2018 Mar 21;23(4):709. doi: 10.3390/molecules23040709 (PMC6017136; doi:10.3390/molecules23040709)

Efficient Synthesis and Bioactivity of Novel Triazole Derivatives

Boyang Hu, Hanqing Zhao *, Zili Chen, Chen Xu, Jianzhuang Zhao and Wenting Zhao

Key Laboratory of Urban Agriculture (North China), Ministry of Agriculture, College of Biological Science and Engineering, Beijing University of Agriculture, Beijing 102206, China; Huboyang_BUA@163.com (B.H.); chenzili1995@163.com (Z.C.); 13691458370@163.com (C.X.); zhaojianzhuang@263.net (J.Z.); wendyz0518@bua.edu.cn (W.Z.)

* Correspondence: zhaohanqing@bua.edu.cn; Tel.: +86-10-8079-9234; Fax: +86-10-8079-9234

Supporting Information

Page No.

Spectrum section

1 ^{1}H NMR spectrum of compound **1-a** S-5

2 ^{13}C NMR spectrum of compound **1-a** S-5

3 HRMS spectrum of compound **1-a** S-5

4 ^{1}H NMR spectrum of compound **1-b** S-6

5 ^{13}C NMR spectrum of compound **1-b** S-6

6 HRMS spectrum of compound **1-b** S-6

7 ^{1}H NMR spectrum of compound **1-c** S-7

8 ^{13}C NMR spectrum of compound **1-c** S-7

9 HRMS spectrum of compound **1-c** S-7

10 ^{1}H NMR spectrum of compound **1-d** S-8

11 ^{13}C NMR spectrum of compound **1-d** S-8

12 HRMS spectrum of compound **1-d** S-8

13 ^{1}H NMR spectrum of compound **1-e** S-9

14 ^{13}C NMR spectrum of compound **1-e** S-9

15 HRMS spectrum of compound **1-e** S-9

16 ^{1}H NMR spectrum of compound **1-f** S-10

17 ^{13}C NMR spectrum of compound **1-f** S-10

18 HRMS spectrum of compound **1-f** S-10

19 ^{1}H NMR spectrum of compound **1-g** S-11

20 ^{13}C NMR spectrum of compound **1-g** S-11

21 HRMS spectrum of compound **1-g** S-11

22 ^{1}H NMR spectrum of compound **2-a** S-12

23 ^{13}C NMR spectrum of compound **2-a** S-12

24 HRMS spectrum of compound **2-a** S-12

25 ^{1}H NMR spectrum of compound **2-b** S-13

26 ^{13}C NMR spectrum of compound **2-b** S-13

27 HRMS spectrum of compound **2-b** S-13

28 ^{1}H NMR spectrum of compound **2-c** S-14

29 ^{13}C NMR spectrum of compound **2-c** S-14

30 HRMS spectrum of compound **2-c** S-14

31 ^{1}H NMR spectrum of compound **2-d** S-15

32 ^{13}C NMR spectrum of compound **2-d** S-15

33 HRMS spectrum of compound **2-d** S-15

34 ^{1}H NMR spectrum of compound **2-e** S-16

35 ^{13}C NMR spectrum of compound **2-e** S-16

36 HRMS spectrum of compound **2-e** S-16

37 ^{1}H NMR spectrum of compound **2-f** S-17

38 ^{13}C NMR spectrum of compound **2-f** S-17

39 HRMS spectrum of compound **2-f** S-17

40 ^{1}H NMR spectrum of compound **2-g** S-18

41 ^{13}C NMR spectrum of compound **2-g** S-18

42 HRMS spectrum of compound **2-g** S-18

43 ^{1}H NMR spectrum of compound **3-a** S-19

44 ^{13}C NMR spectrum of compound **3-a** S-19

45 HRMS spectrum of compound **3-a** S-19

46 ^{1}H NMR spectrum of compound **3-b** S-20

47 ^{13}C NMR spectrum of compound **3-b** S-20

48 HRMS spectrum of compound **3-b** S-20

49 ^{1}H NMR spectrum of compound **3-c** S-21

50 ^{13}C NMR spectrum of compound **3-c** S-21

51 HRMS spectrum of compound **3-c** S-21

52 ^{1}H NMR spectrum of compound **3-d** S-22

53 ^{13}C NMR spectrum of compound **3-d** S-22

54 HRMS spectrum of compound **3-d** S-22

55 ^{1}H NMR spectrum of compound **3-e** S-23

56 ^{13}C NMR spectrum of compound **3-e** S-23

57 HRMS spectrum of compound **3-e** S-23

58 ^{1}H NMR spectrum of compound **3-f** S-24

59 ^{13}C NMR spectrum of compound **3-f** S-24

60 HRMS spectrum of compound **3-f** S-24

61 ^{1}H NMR spectrum of compound **3-g** S-25

62 ^{13}C NMR spectrum of compound **3-g** S-25

63 HRMS spectrum of compound **3-g** S-25

64 ^{1}H NMR spectrum of compound **4-a** S-26

65 ^{13}C NMR spectrum of compound **4-a** S-26

66 HRMS spectrum of compound **4-a** S-26

67 ^{1}H NMR spectrum of compound **4-b** S-27

68 ^{13}C NMR spectrum of compound **4-b** S-27
69 HRMS spectrum of compound **4-b** S-27
70 ^{1}H NMR spectrum of compound **4-c** S-28
71 ^{13}C NMR spectrum of compound **4-c** S-28
72 HRMS spectrum of compound **4-c** S-28
73 ^{1}H NMR spectrum of compound **4-d** S-29
74 ^{13}C NMR spectrum of compound **4-d** S-29
75 HRMS spectrum of compound **4-d** S-29
76 ^{1}H NMR spectrum of compound **4-e** S-30
77 ^{13}C NMR spectrum of compound **4-e** S-30
78 HRMS spectrum of compound **4-e** S-30
79 ^{1}H NMR spectrum of compound **4-f** S-31
80 ^{13}C NMR spectrum of compound **4-f** S-31
81 HRMS spectrum of compound **4-f** S-31
82 ^{1}H NMR spectrum of compound **4-g** S-32
83 ^{13}C NMR spectrum of compound **4-g** S-32
84 HRMS spectrum of compound **4-g** S-32
85 ^{1}H NMR spectrum of compound **5-a** S-33
86 ^{13}C NMR spectrum of compound **5-a** S-33
87 HRMS spectrum of compound **5-a** S-33
88 ^{1}H NMR spectrum of compound **5-b** S-34
89 ^{13}C NMR spectrum of compound **5-b** S-34
90 HRMS spectrum of compound **5-b** S-34
91 ^{1}H NMR spectrum of compound **5-c** S-35
92 ^{13}C NMR spectrum of compound **5-c** S-35
93 HRMS spectrum of compound **5-c** S-35
94 ^{1}H NMR spectrum of compound **5-d** S-36
95 ^{13}C NMR spectrum of compound **5-d** S-36
96 HRMS spectrum of compound **5-d** S-36
97 ^{1}H NMR spectrum of compound **5-e** S-37
98 ^{13}C NMR spectrum of compound **5-e** S-37
99 HRMS spectrum of compound **5-e** S-37
100 ^{1}H NMR spectrum of compound **5-f** S-38
101 ^{13}C NMR spectrum of compound **5-f** S-38
102 HRMS spectrum of compound **5-f** S-38
103 ^{1}H NMR spectrum of compound **6-a** S-39
104 ^{13}C NMR spectrum of compound **6-a** S-39
105 HRMS spectrum of compound **6-a** S-39
106 ^{1}H NMR spectrum of compound **6-b** S-40

107 ^{13}C NMR spectrum of compound **6-b** S-40
108 HRMS spectrum of compound **6-b** S-40
109 ^{1}H NMR spectrum of compound **6-c** S-41
110 ^{13}C NMR spectrum of compound **6-c** S-41
111 HRMS spectrum of compound **6-c** S-41
112 ^{1}H NMR spectrum of compound **6-d** S-42
113 ^{13}C NMR spectrum of compound **6-d** S-42
114 HRMS spectrum of compound **6-d** S-42
115 ^{1}H NMR spectrum of compound **6-e** S-43
116 ^{13}C NMR spectrum of compound **6-e** S-43
117 HRMS spectrum of compound **6-e** S-43
118 ^{1}H NMR spectrum of compound **6-f** S-44
119 ^{13}C NMR spectrum of compound **6-f** S-44
120 HRMS spectrum of compound **6-f** S-44

^{1}H NMR spectrum of compound 1-a

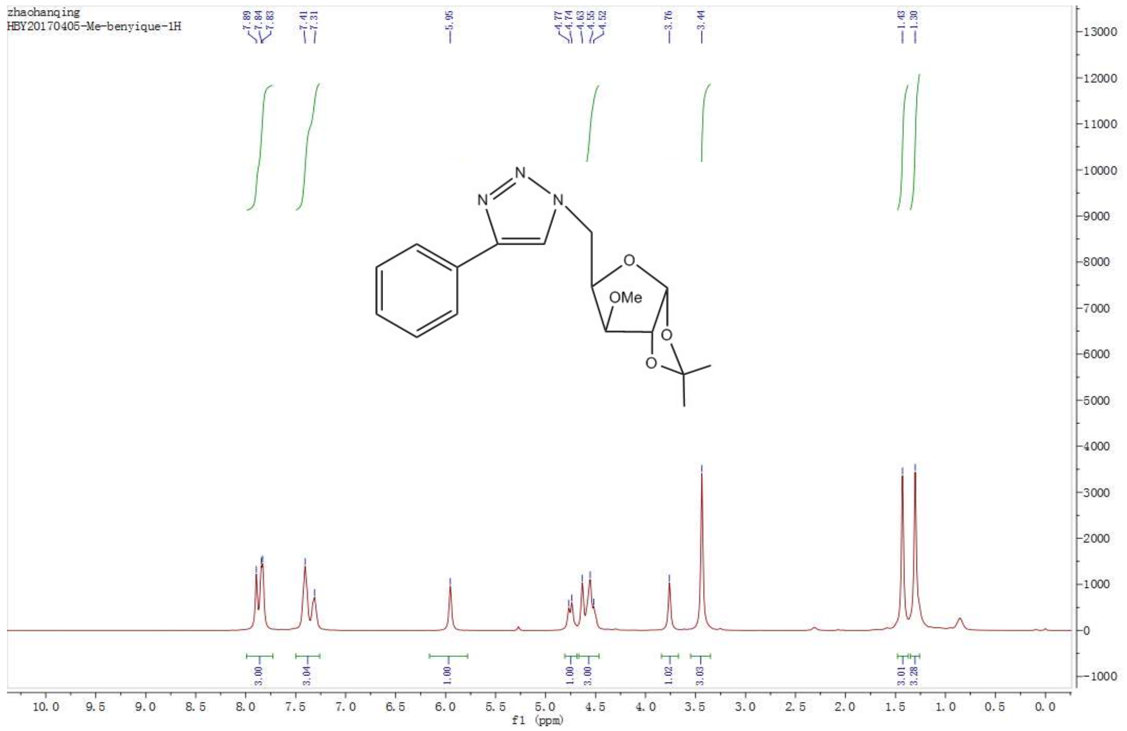

^{13}C NMR spectrum of compound 1-a

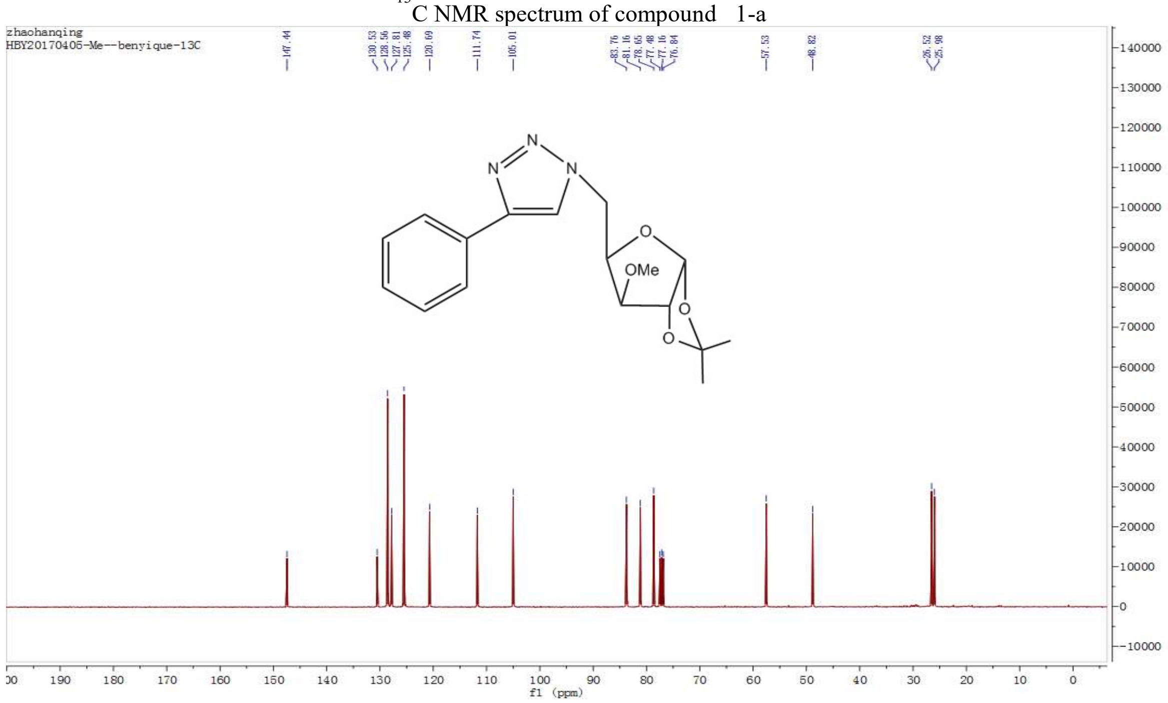

HRMS spectrum of compound **1-a**

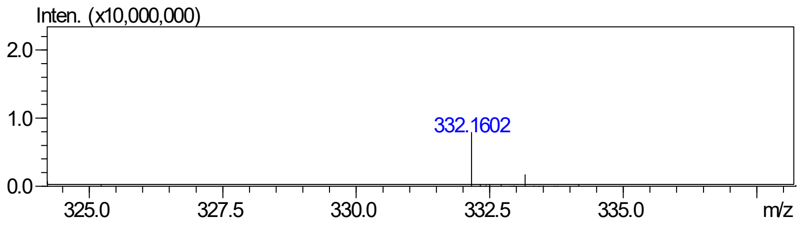

^{1}H NMR spectrum of compound **1-b**

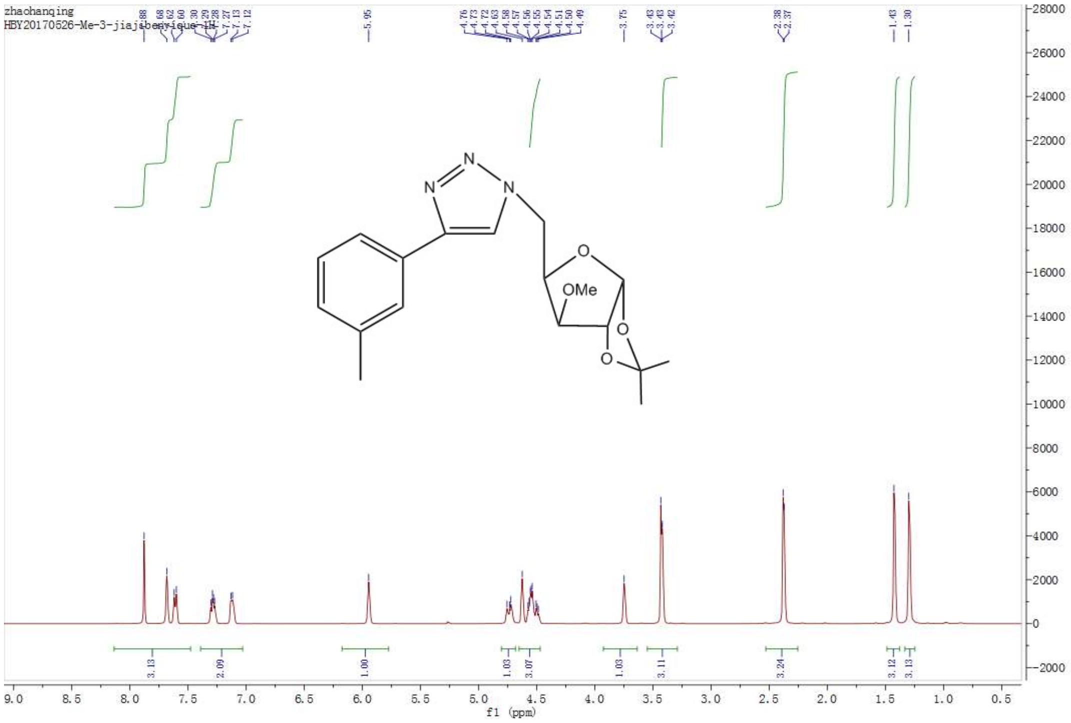

^{13}C NMR spectrum of compound **1-b**

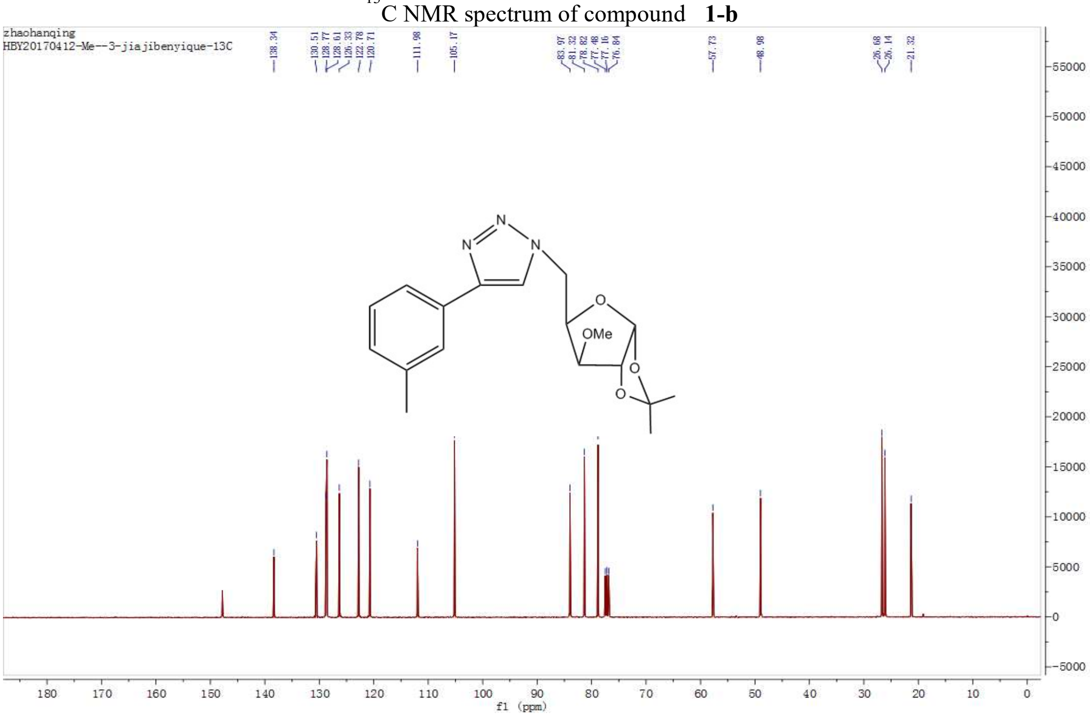

HRMS spectrum of compound **1-b**

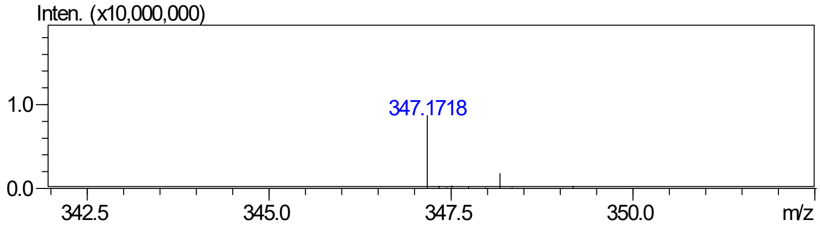

^{1}H NMR spectrum of compound 1-c

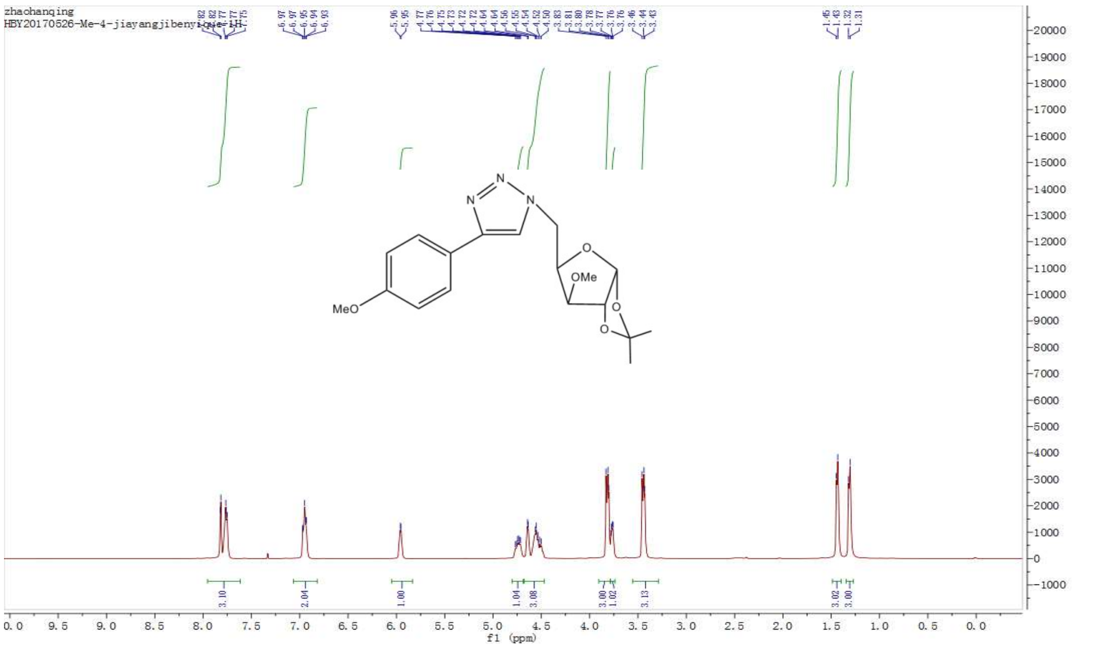

^{13}C NMR spectrum of compound 1-c

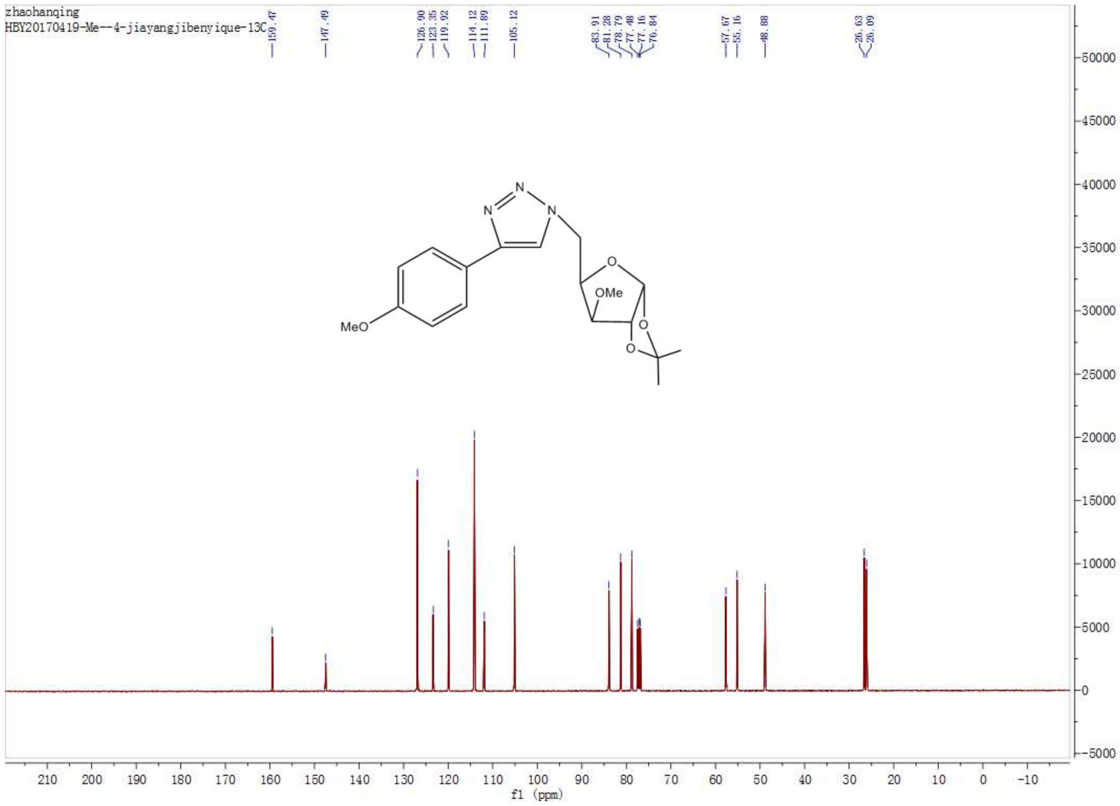

HRMS spectrum of compound 1-c

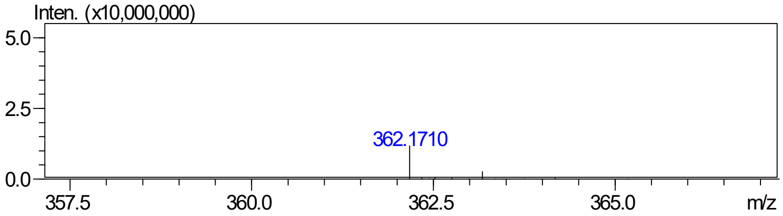

^{1}H NMR spectrum of compound 1-d

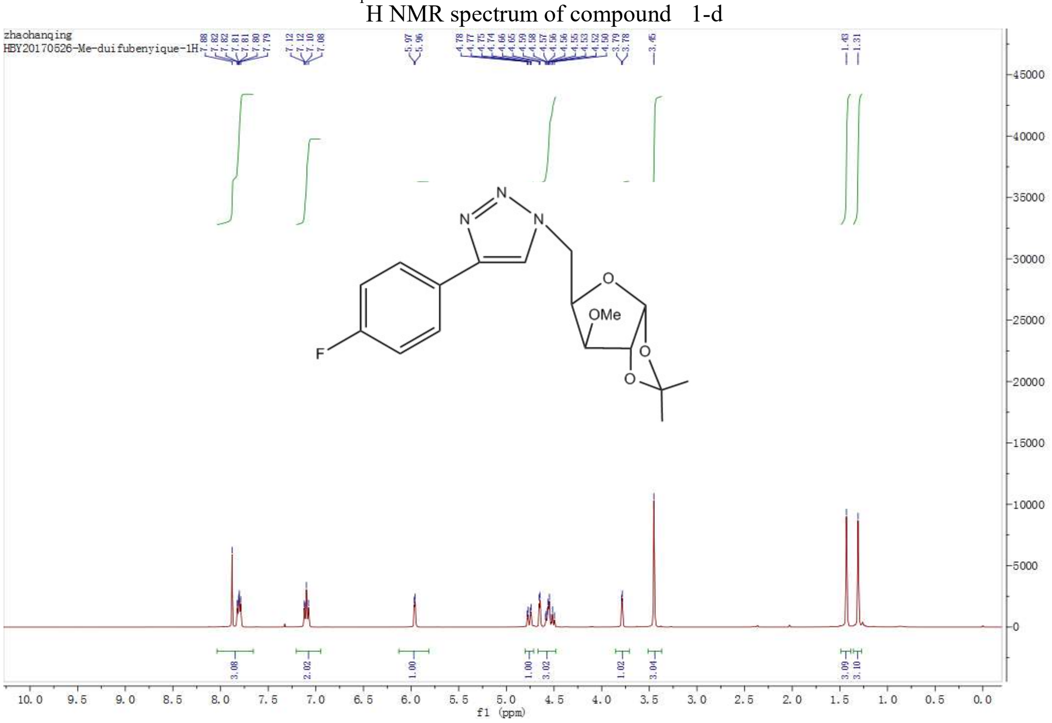

^{13}C NMR spectrum of compound 1-d

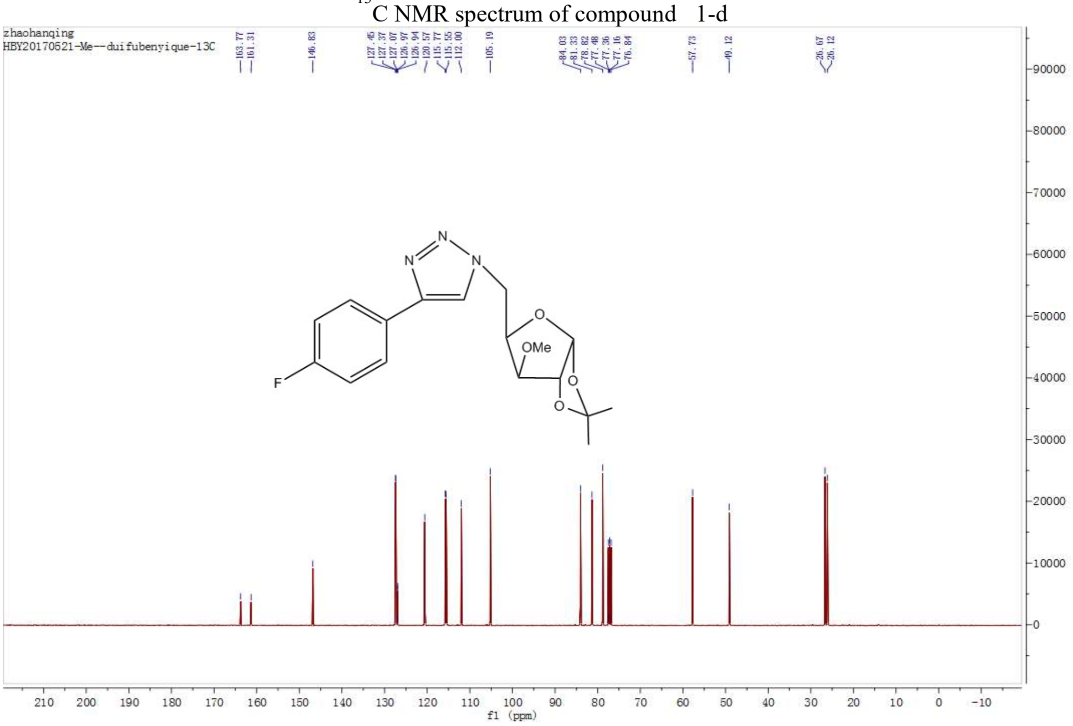

HRMS spectrum of compound 1-d

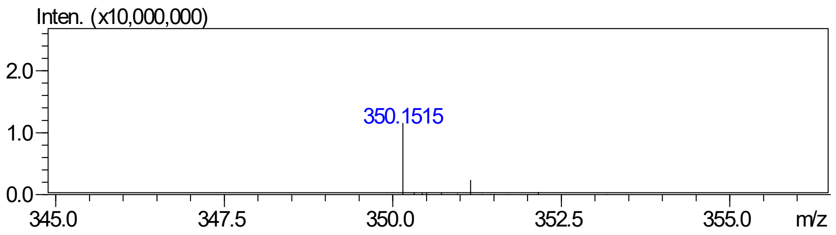

^{1}H NMR spectrum of compound 1-e

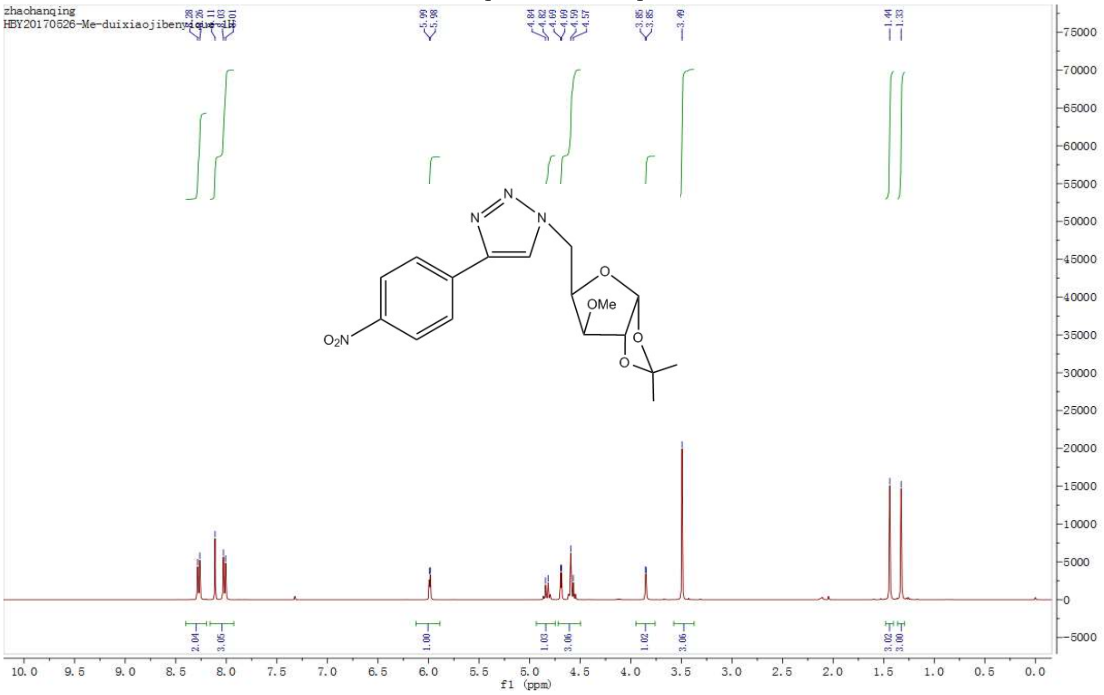

^{13}C NMR spectrum of compound 1-e

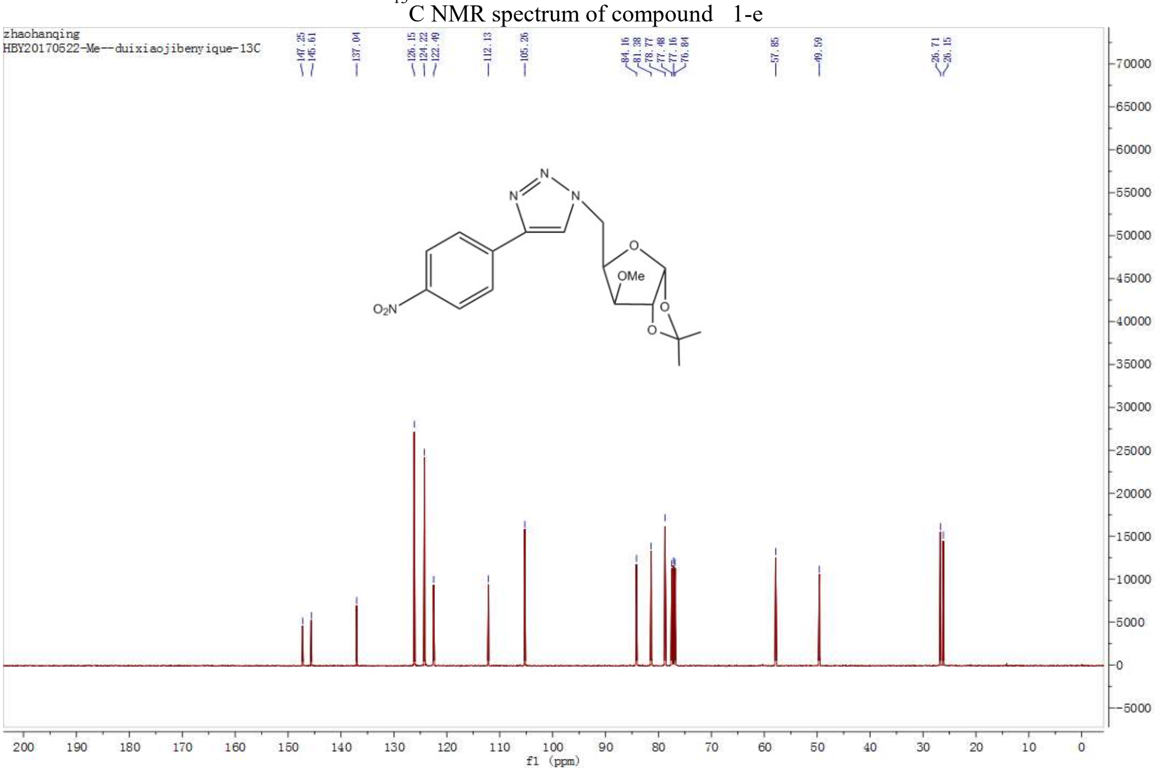

HRMS spectrum of compound 1-e

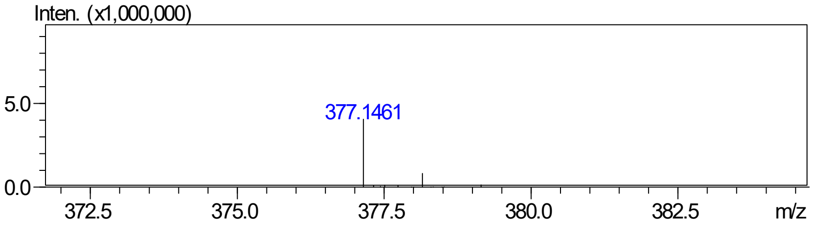

^{1}H NMR spectrum of compound 1-f

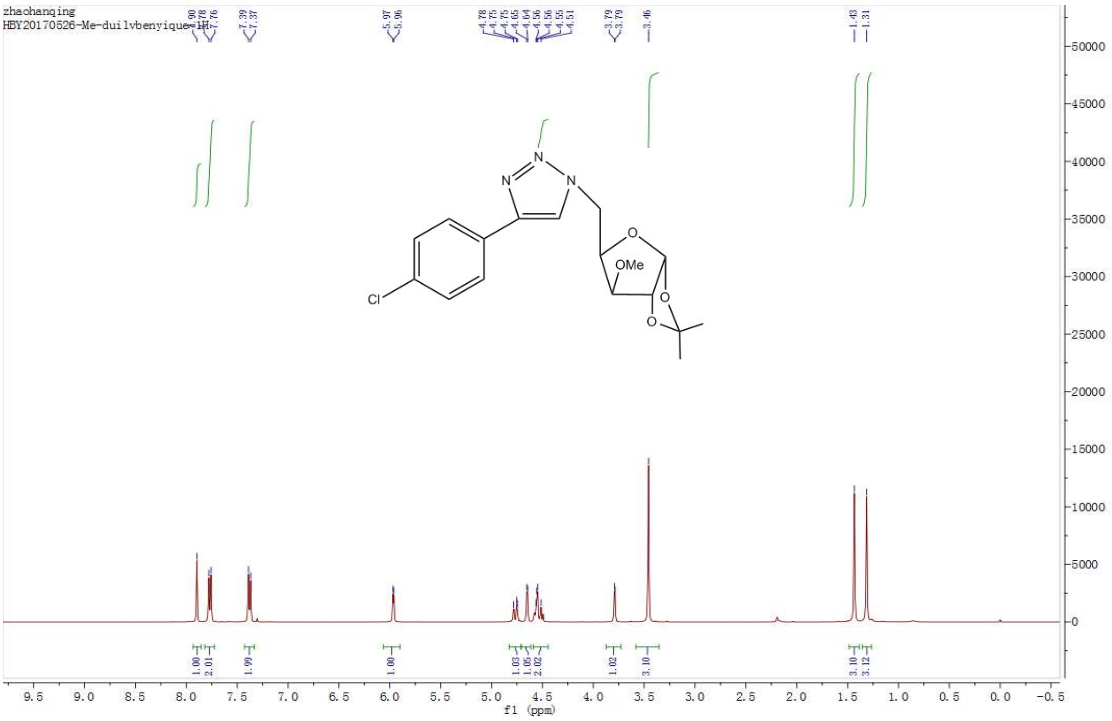

^{13}C NMR spectrum of compound 1-f

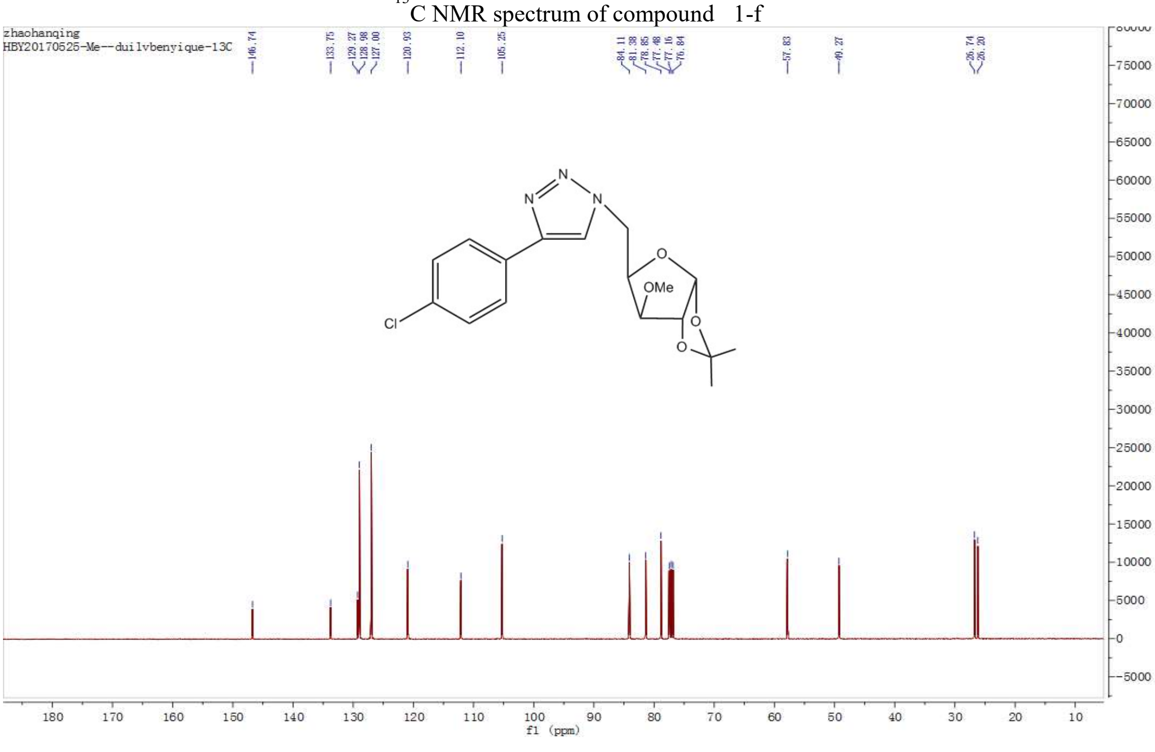

HRMS spectrum of compound 1-f

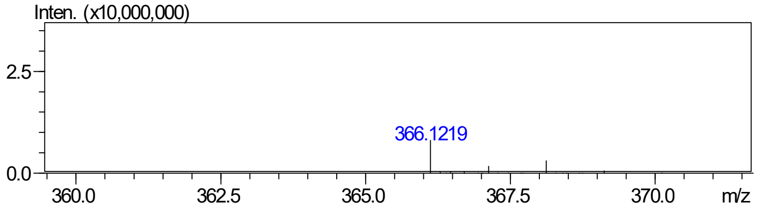

^{1}H NMR spectrum of compound 1-g

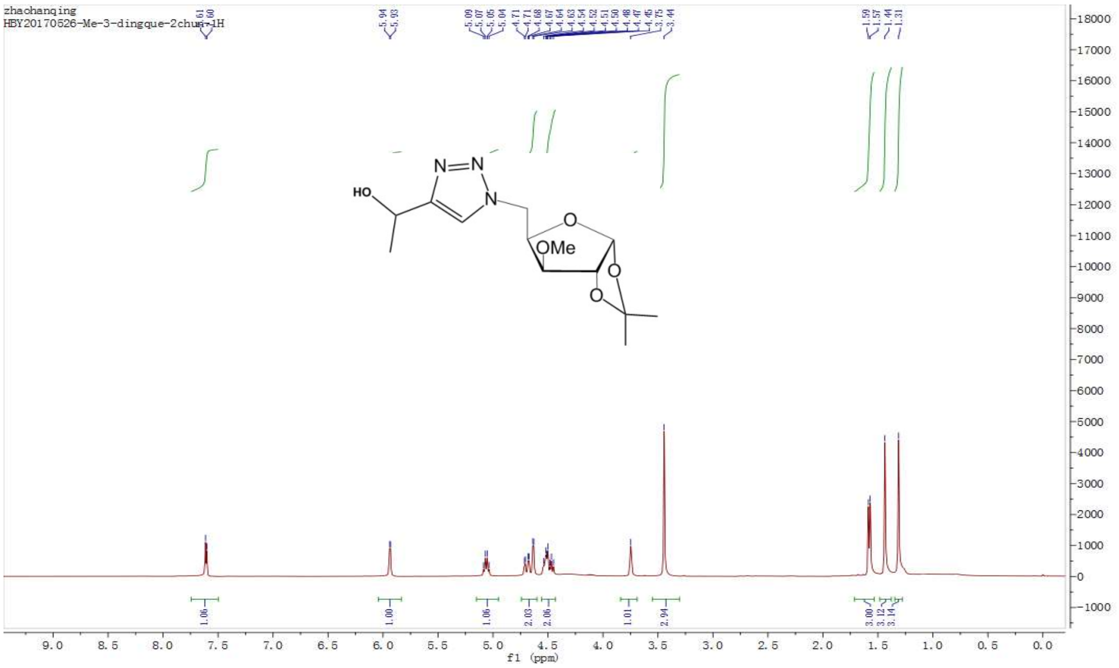

^{13}C NMR spectrum of compound 1-g

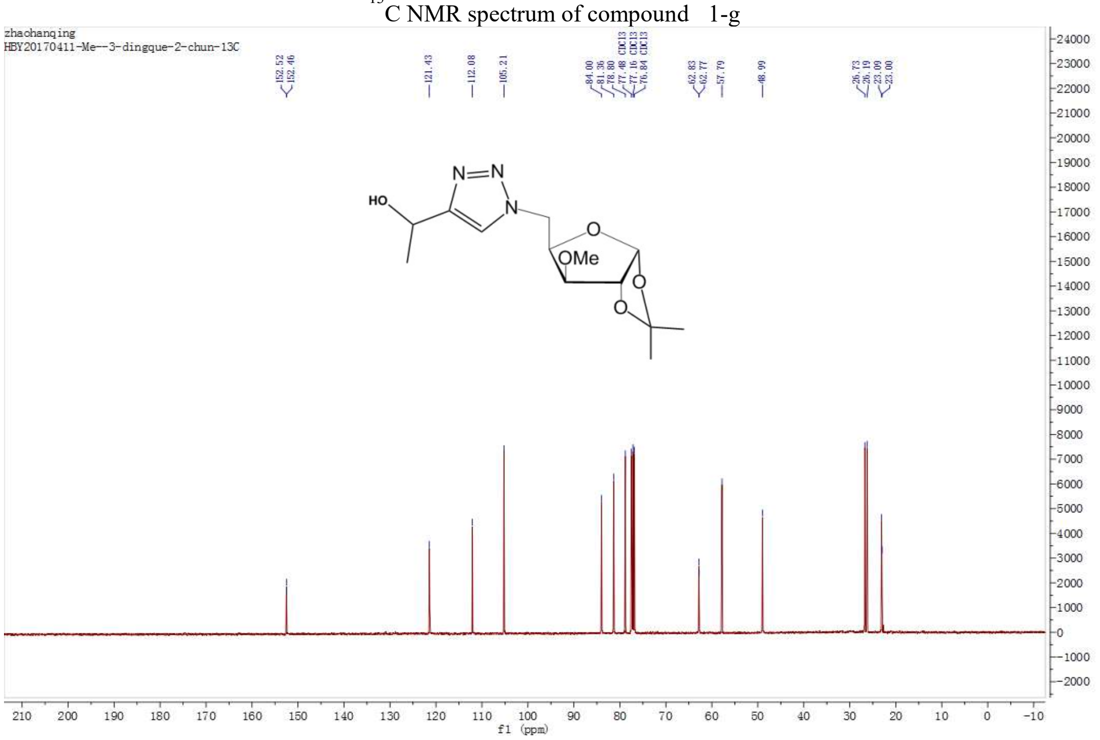

HRMS spectrum of compound 1-g

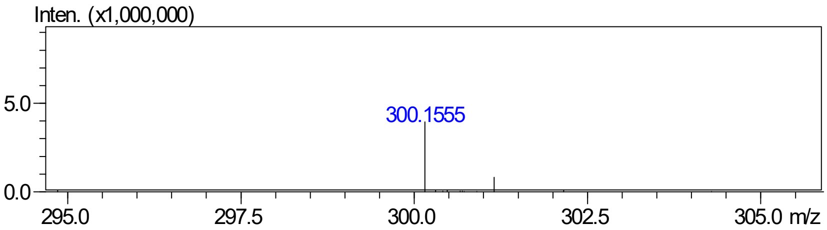

^{1}H NMR spectrum of compound 2-a

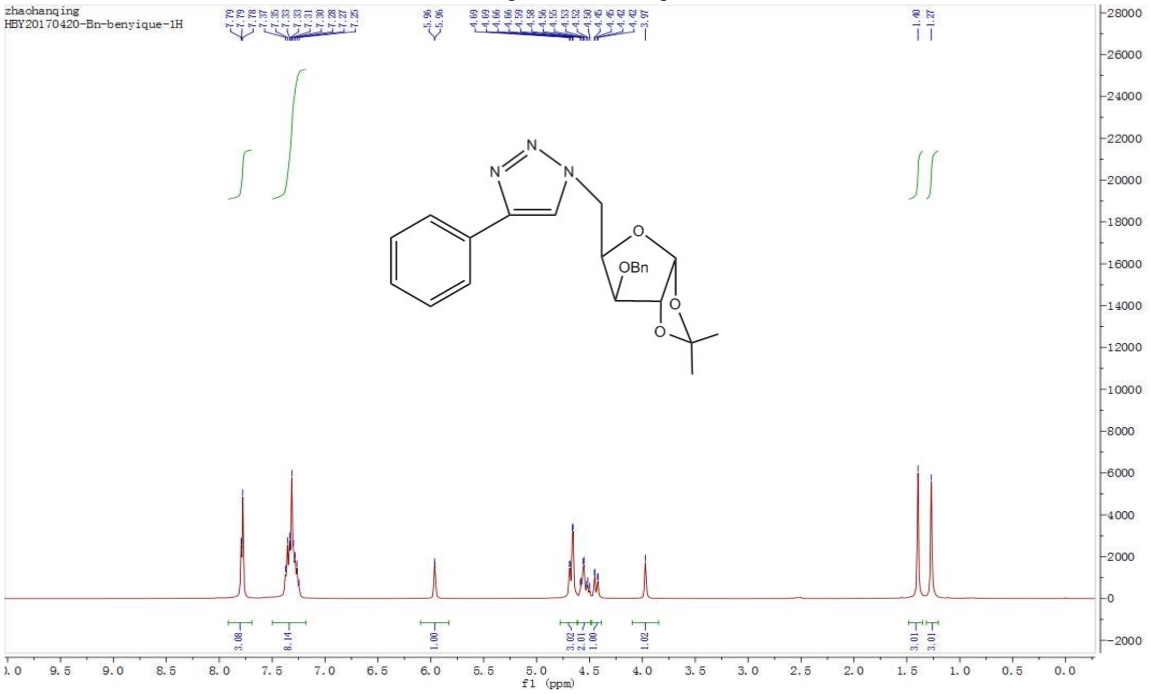

^{13}C NMR spectrum of compound 2-a

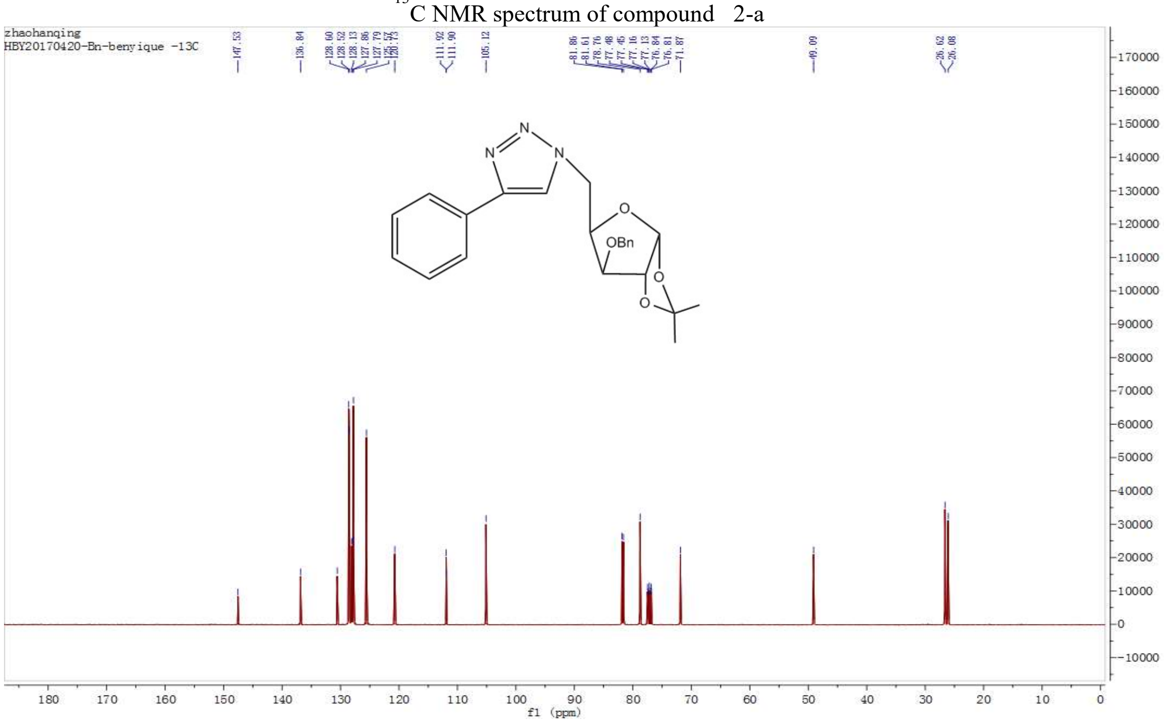

HRMS spectrum of compound 2-a

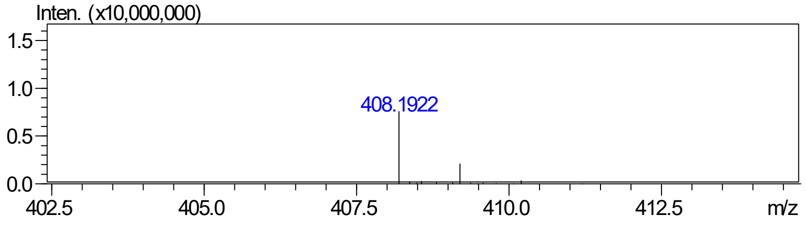

^{1}H NMR spectrum of compound 2-b

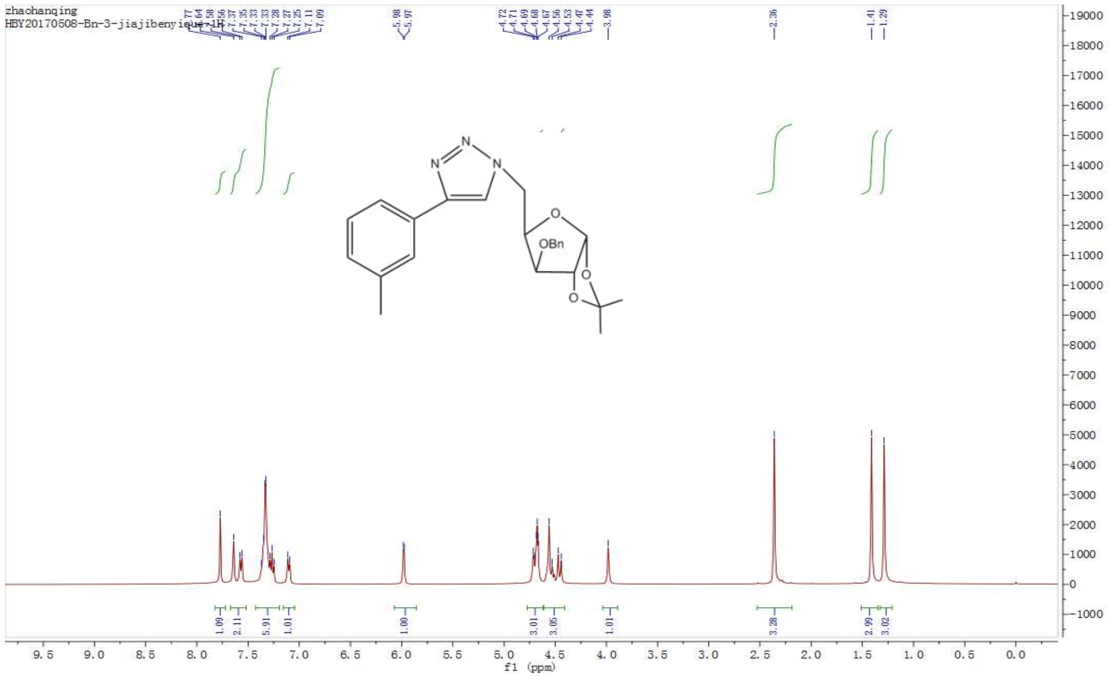

^{13}C NMR spectrum of compound 2-b

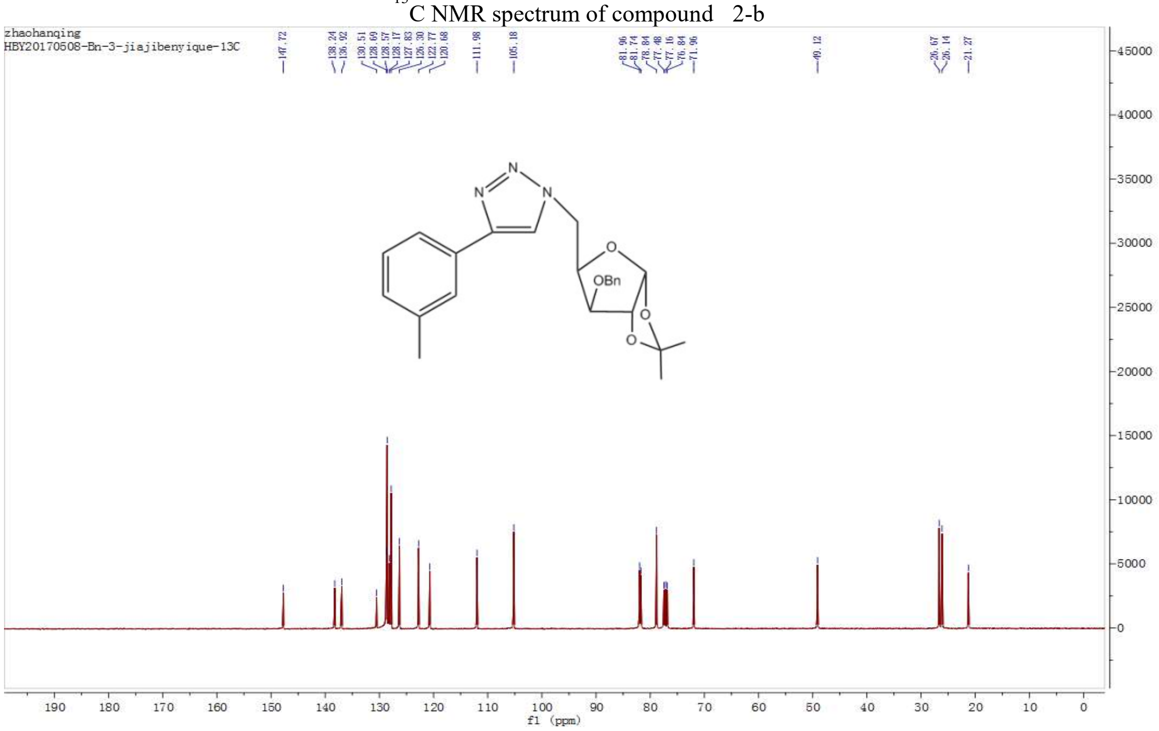

HRMS spectrum of compound 2-b

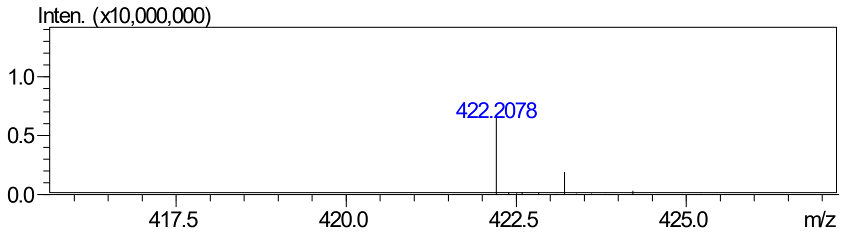

^{1}H NMR spectrum of compound 2-c

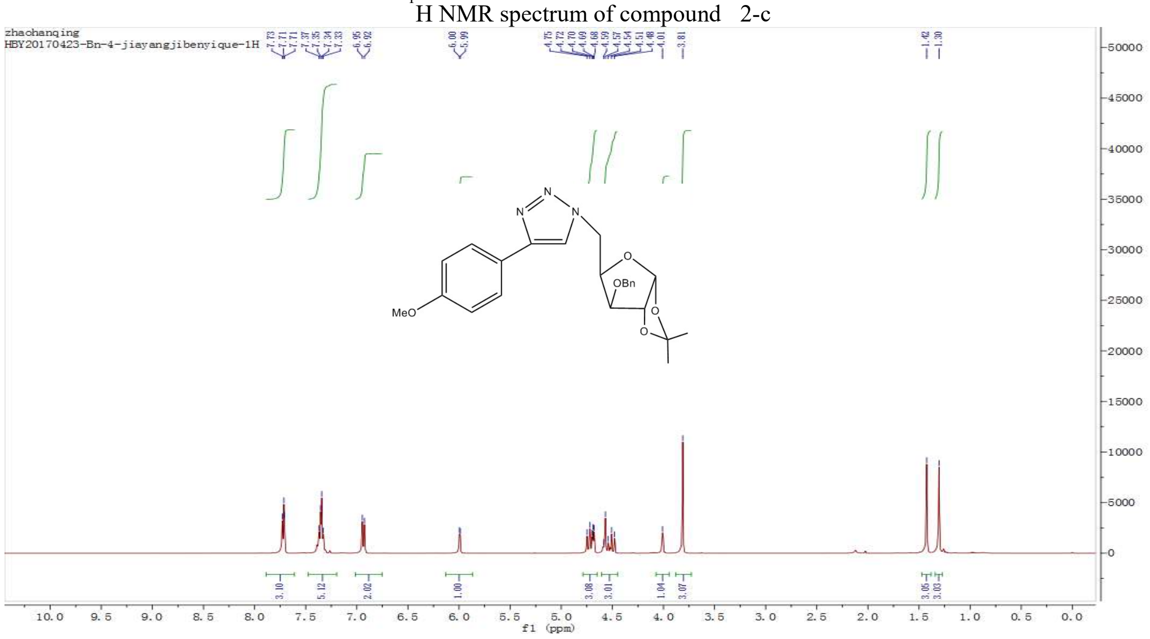

^{13}C NMR spectrum of compound 2-c

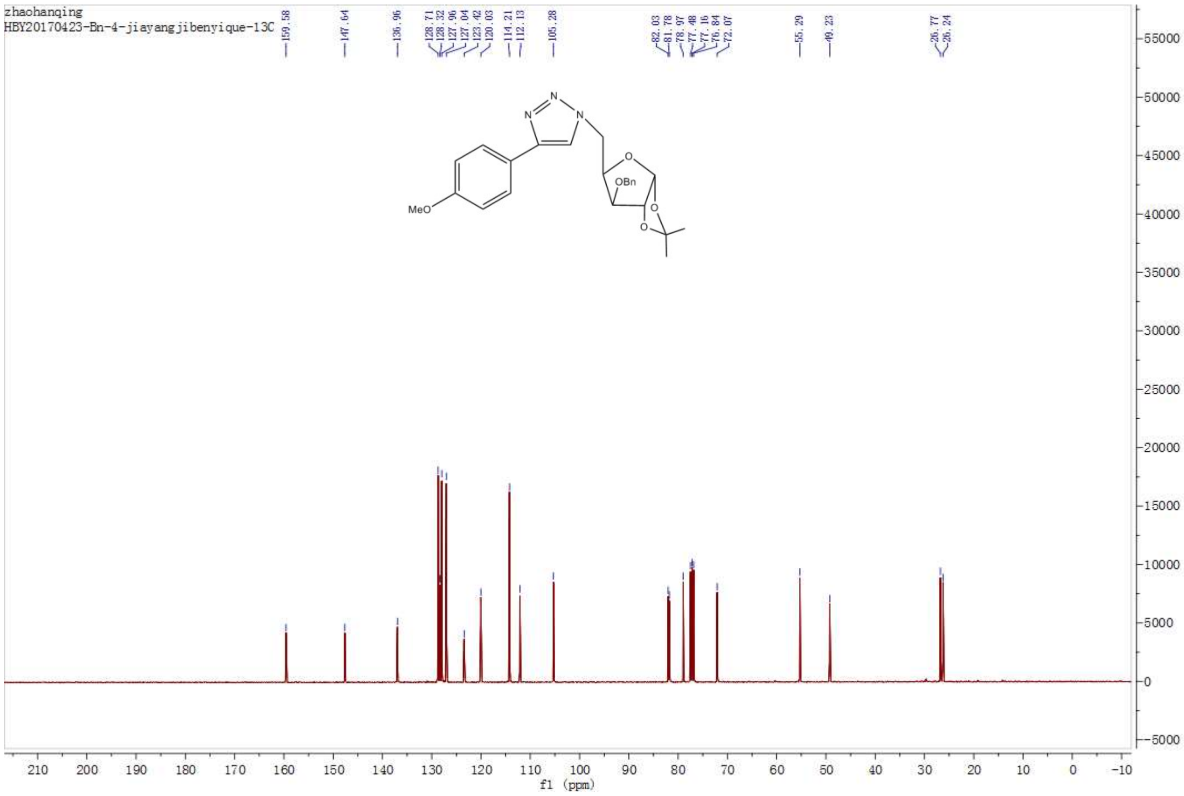

HRMS spectrum of compound 2-c

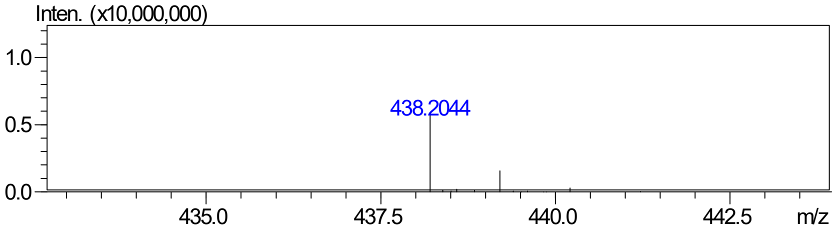

^{1}H NMR spectrum of compound 2-d

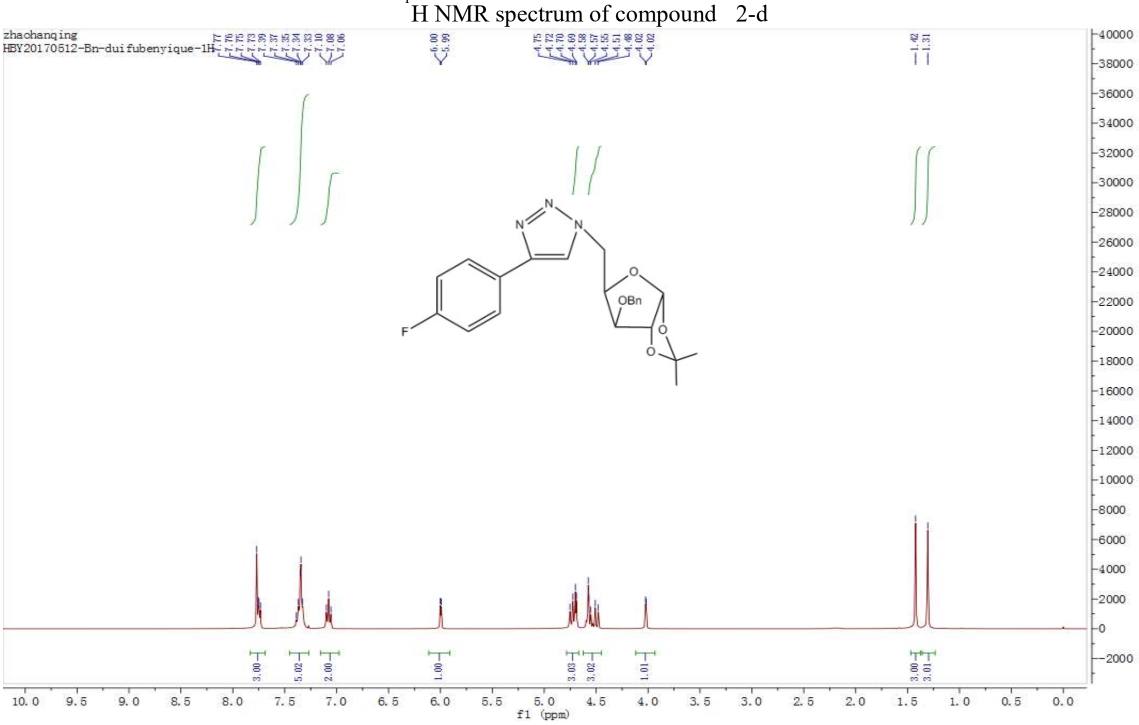

^{13}C NMR spectrum of compound 2-d

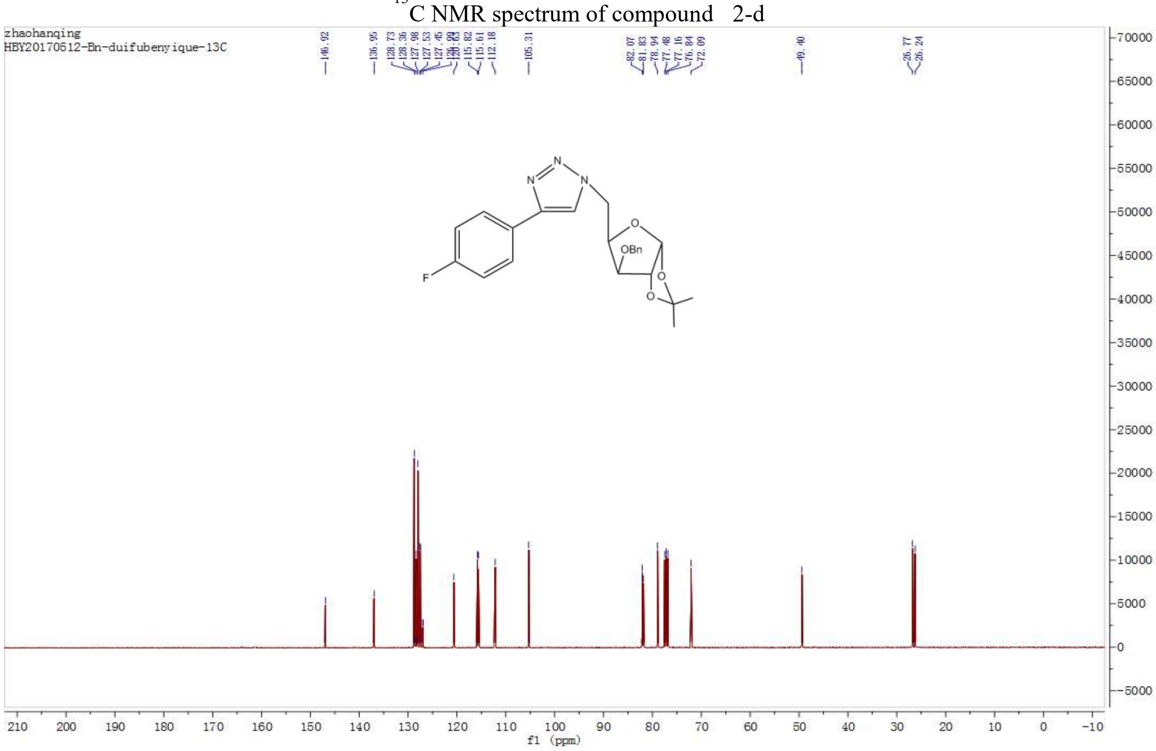

HRMS spectrum of compound 2-d

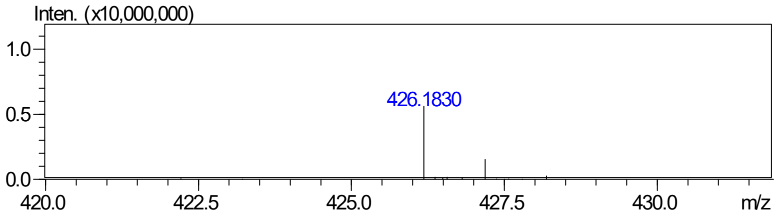

^{1}H NMR spectrum of compound 2-e

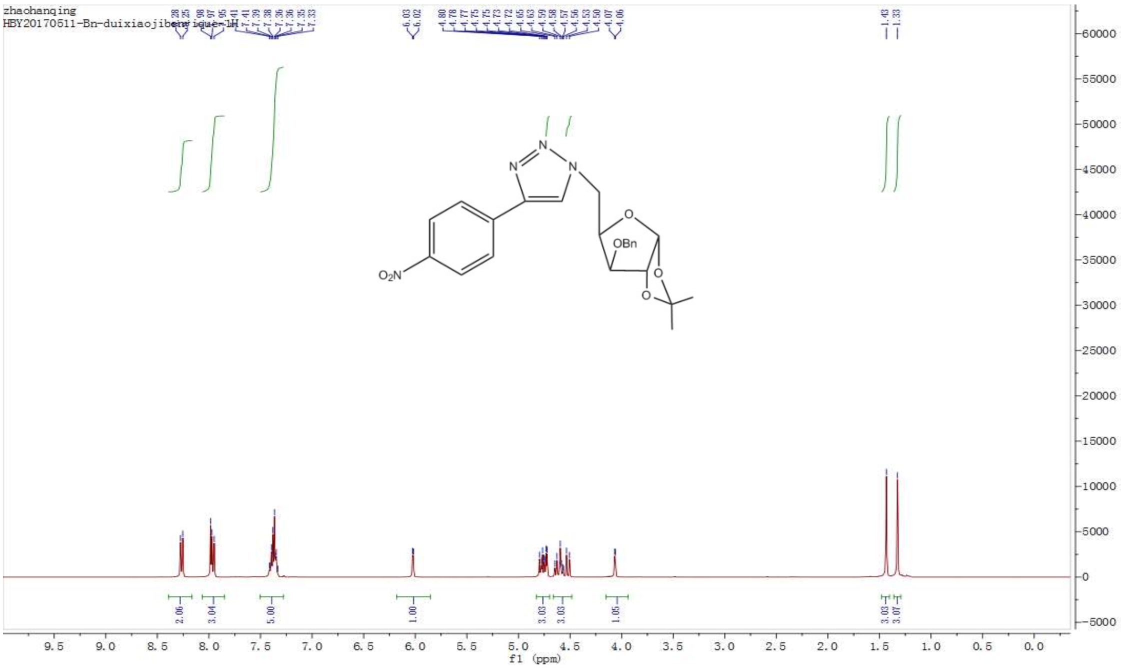

^{13}C NMR spectrum of compound 2-e

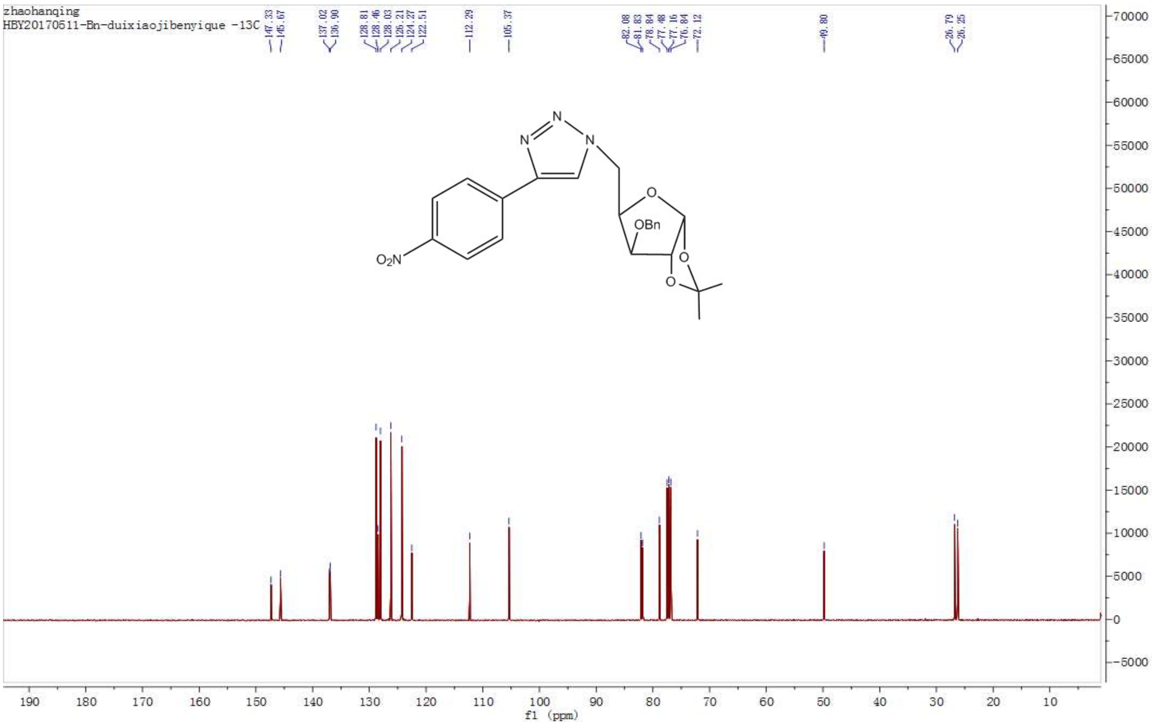

HRMS spectrum of compound 2-e

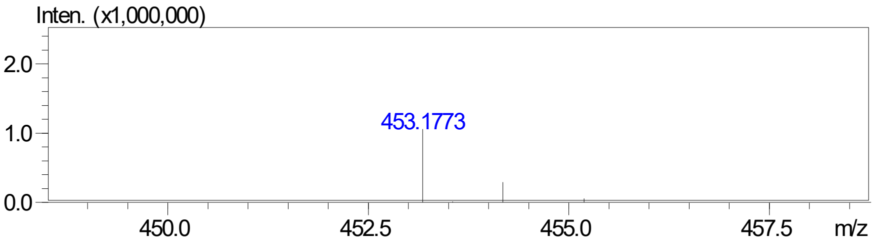

^{1}H NMR spectrum of compound 2-f

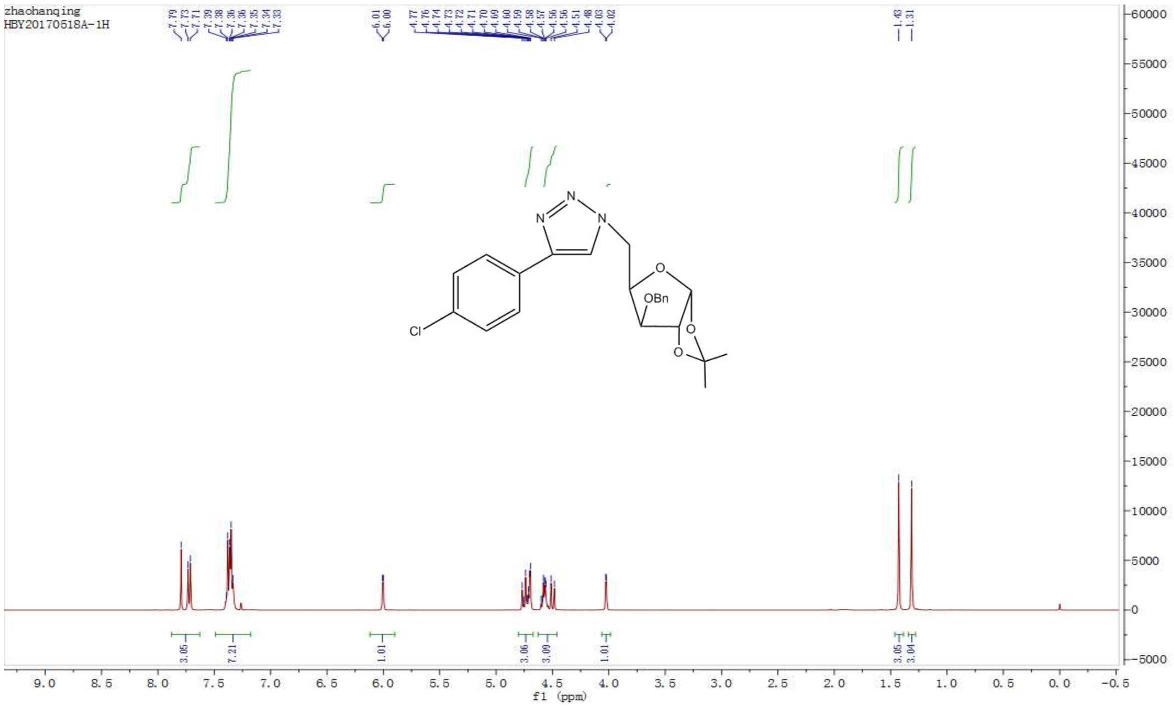

^{13}C NMR spectrum of compound 2-f

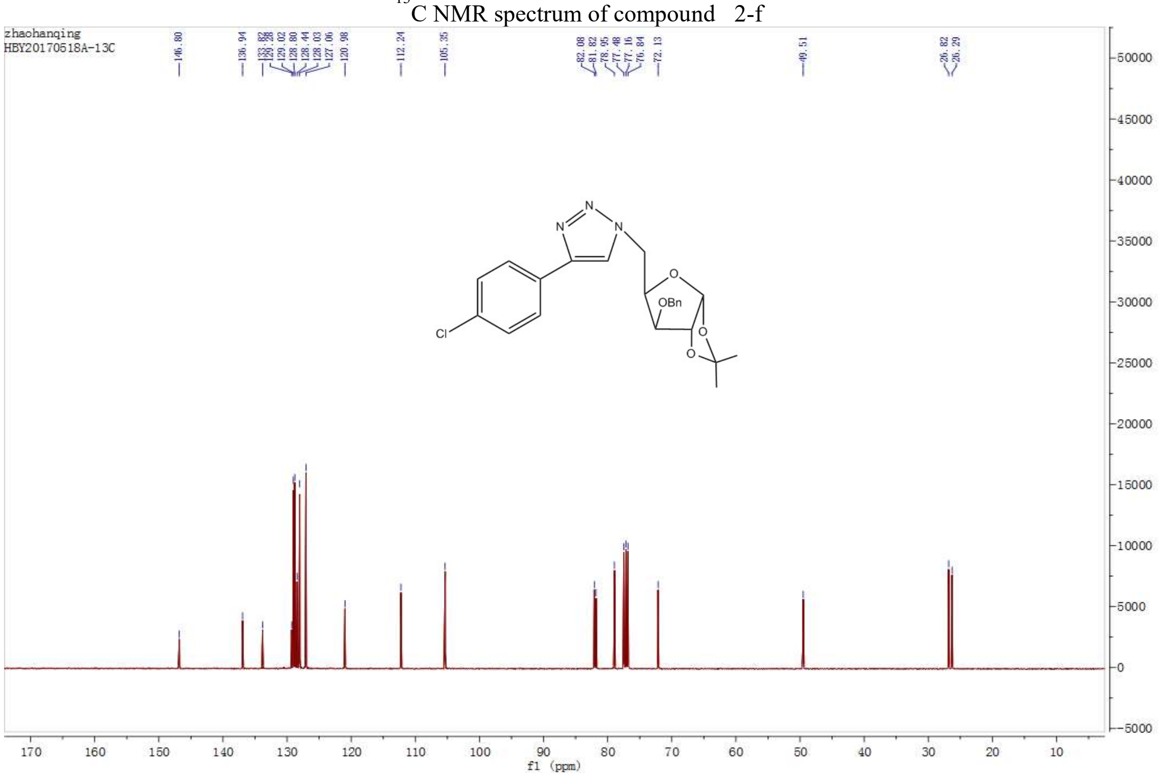

HRMS spectrum of compound 2-f

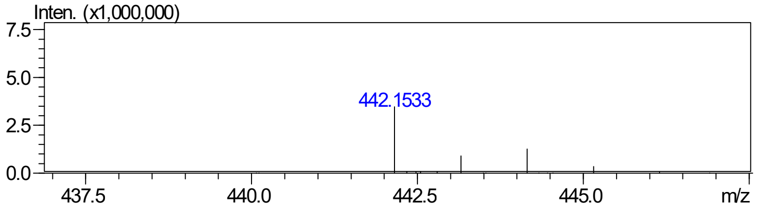

^{1}H NMR spectrum of compound 2-g

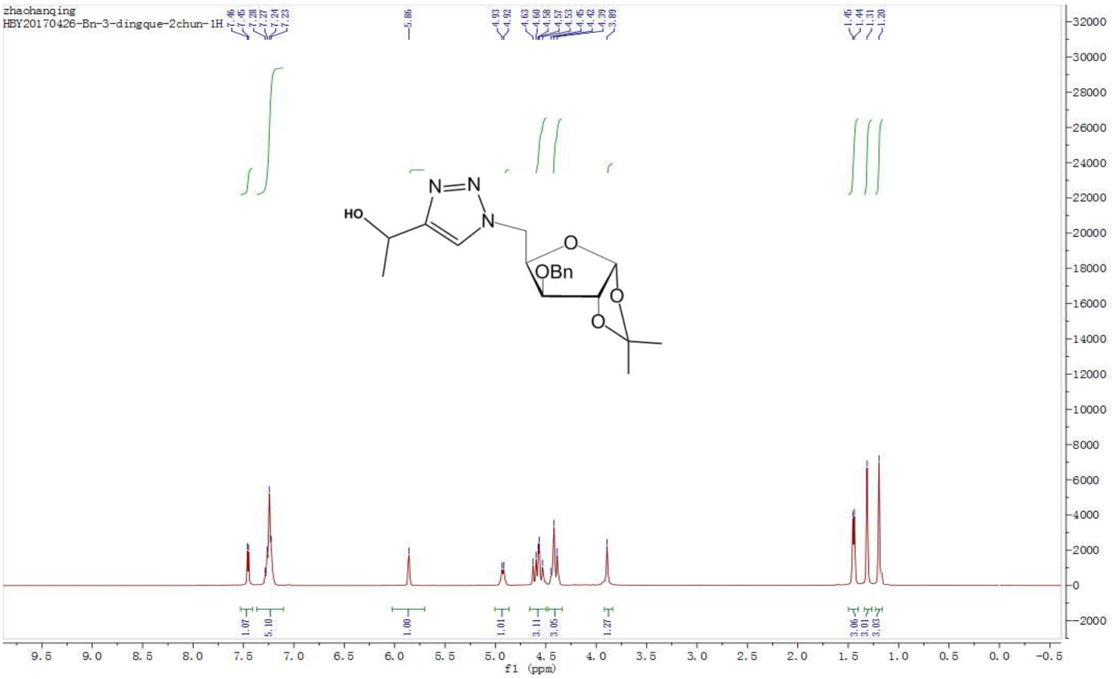

^{13}C NMR spectrum of compound 2-g

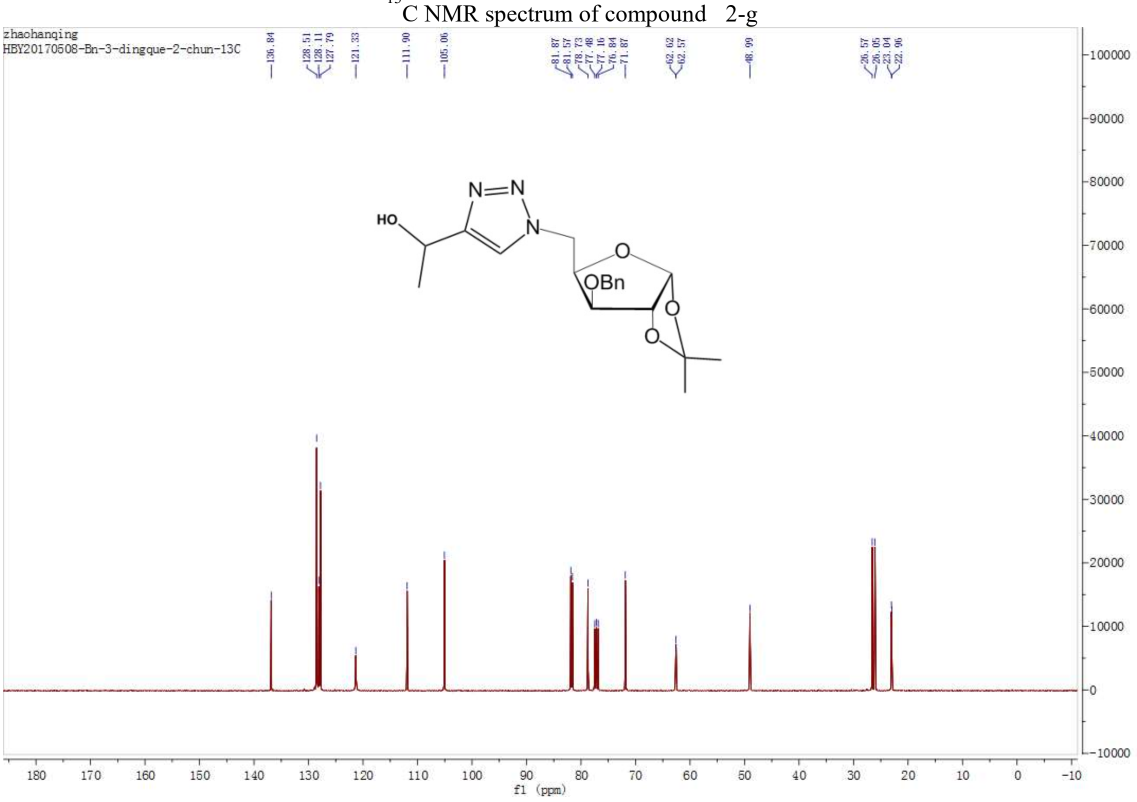

HRMS spectrum of compound 2-g

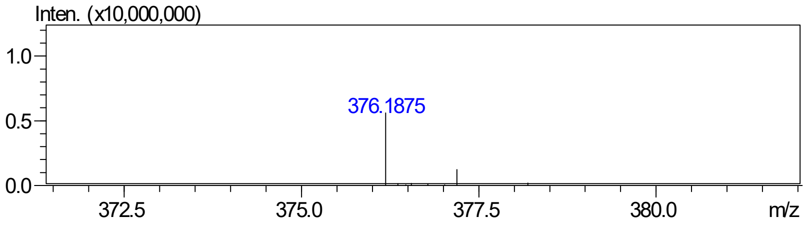

^{1}H NMR spectrum of compound 3-a

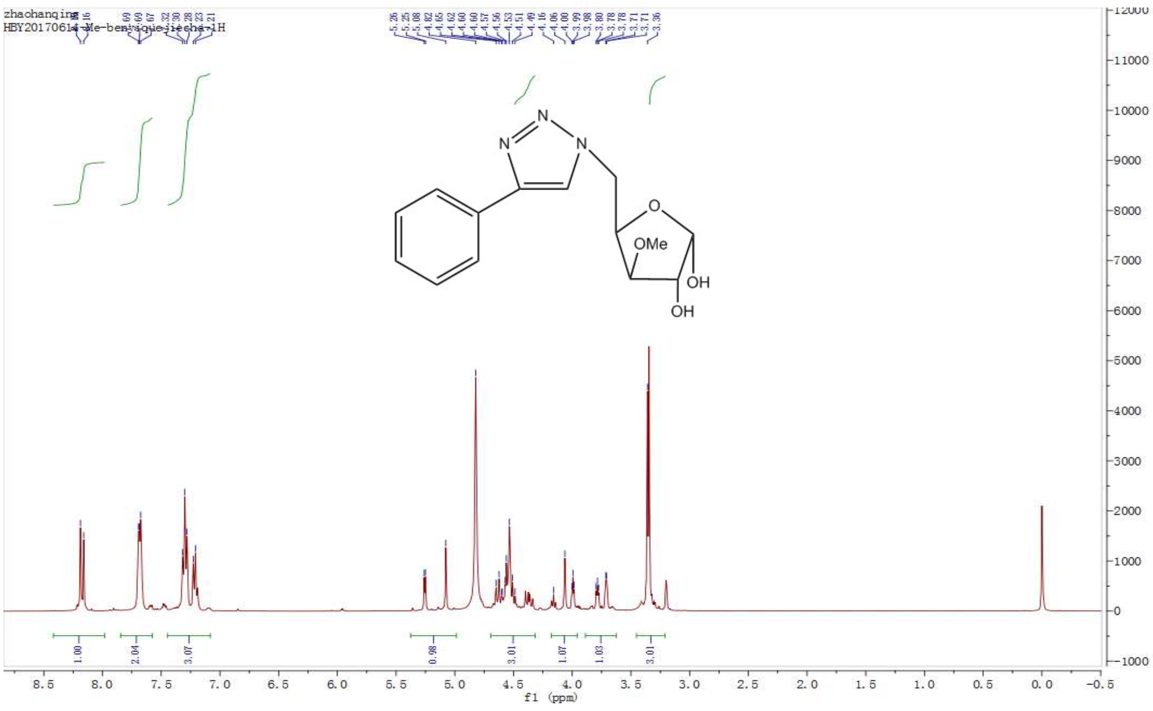

^{13}C NMR spectrum of compound 3-a

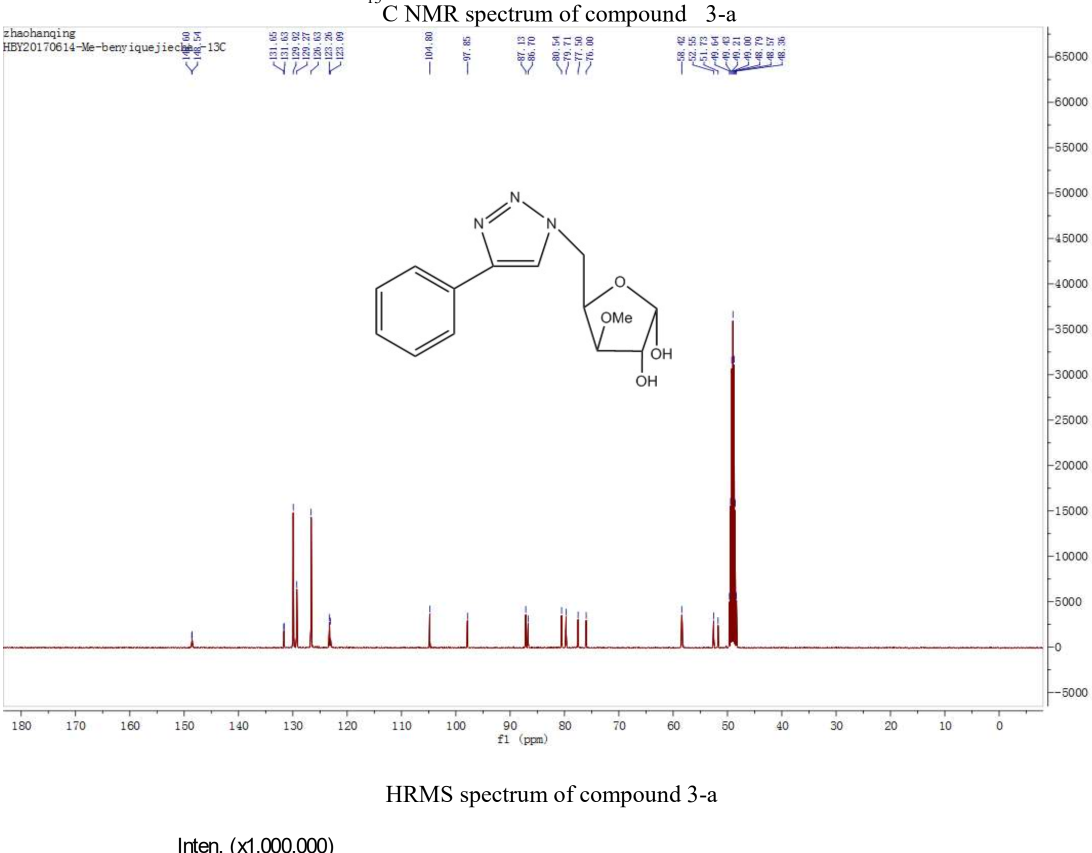

HRMS spectrum of compound 3-a

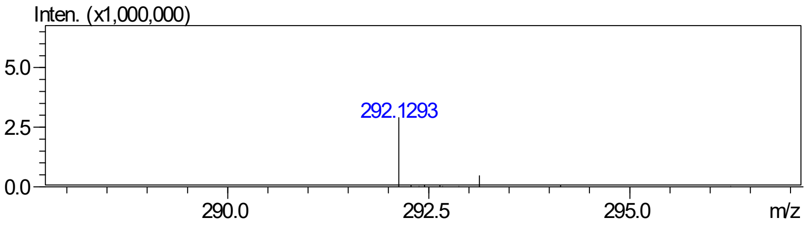

^{1}H NMR spectrum of compound 3-b

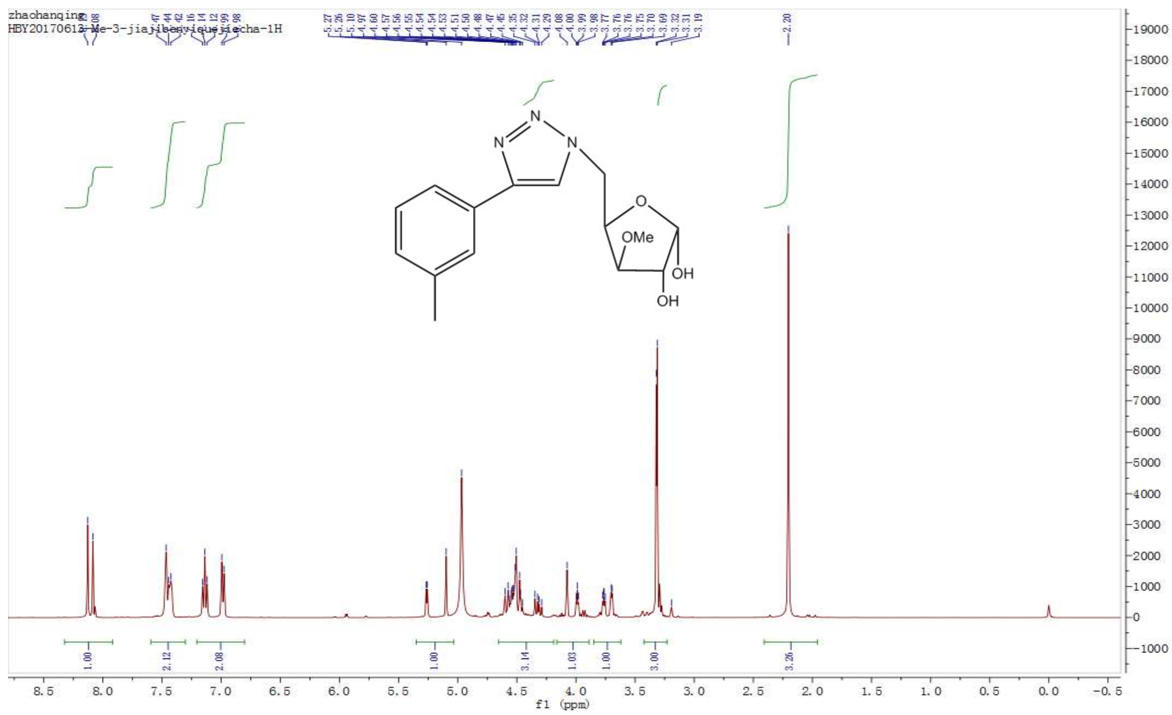

^{13}C NMR spectrum of compound 3-b

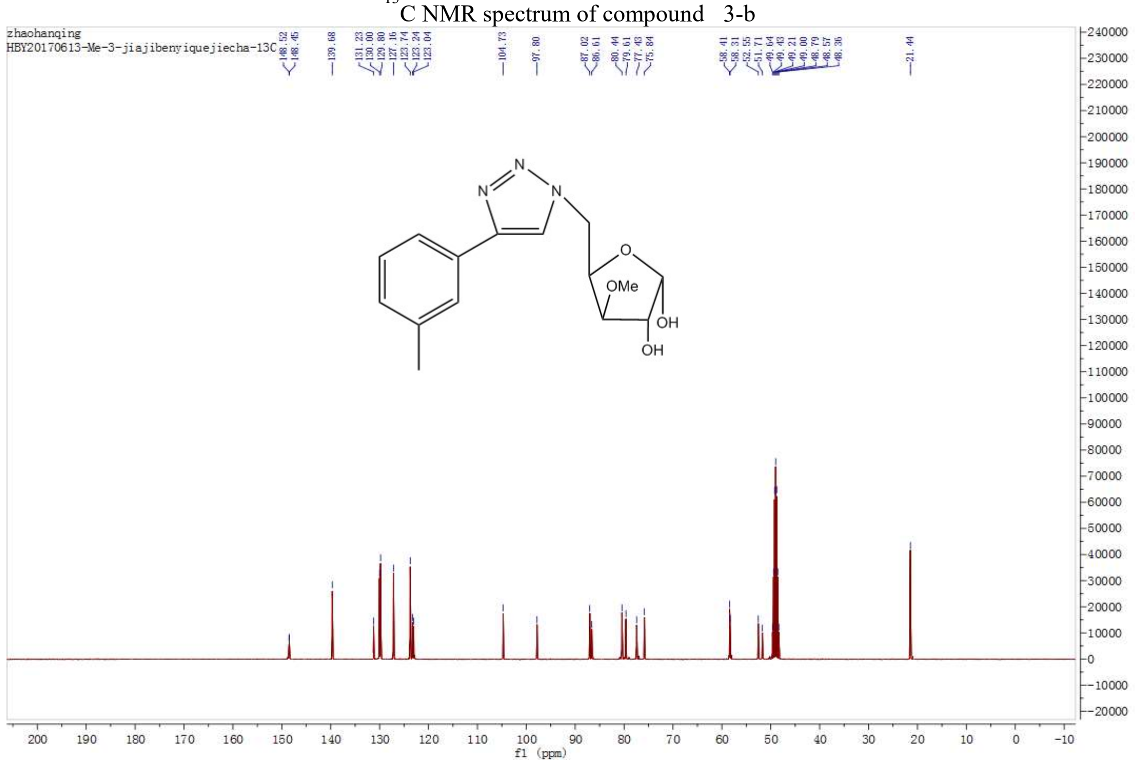

HRMS spectrum of compound 3-b

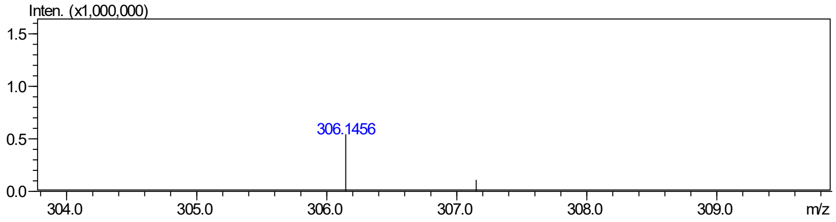

^{1}H NMR spectrum of compound 3-c

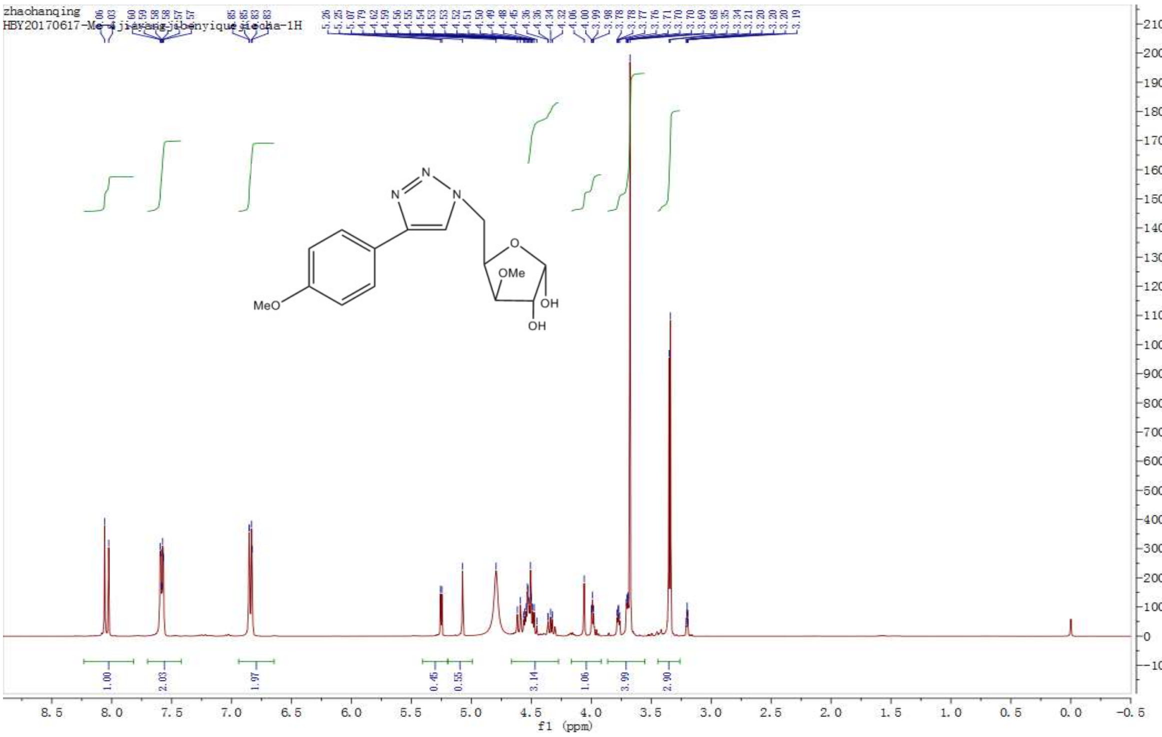

^{13}C NMR spectrum of compound 3-c

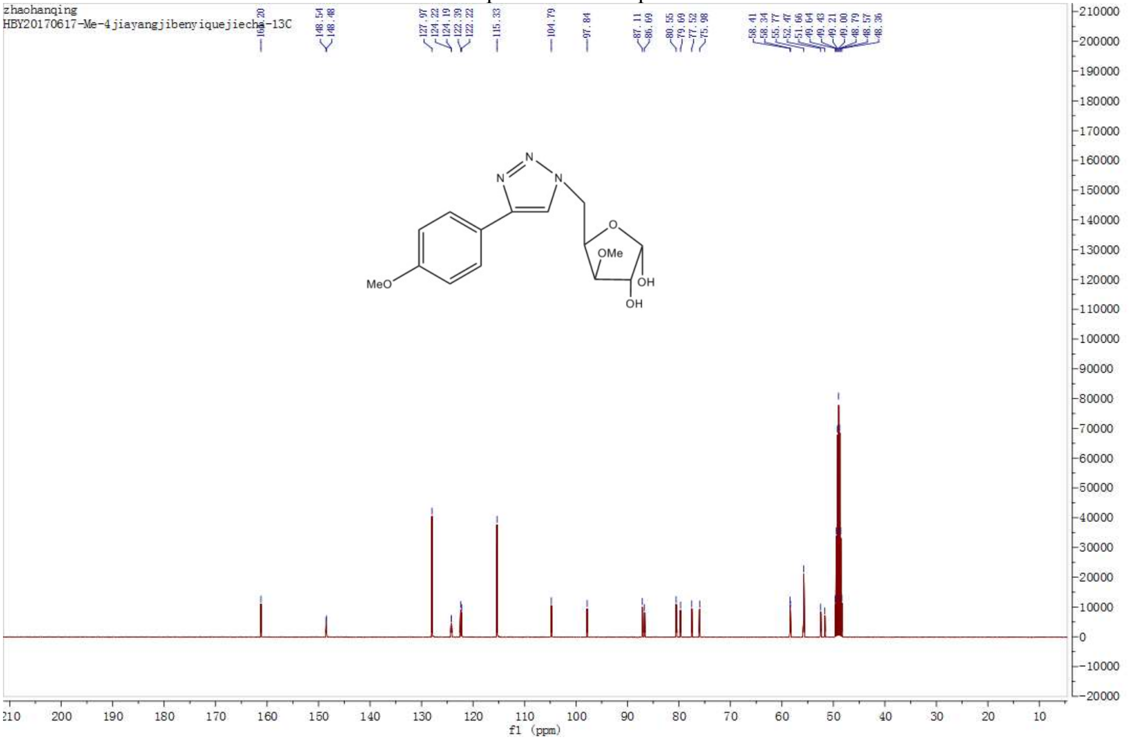

HRMS spectrum of compound 3-c

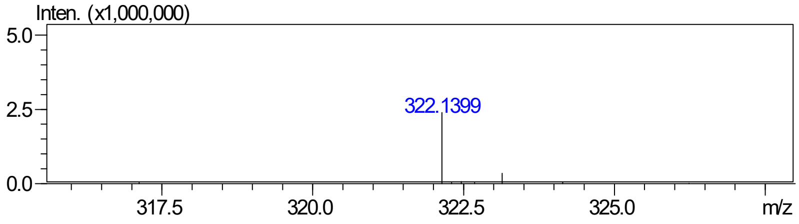

^{1}H NMR spectrum of compound 3-d

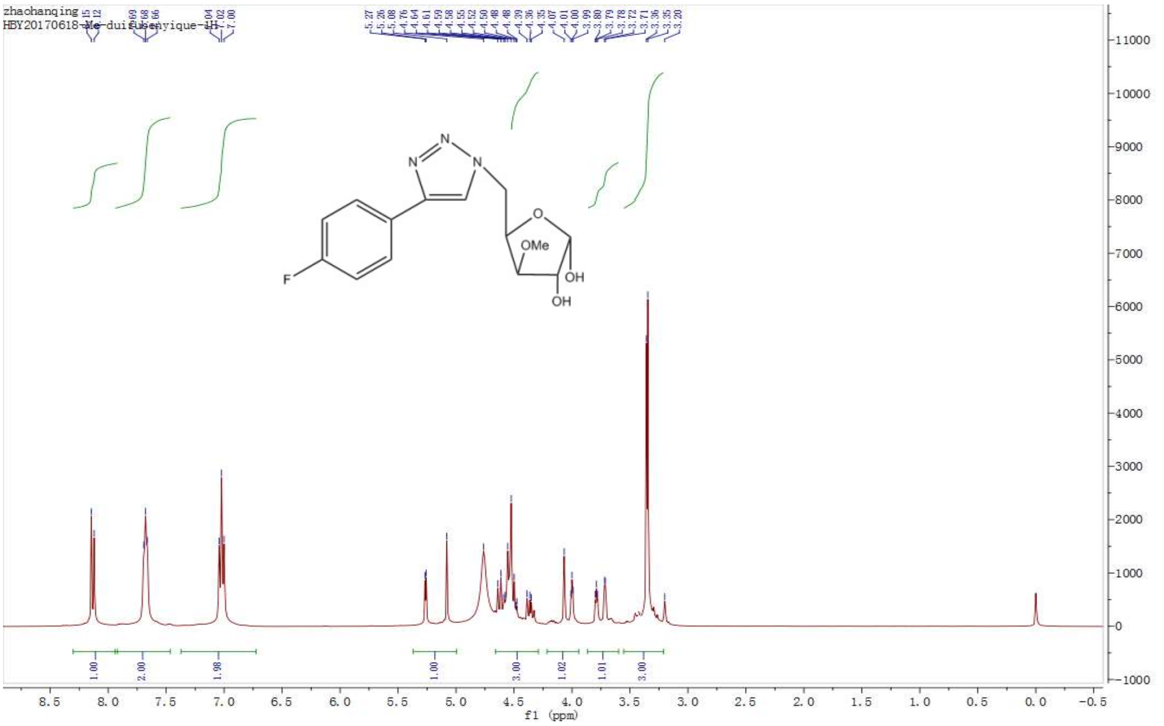

^{13}C NMR spectrum of compound 3-d

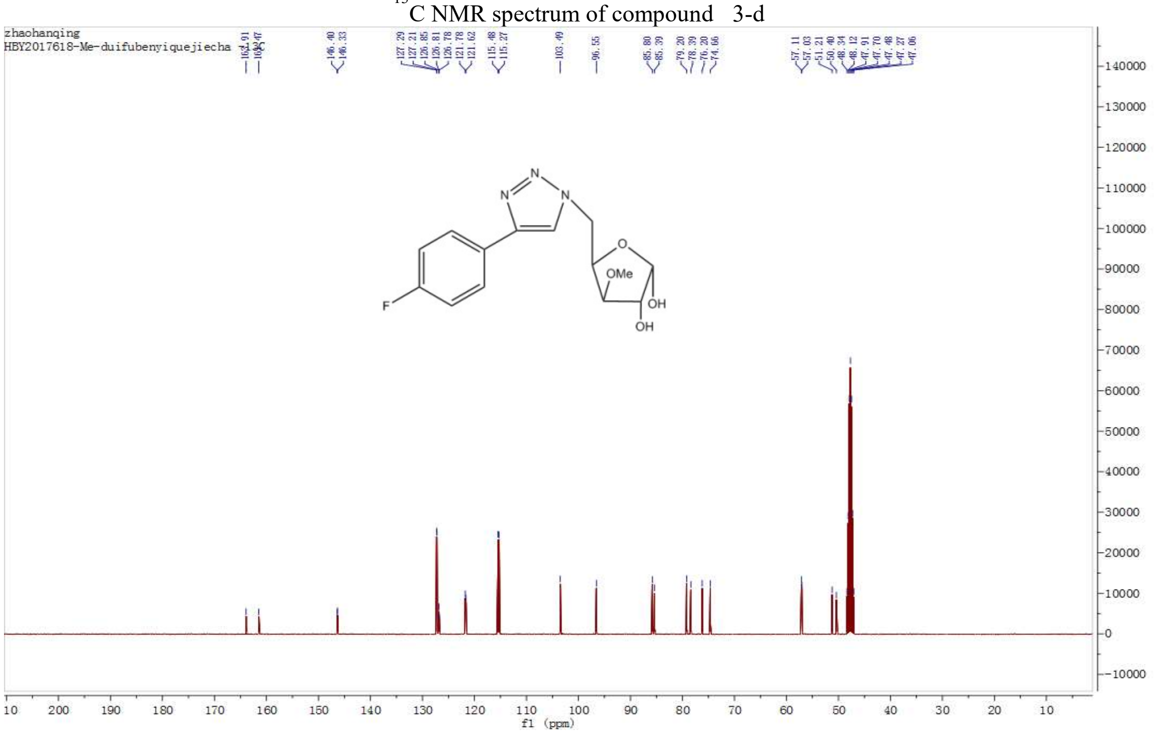

HRMS spectrum of compound 3-d

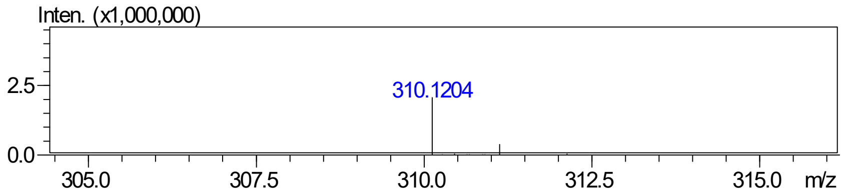

^{1}H NMR spectrum of compound 3-e

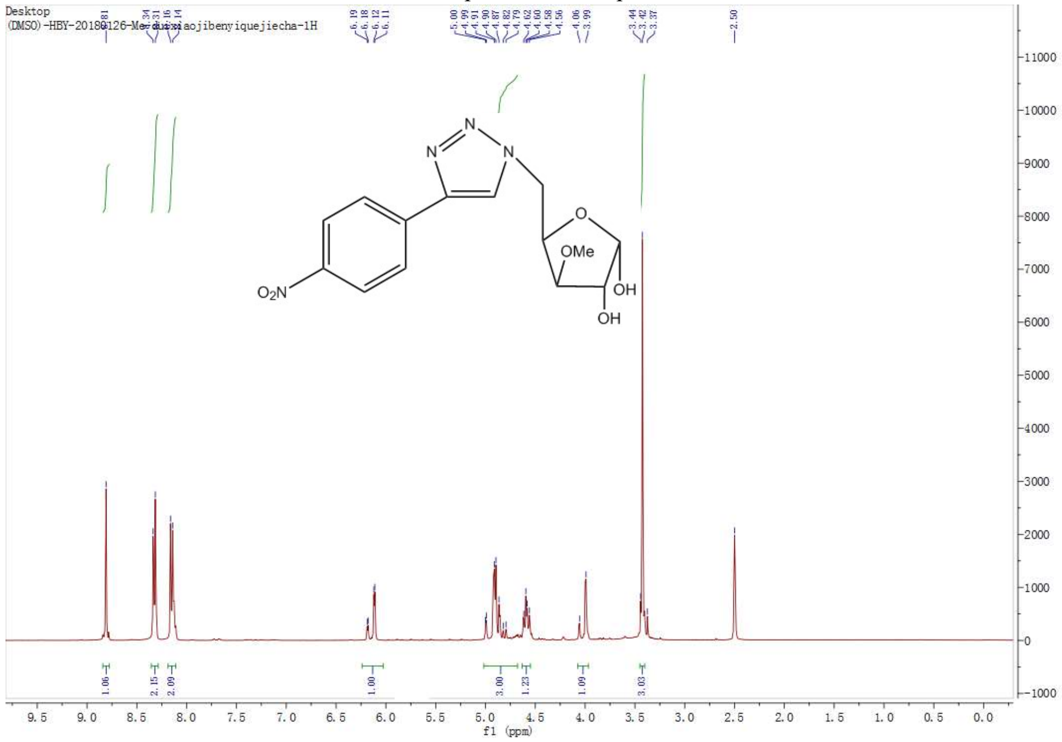

^{13}C NMR spectrum of compound 3-e

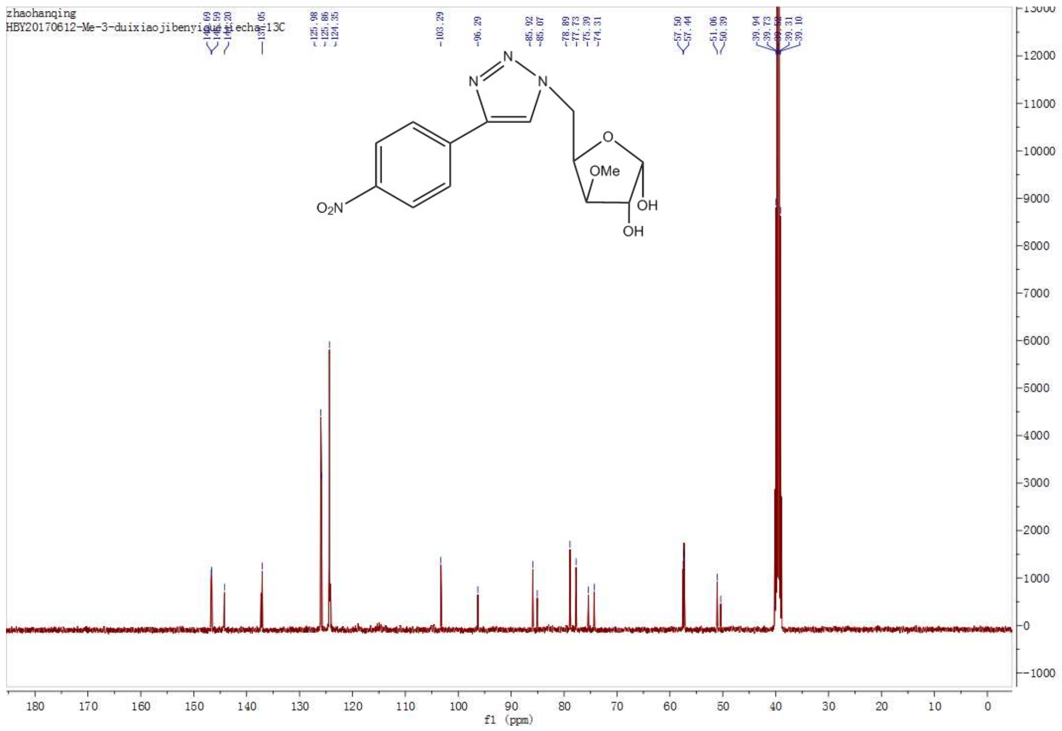

HRMS spectrum of compound 3-e

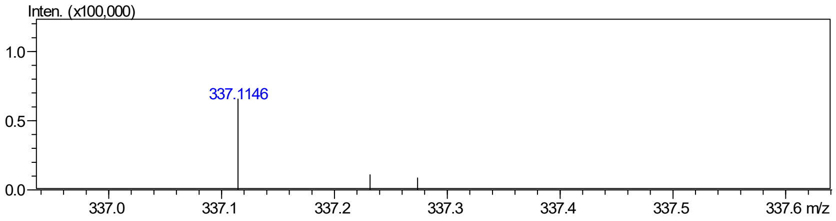

^{1}H NMR spectrum of compound 3-f

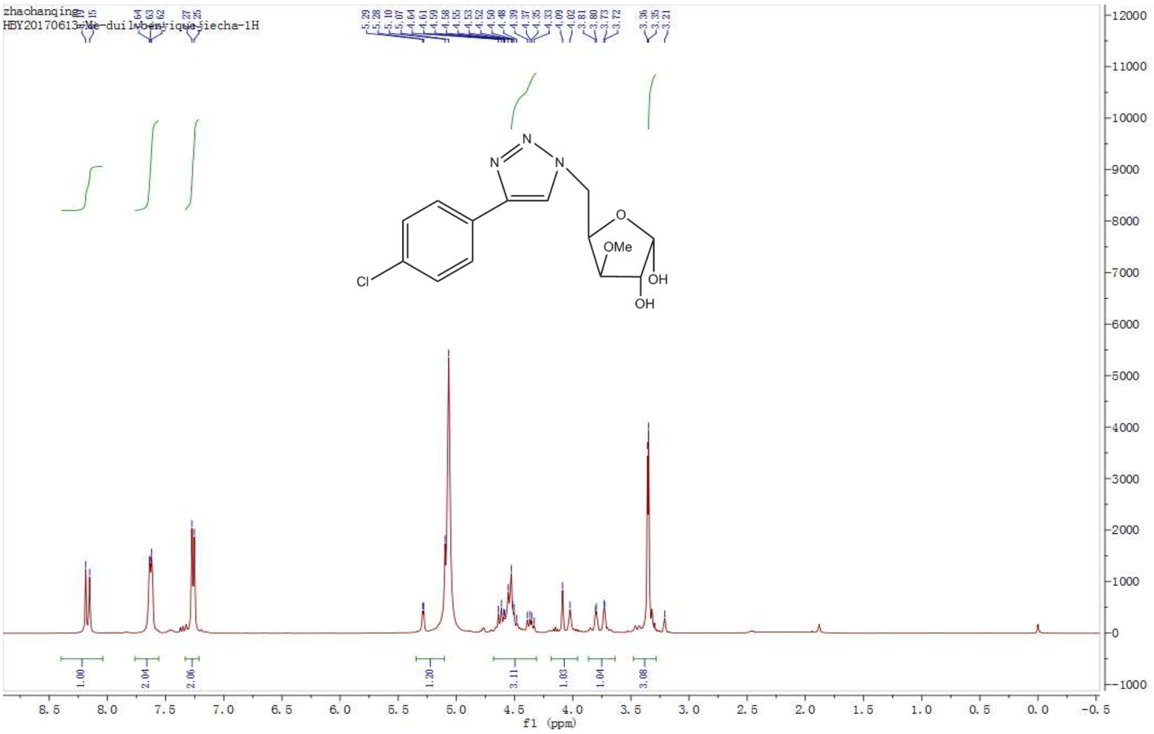

^{13}C NMR spectrum of compound 3-f

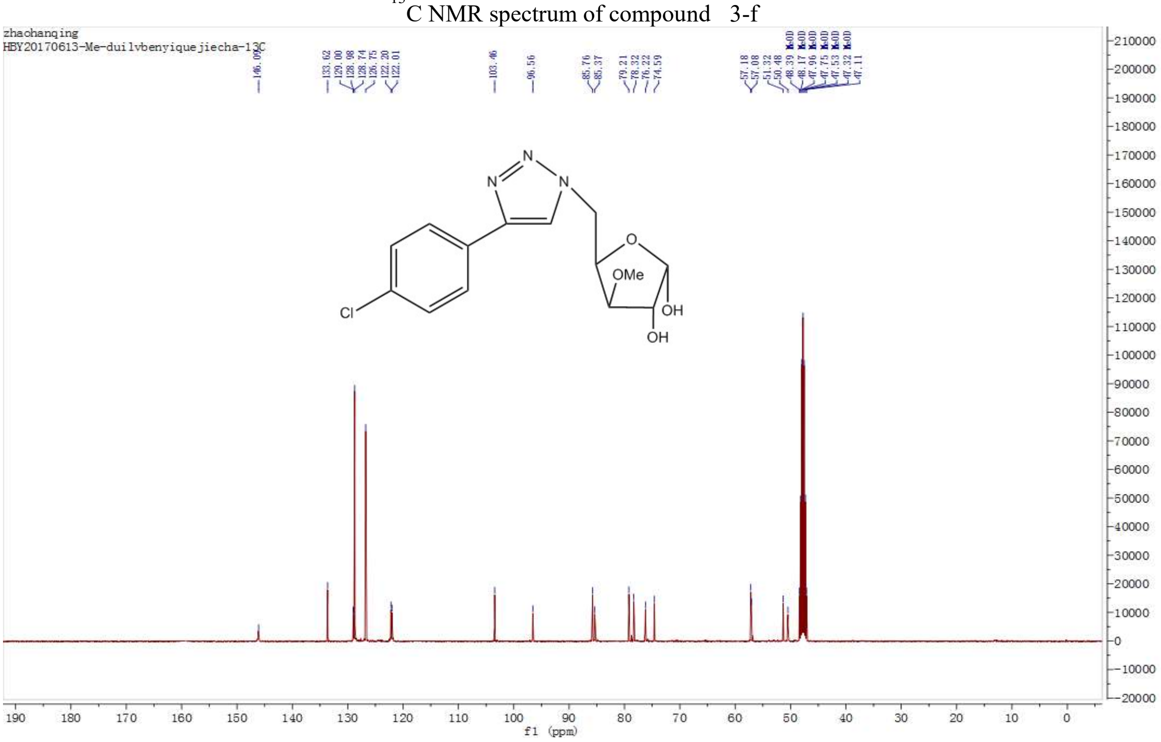

HRMS spectrum of compound 3-f

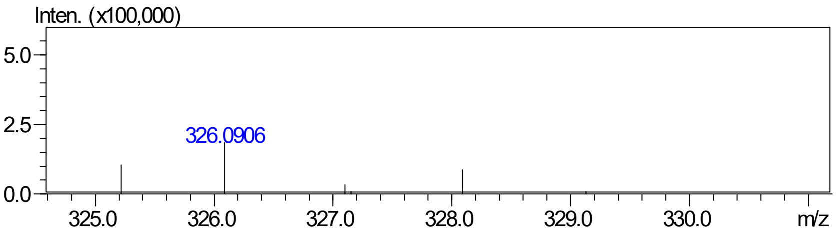

^{1}H NMR spectrum of compound 3-g

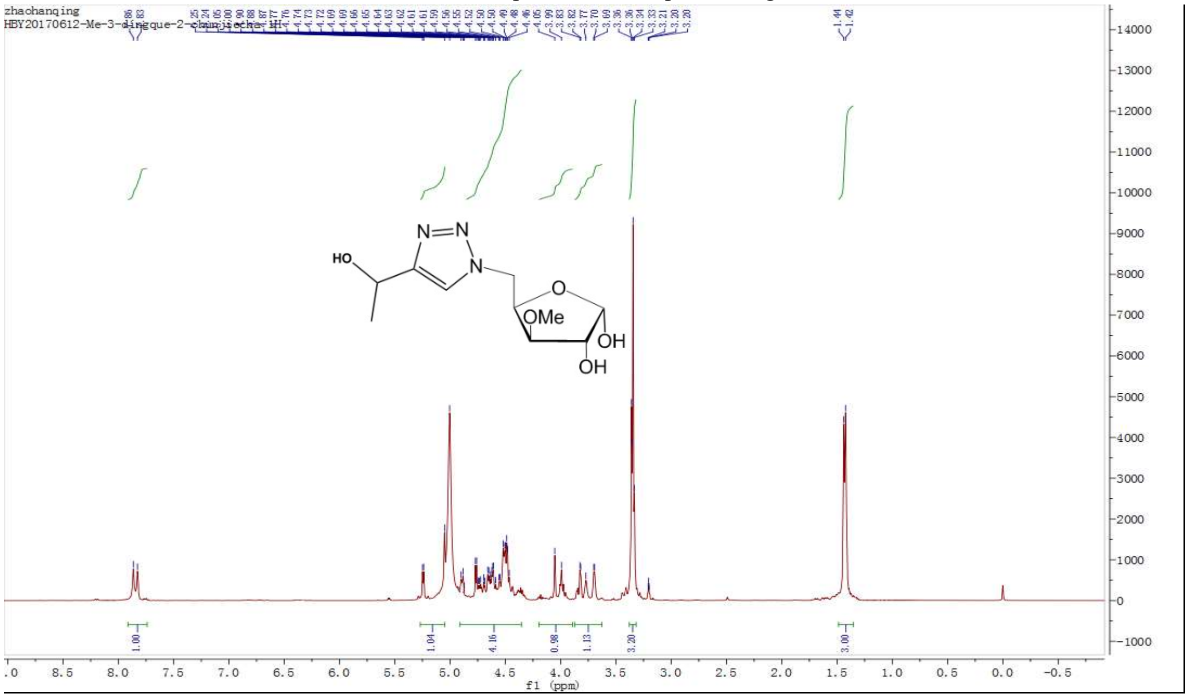

^{13}C NMR spectrum of compound 3-g

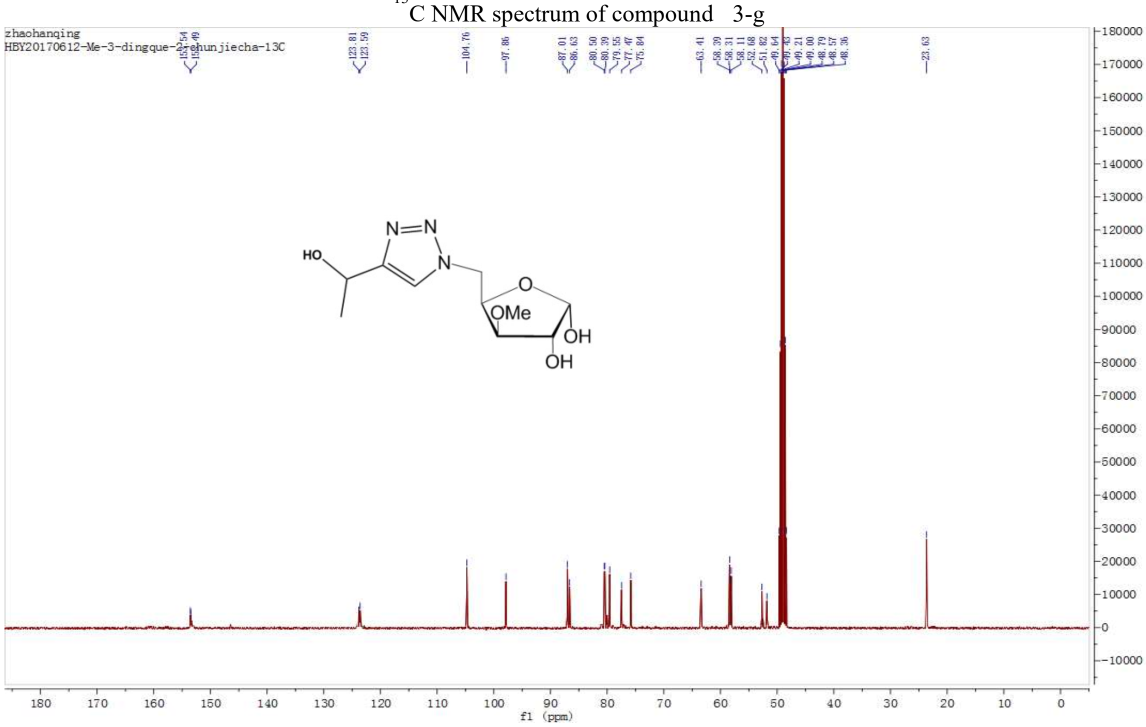

HRMS spectrum of compound 3-g

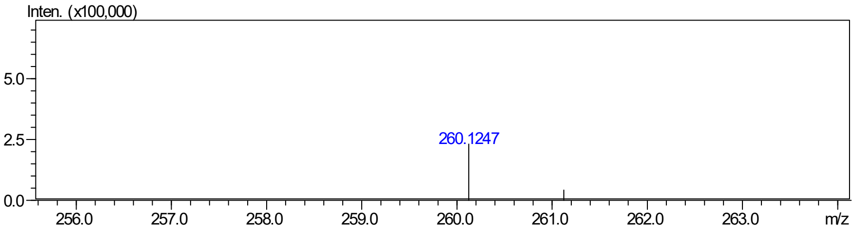

^{1}H NMR spectrum of compound 4-a

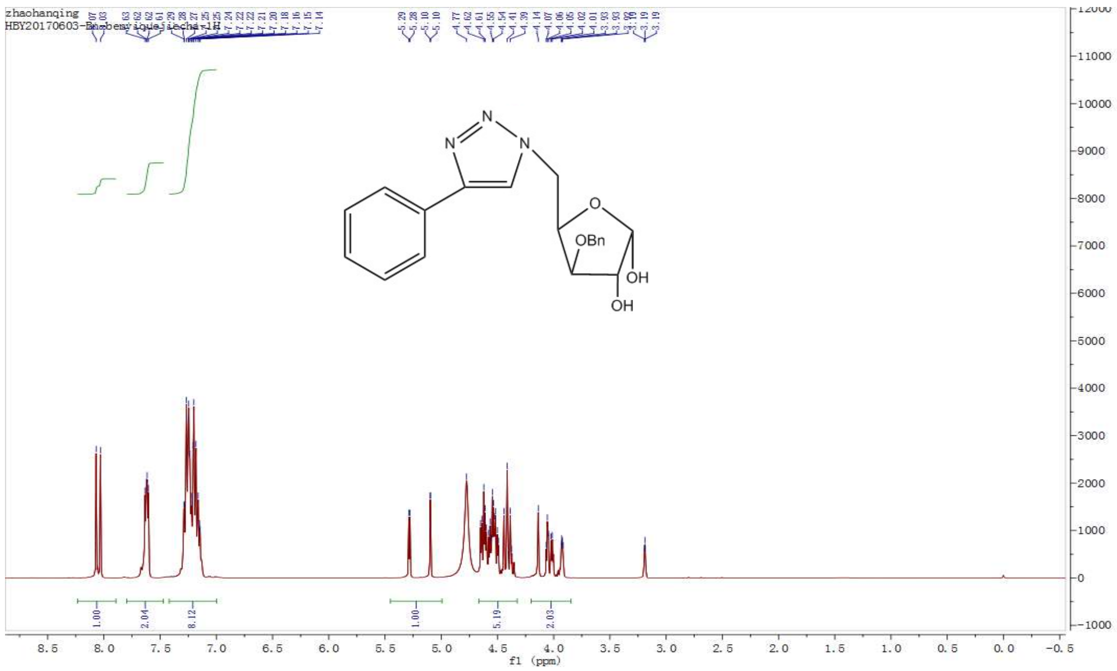

^{13}C NMR spectrum of compound 4-a

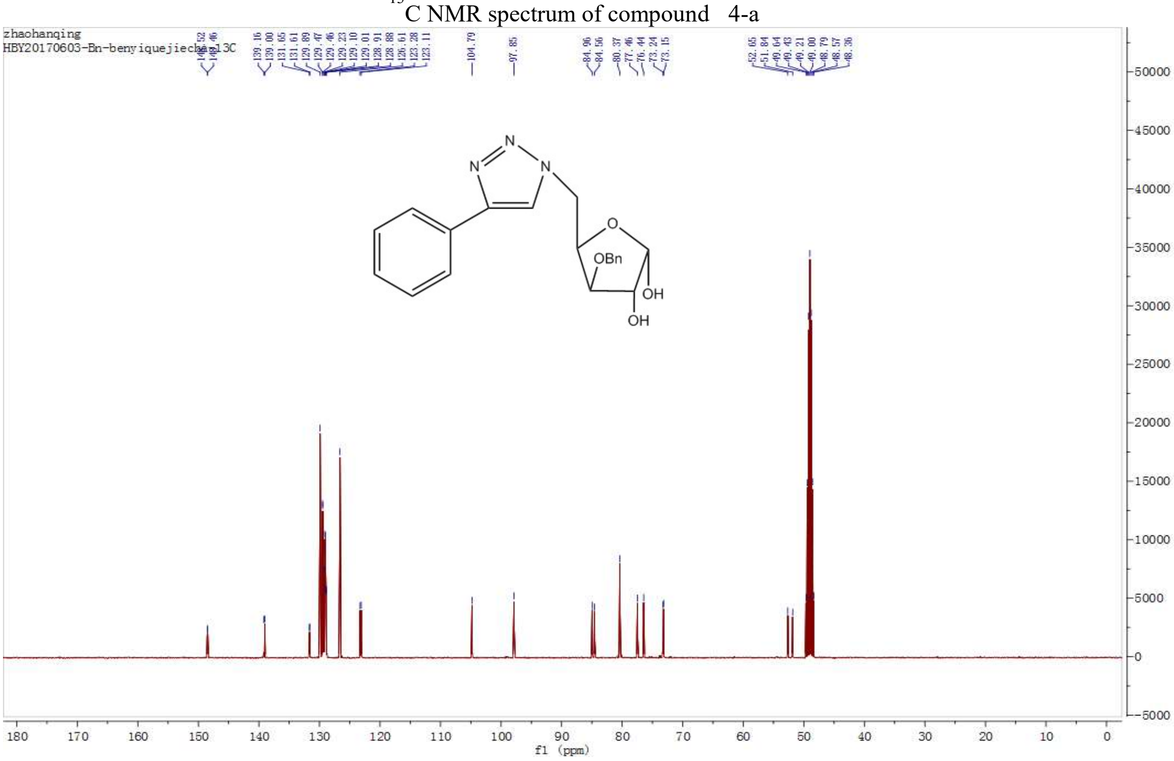

HRMS spectrum of compound **4-a**

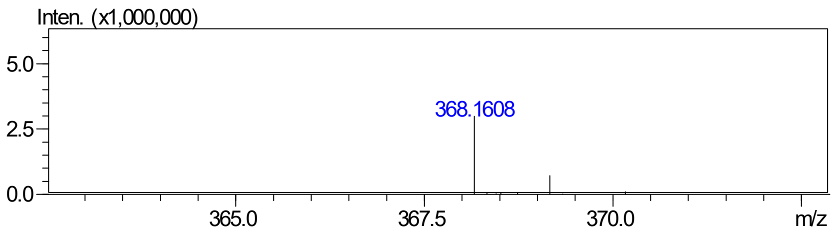

^{1}H NMR spectrum of compound 4-b

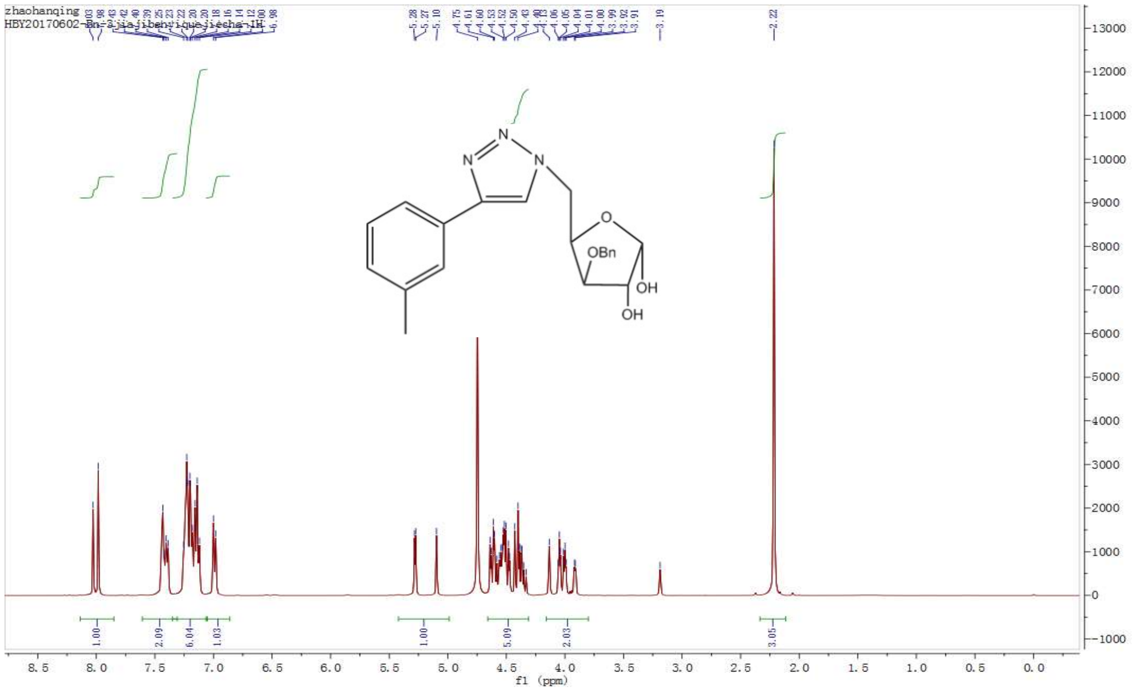

^{13}C NMR spectrum of compound 4-b

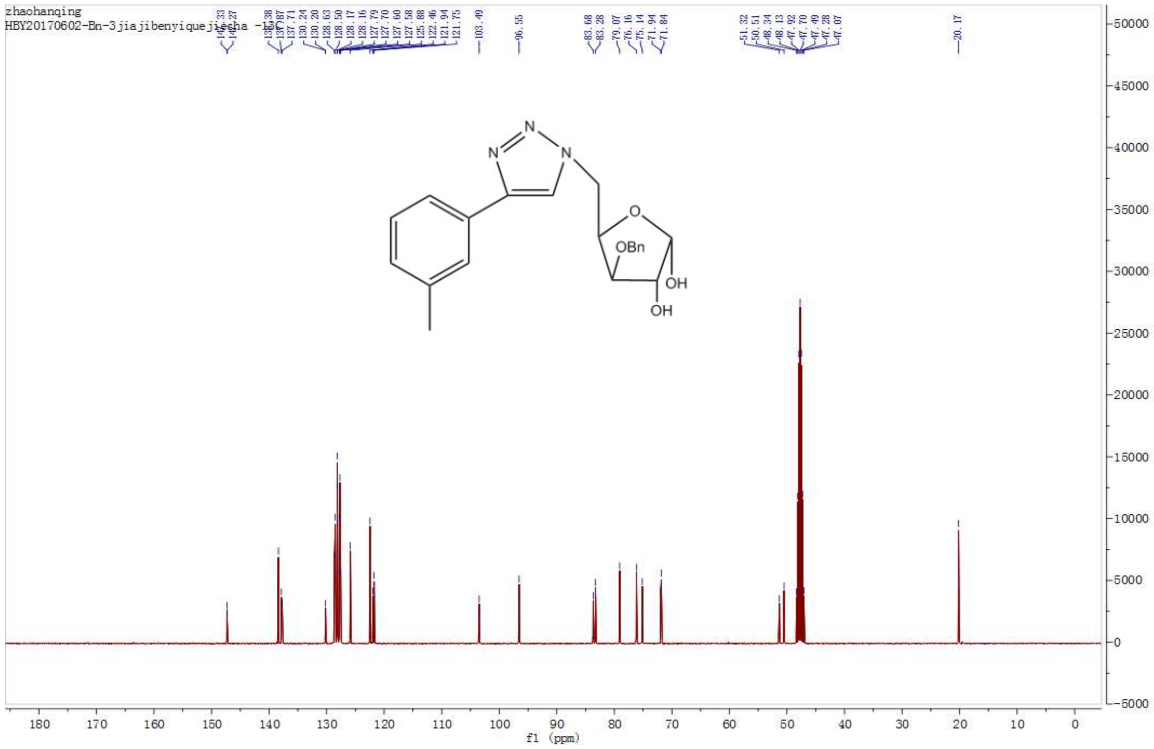

HRMS spectrum of compound **4-b**

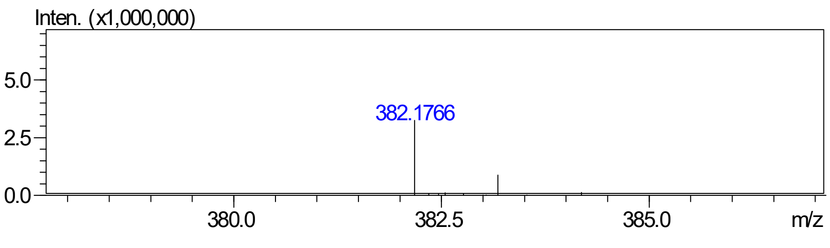

^{1}H NMR spectrum of compound 4-c

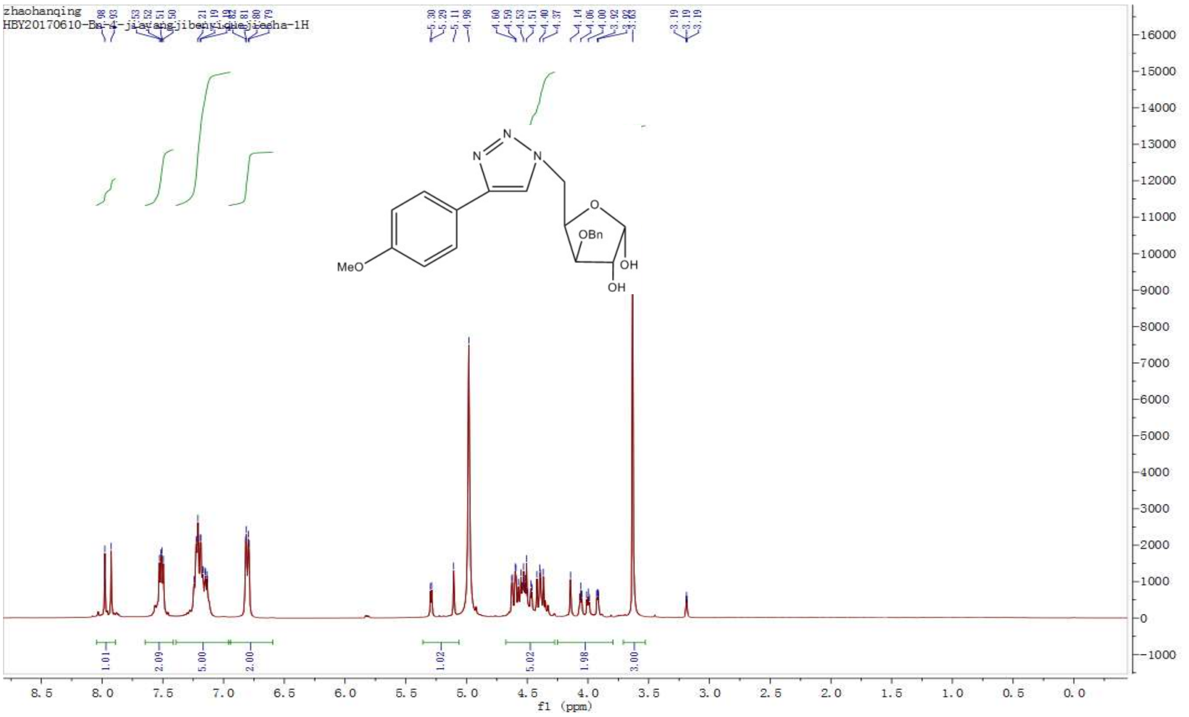

^{13}C NMR spectrum of compound 4-c

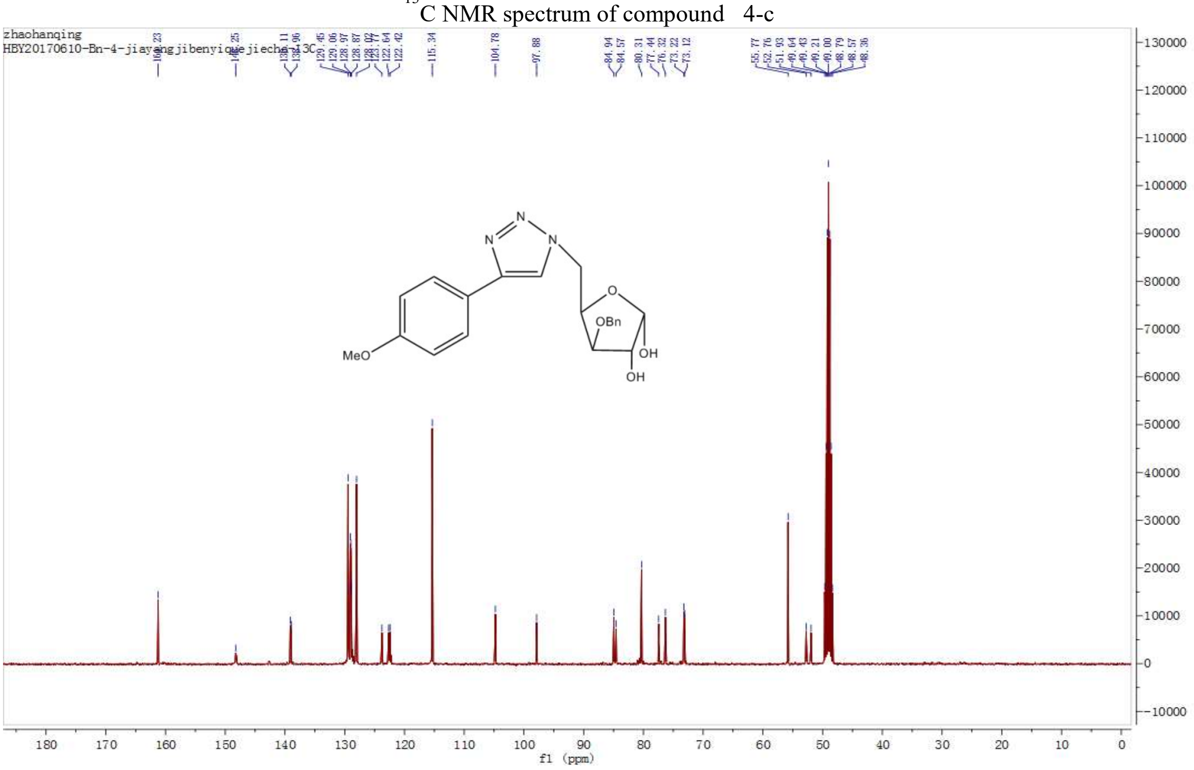

HRMS spectrum of compound **4-c**

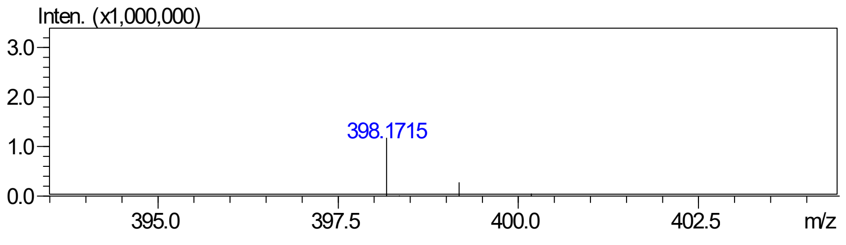

^{1}H NMR spectrum of compound 4-d

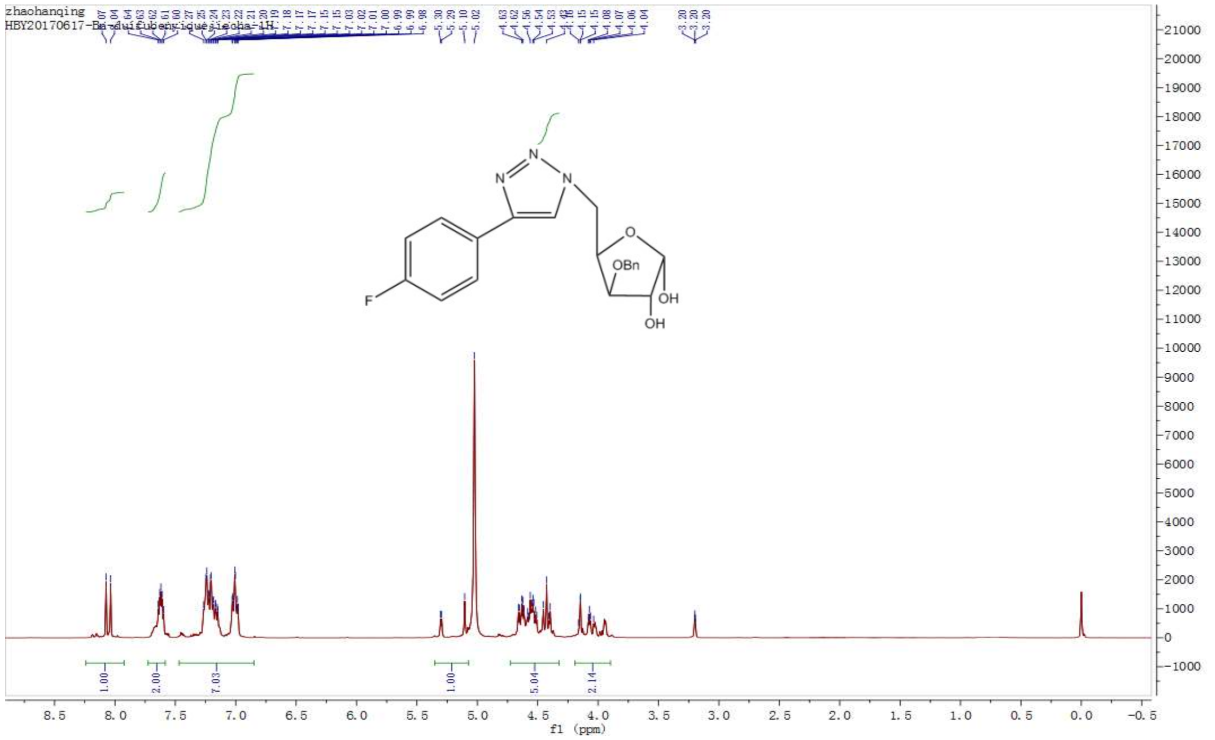

^{13}C NMR spectrum of compound 4-d

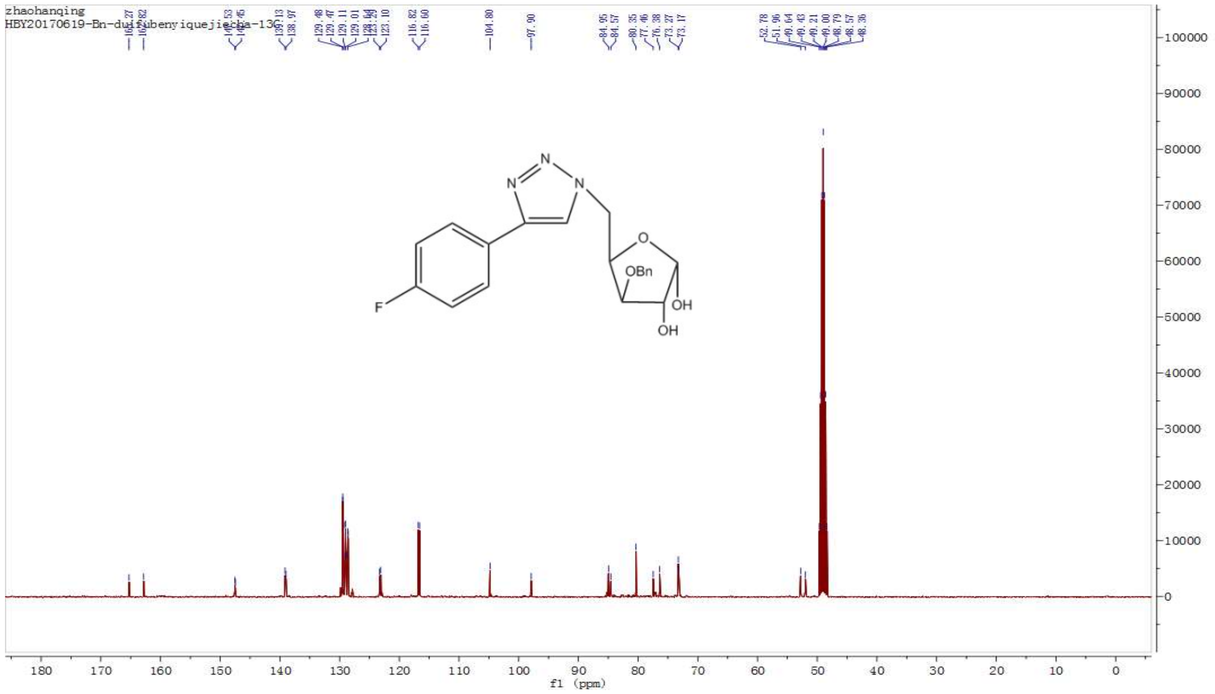

HRMS spectrum of compound **4-d**

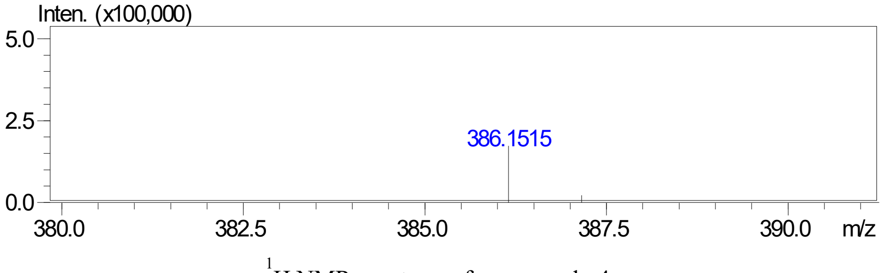

^{1}H NMR spectrum of compound 4-e

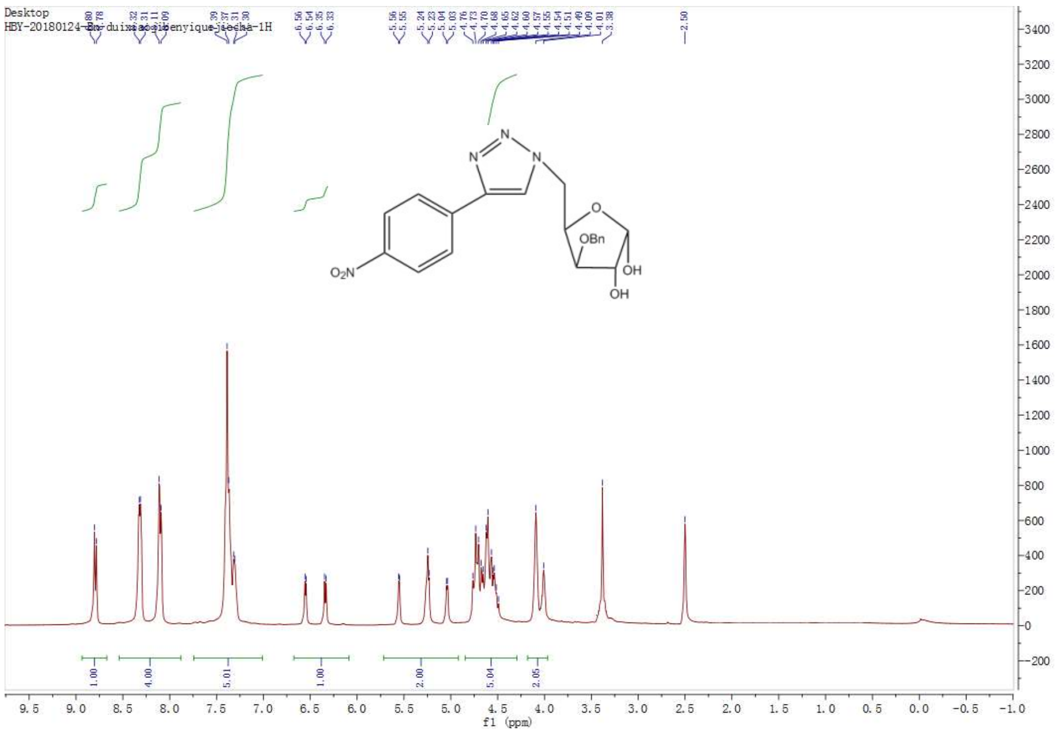

^{13}C NMR spectrum of compound 4-e

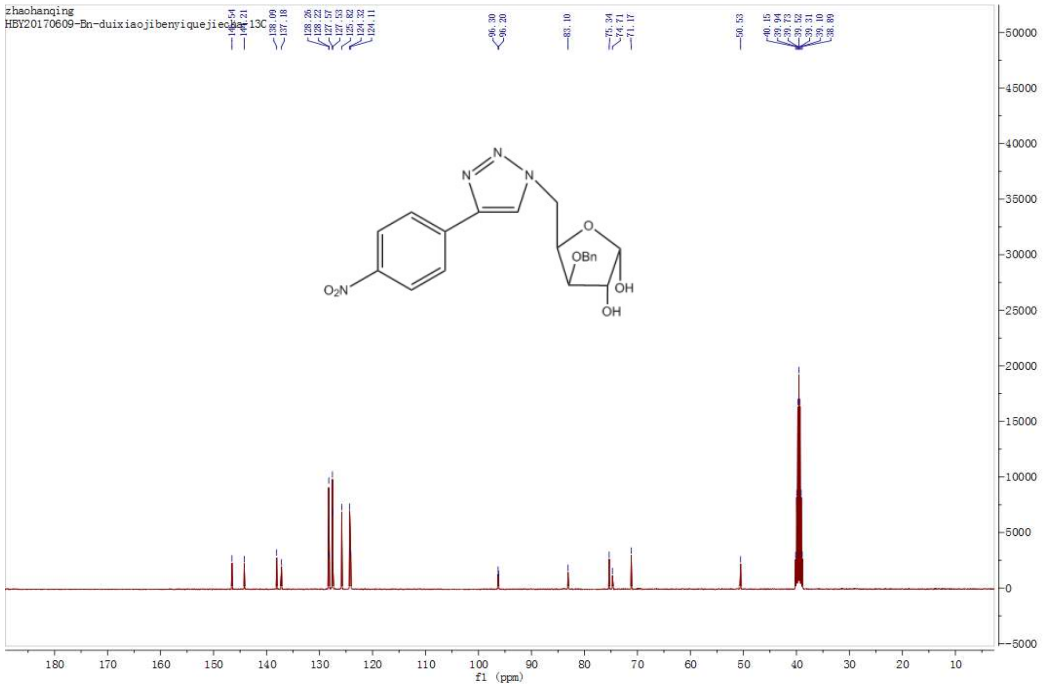

HRMS spectrum of compound **4-e**

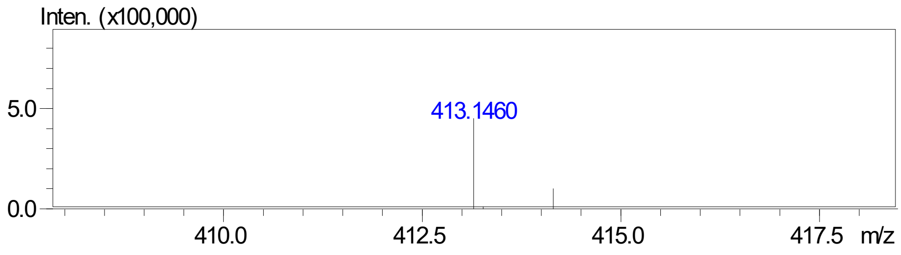

^{1}H NMR spectrum of compound 4-f

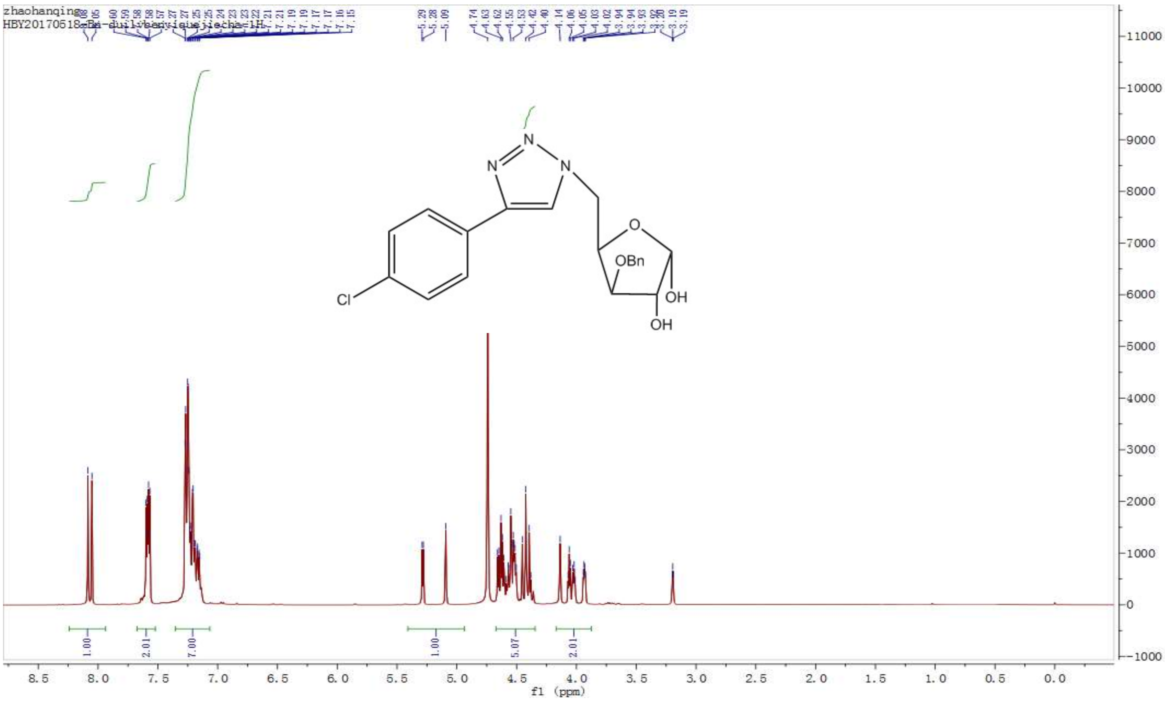

^{13}C NMR spectrum of compound 4-f

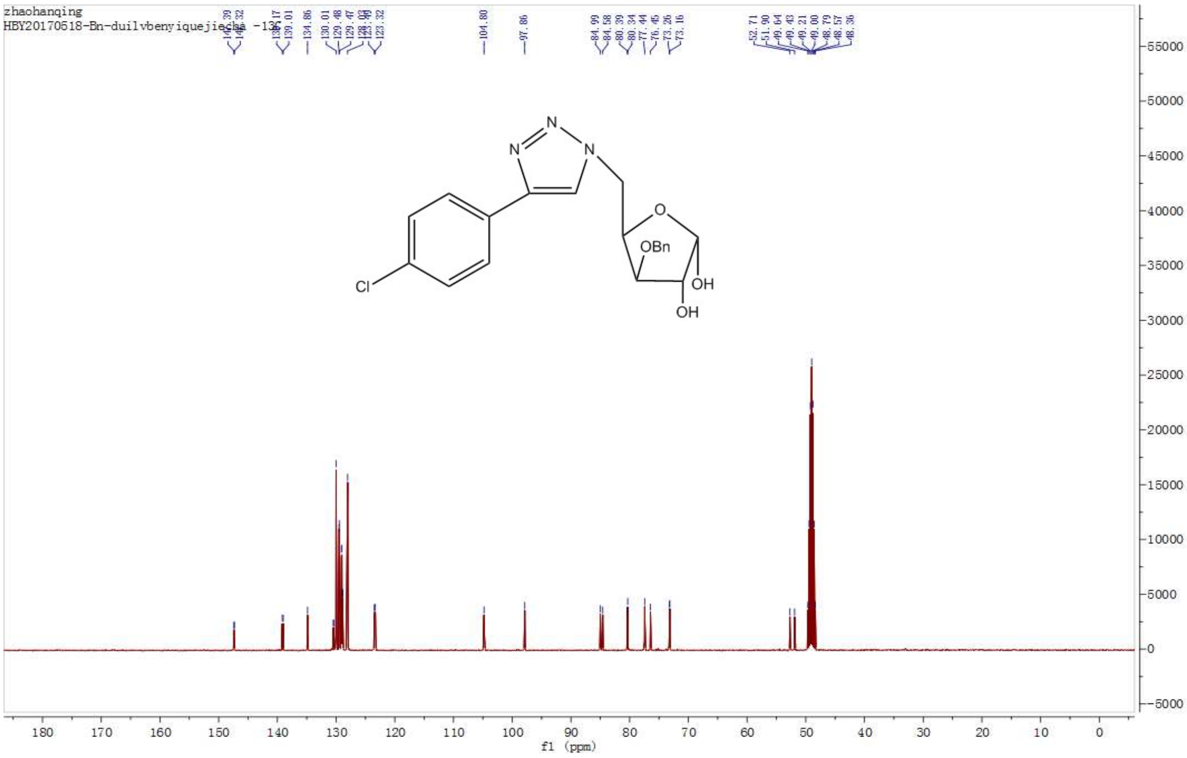

HRMS spectrum of compound **4-f**

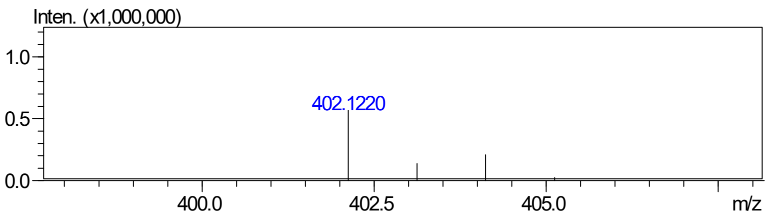

^{1}H NMR spectrum of compound 4-g

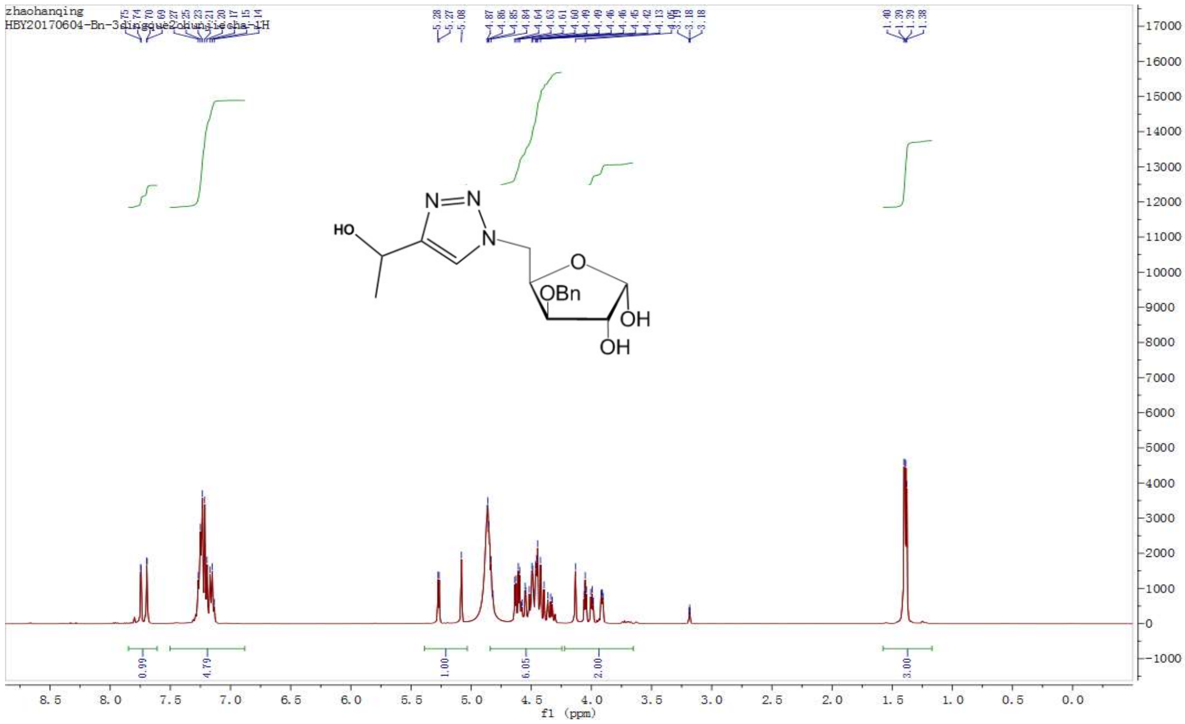

^{13}C NMR spectrum of compound 4-g

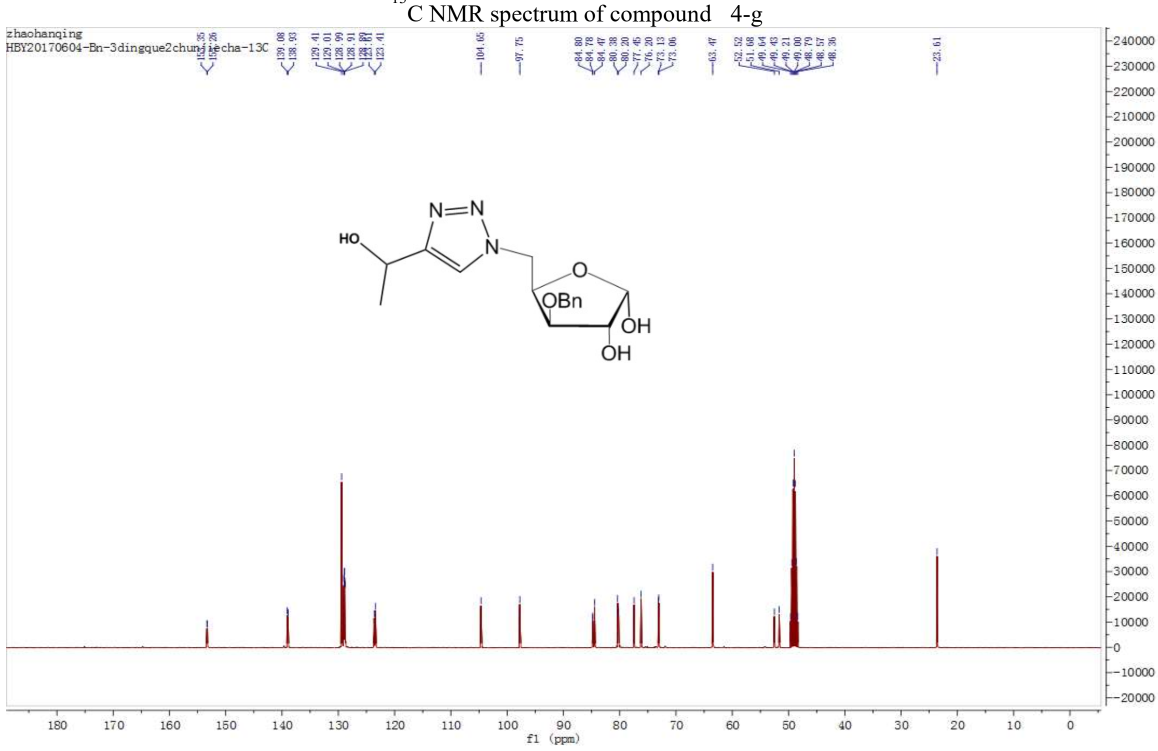

HRMS spectrum of compound 4-g

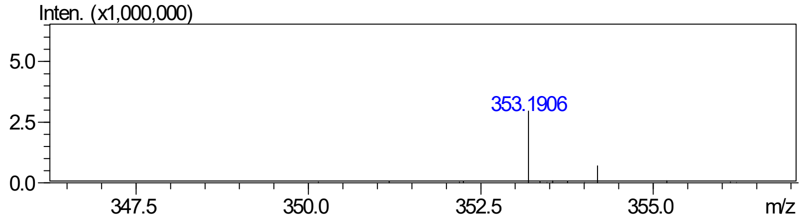

^{1}H NMR spectrum of compound 5-a

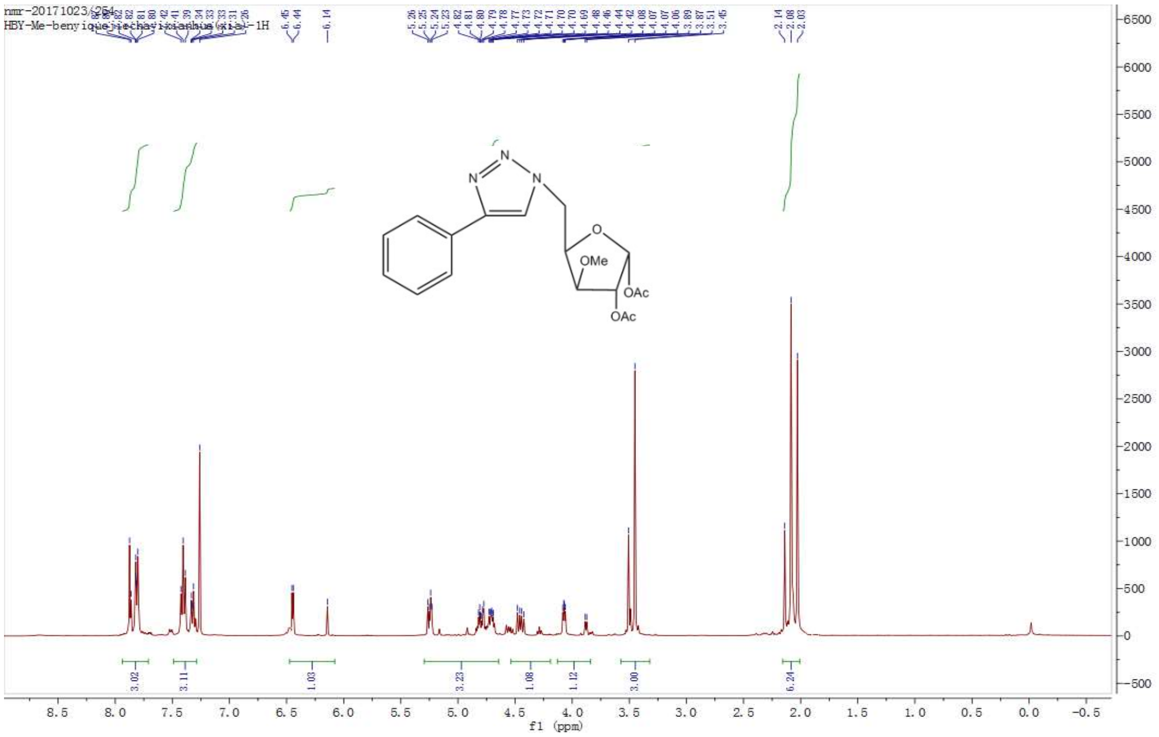

^{13}C NMR spectrum of compound 5-a

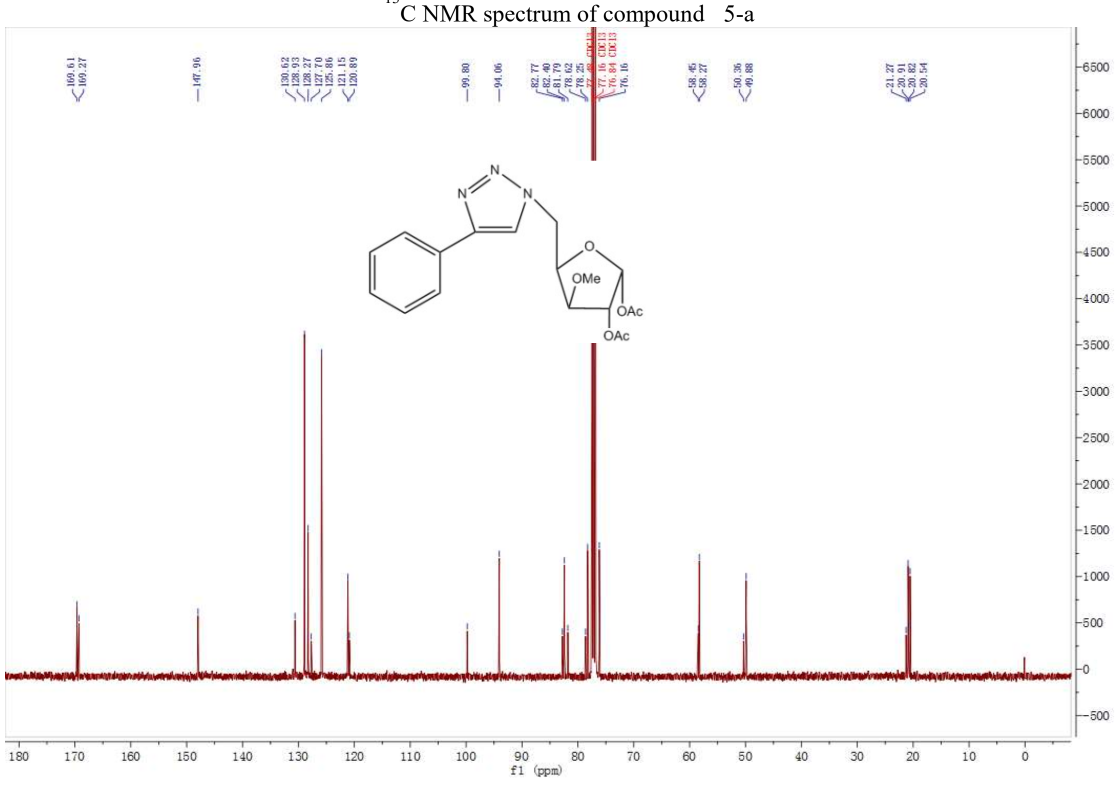

HRMS spectrum of compound 5-a

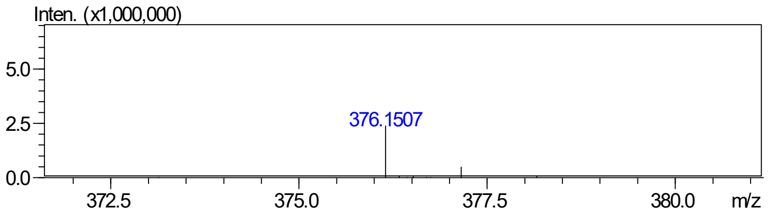

^{1}H NMR spectrum of compound 5-b

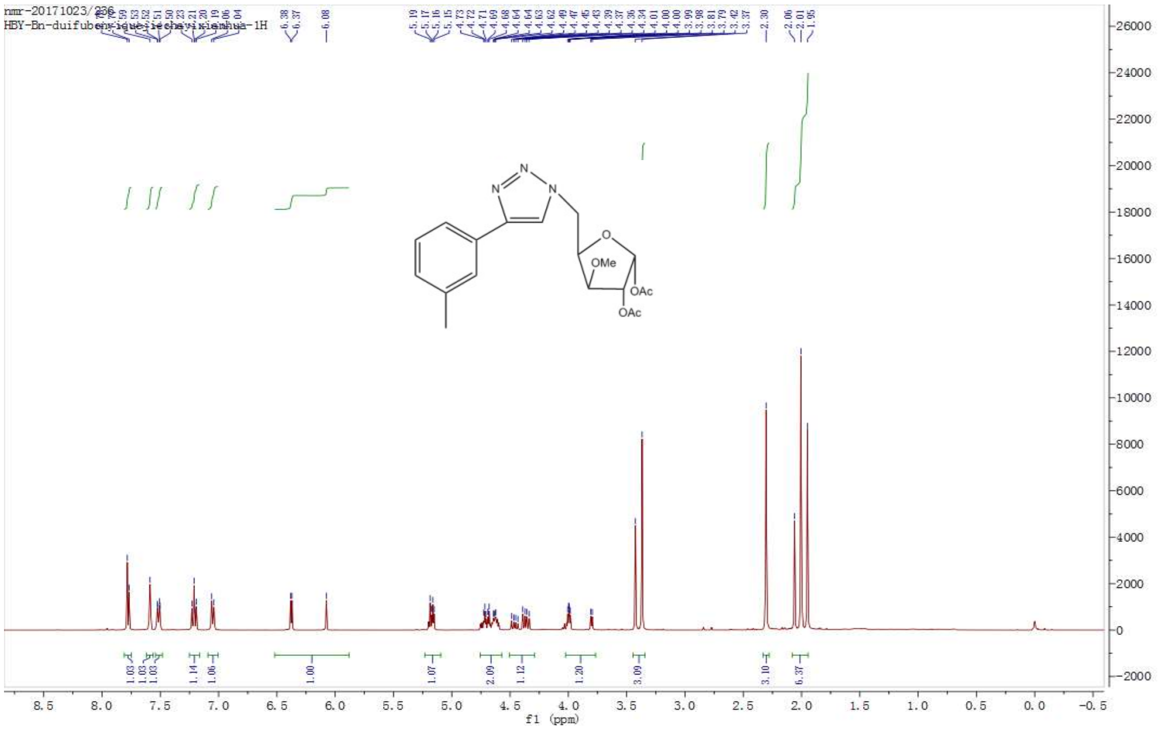

^{13}C NMR spectrum of compound 5-b

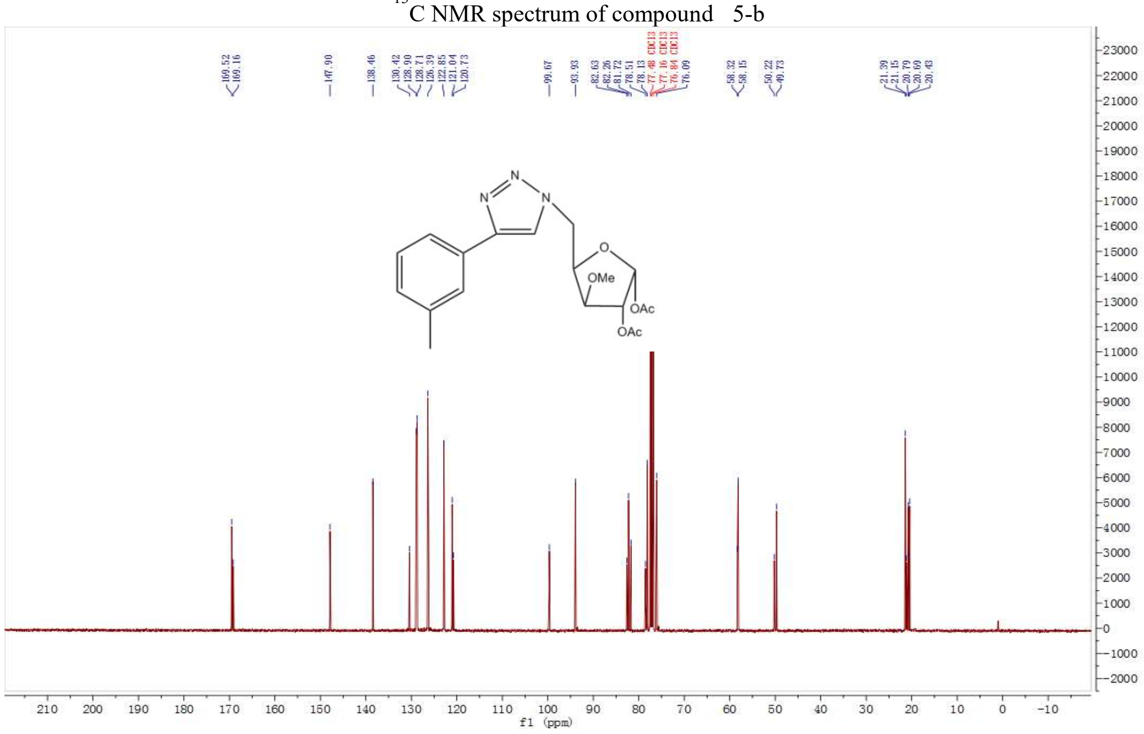

HRMS spectrum of compound 5-b

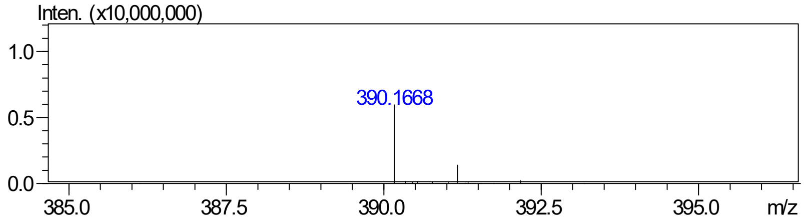

^{1}H NMR spectrum of compound 5-c

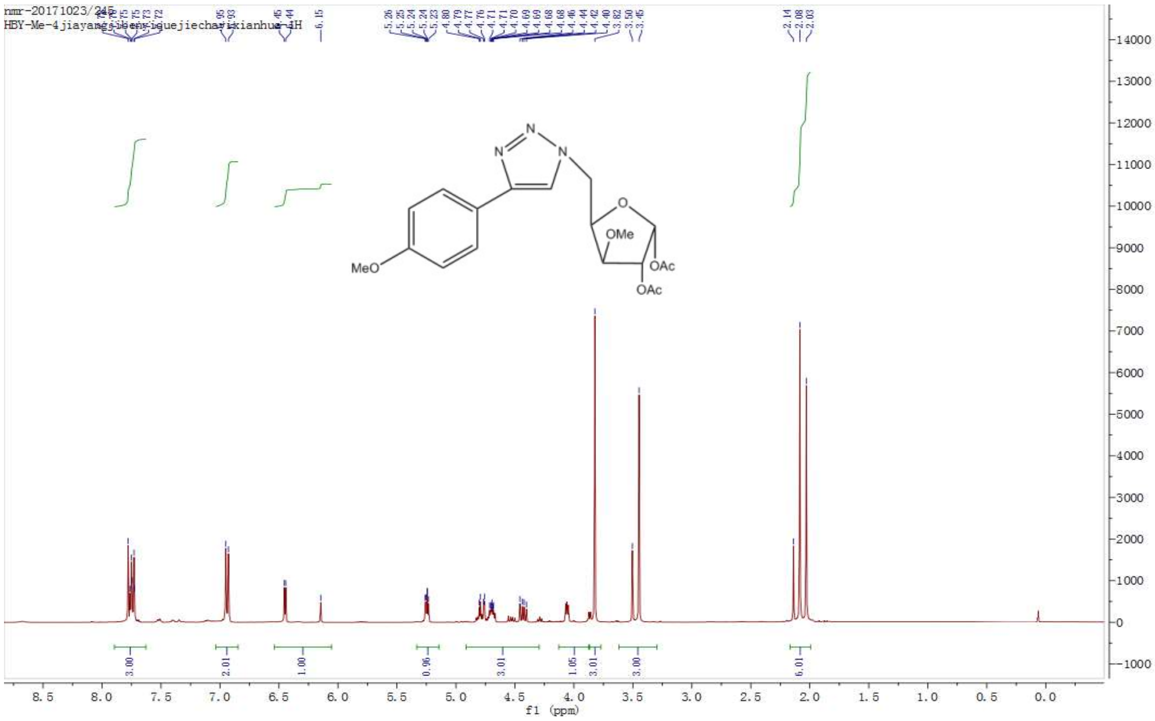

^{13}C NMR spectrum of compound 5-c

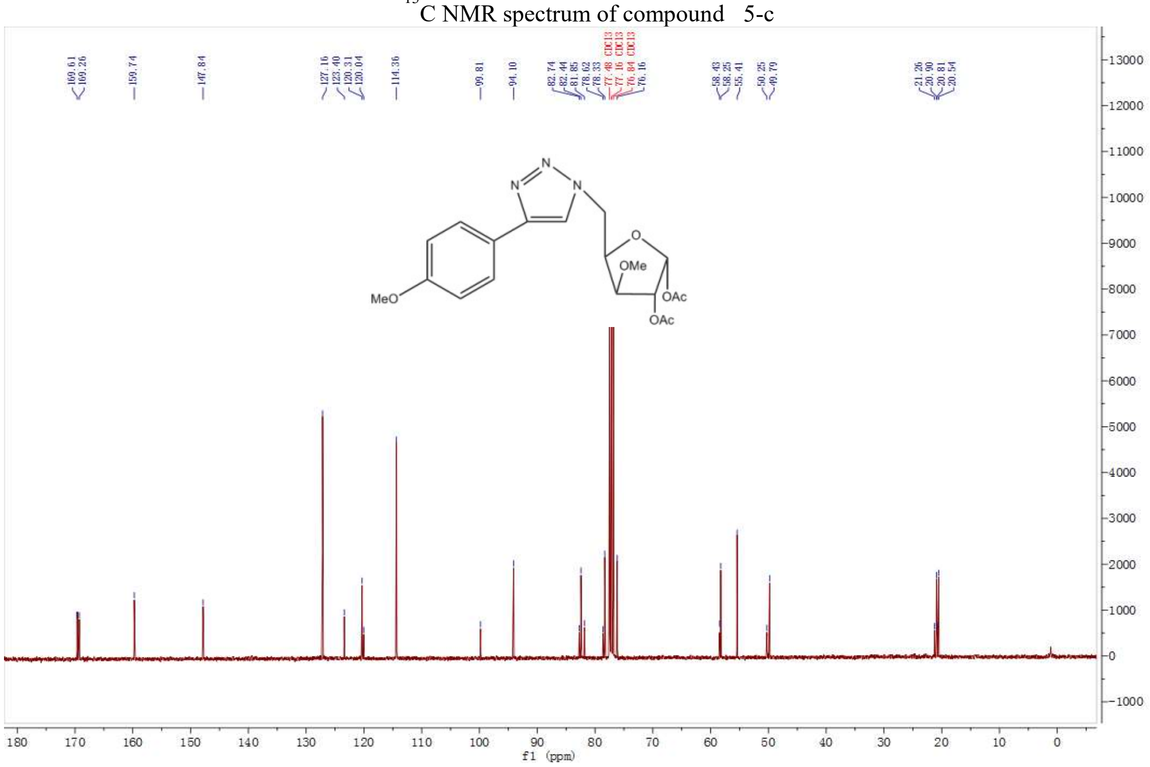

HRMS spectrum of compound 5-c

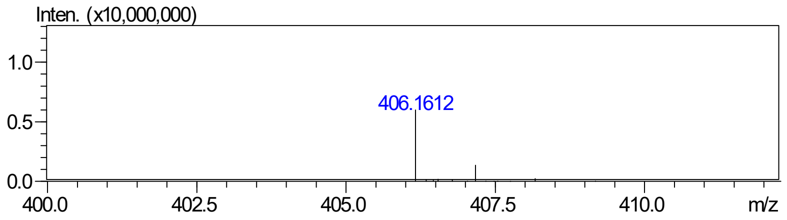

^{1}H NMR spectrum of compound 5-d

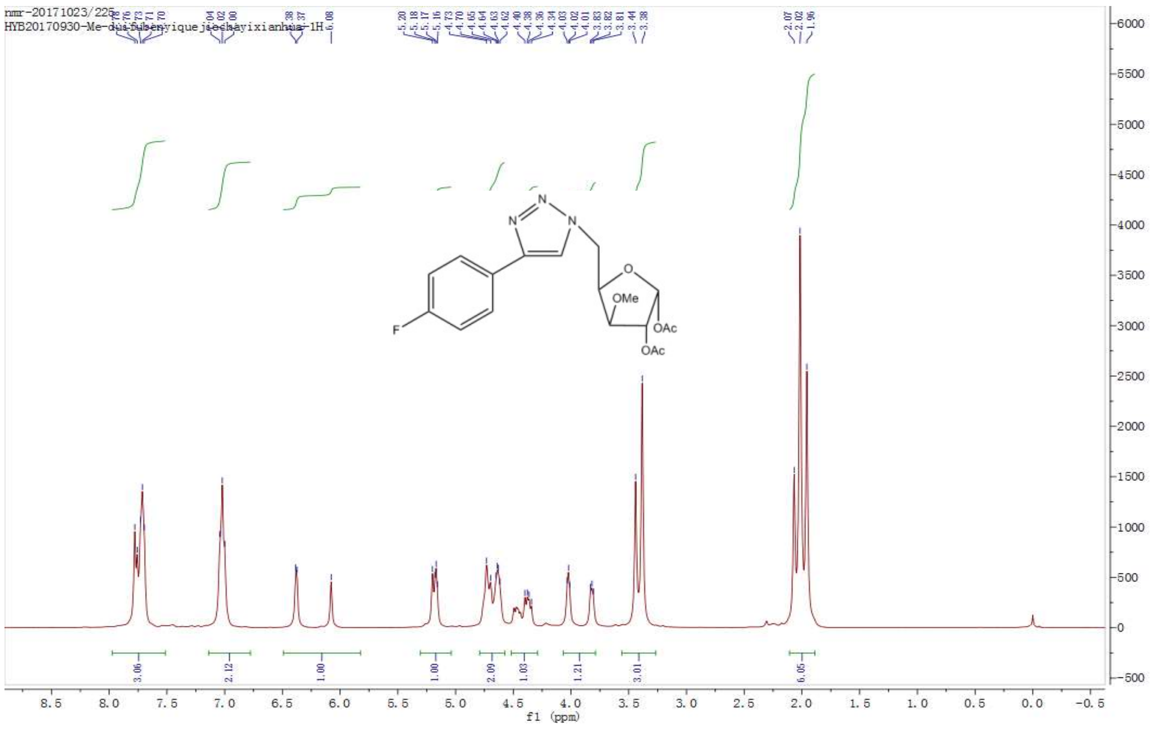

^{13}C NMR spectrum of compound 5-d

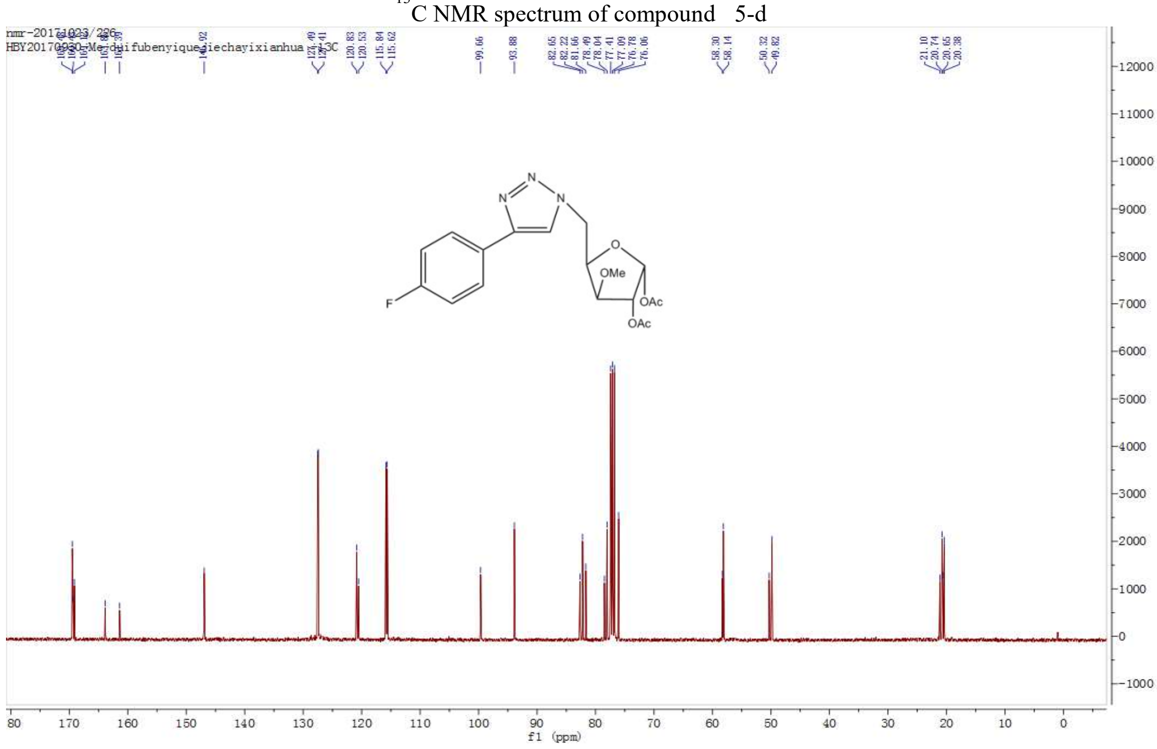

HRMS spectrum of compound 5-d

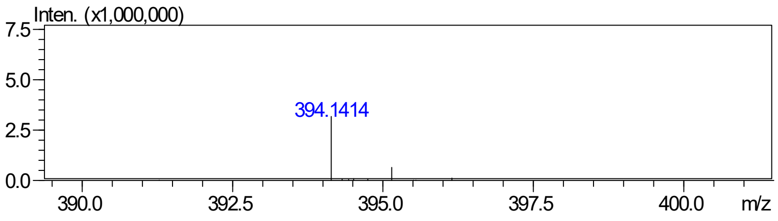

^{1}H NMR spectrum of compound 5-e

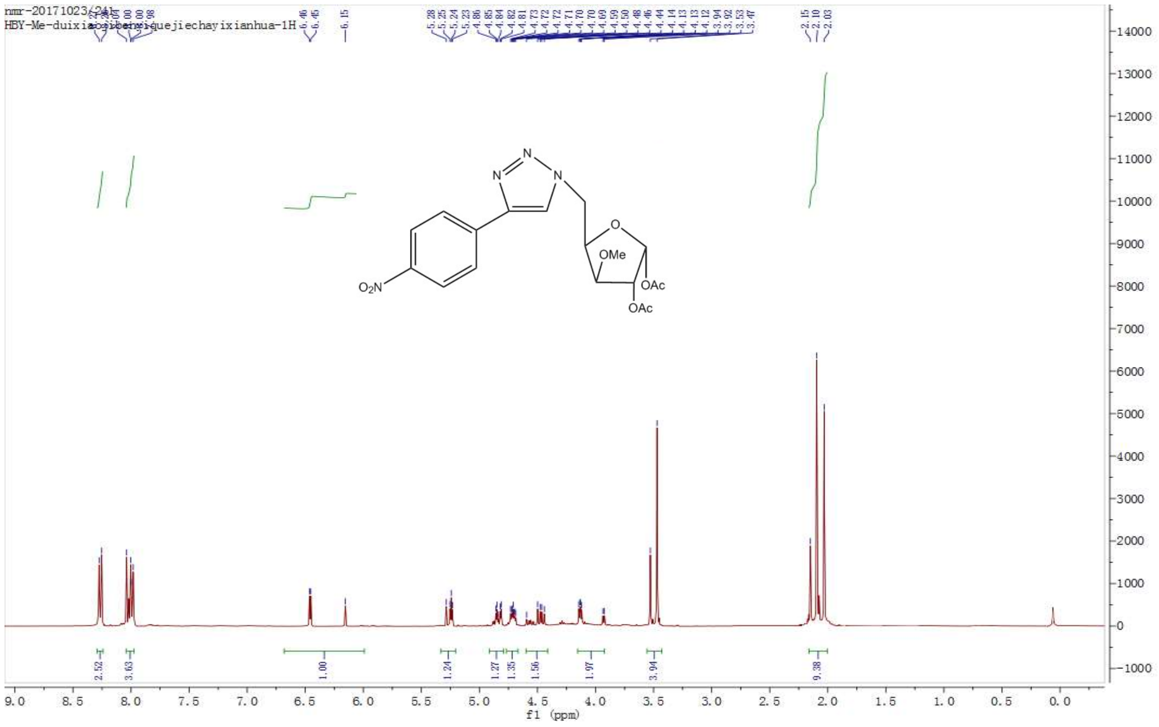

^{13}C NMR spectrum of compound 5-e

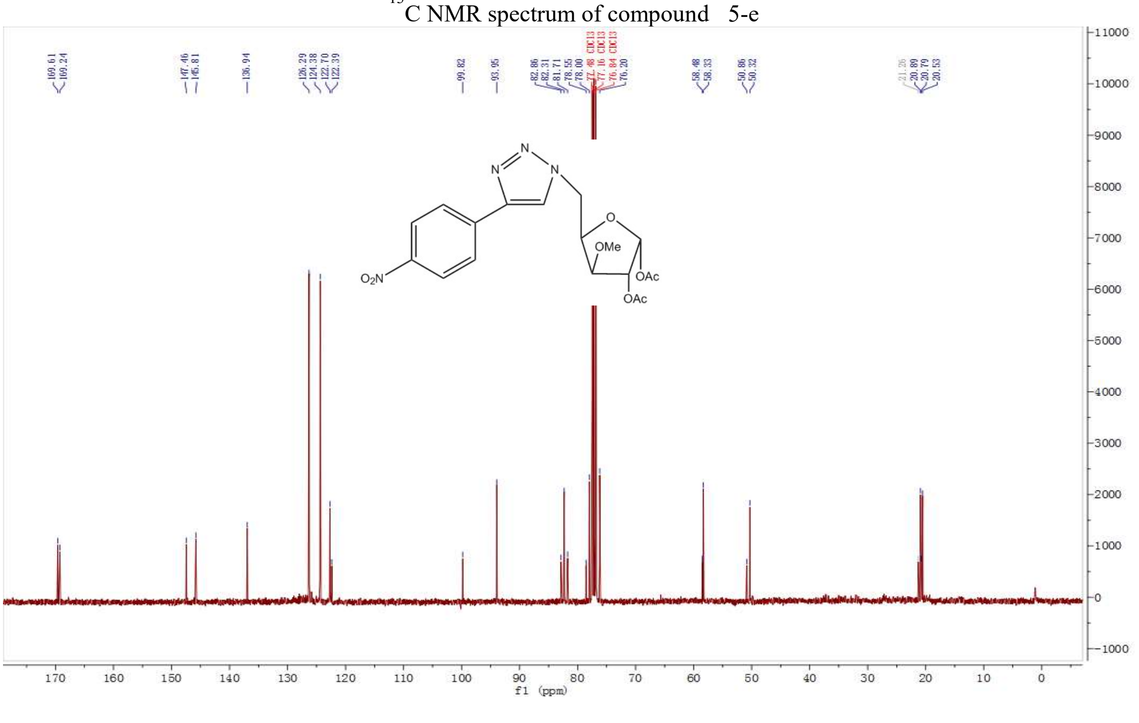

HRMS spectrum of compound 5-e

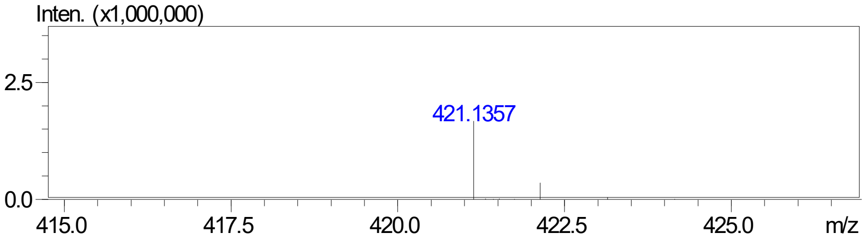

^{1}H NMR spectrum of compound 5-f

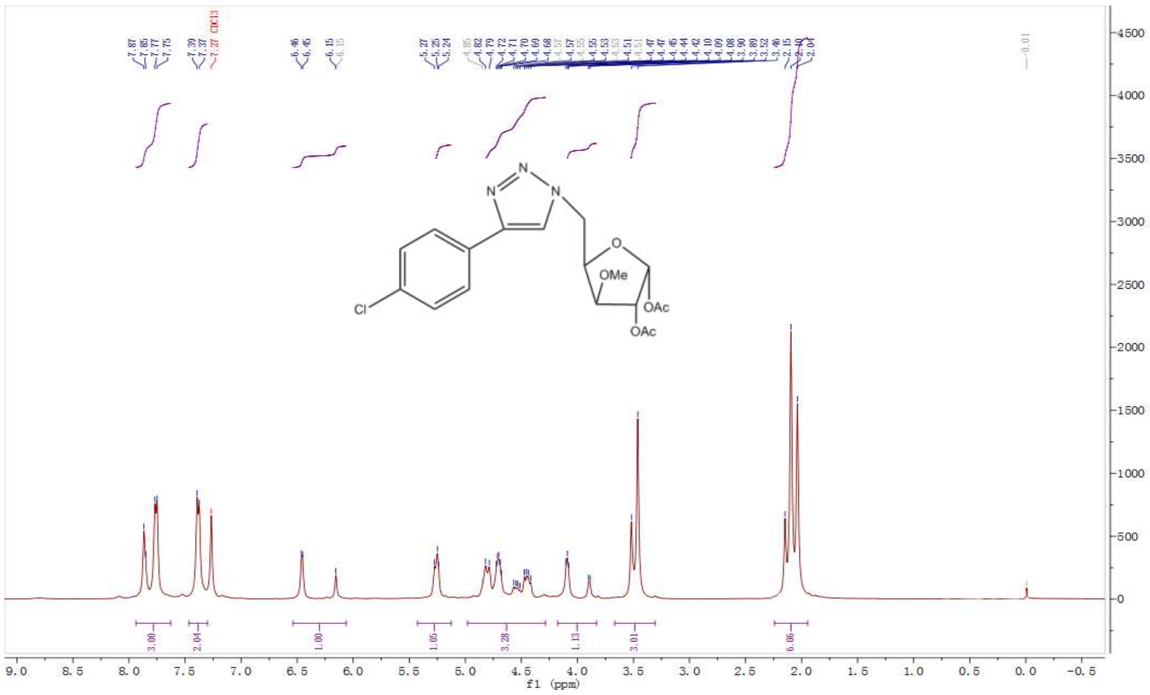

^{13}C NMR spectrum of compound 5-f

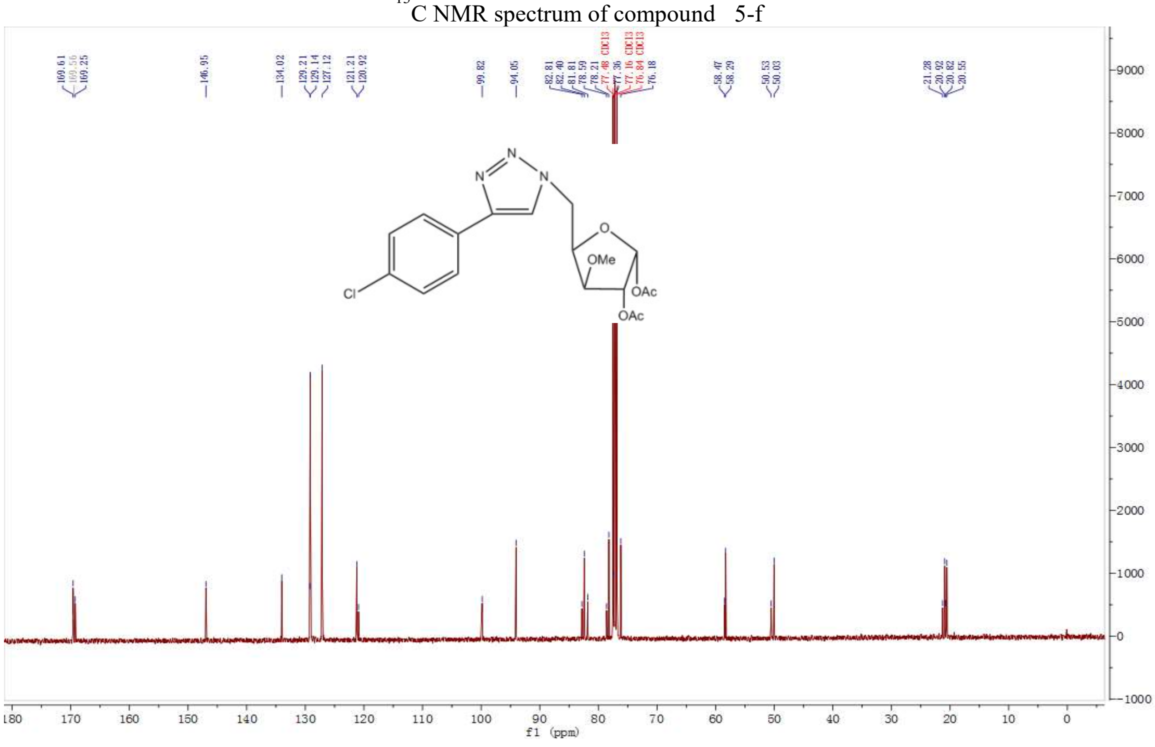

HRMS spectrum of compound 5-f

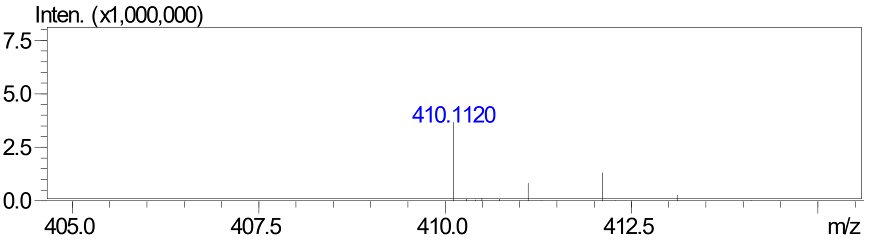

^{1}H NMR spectrum of compound 6-a

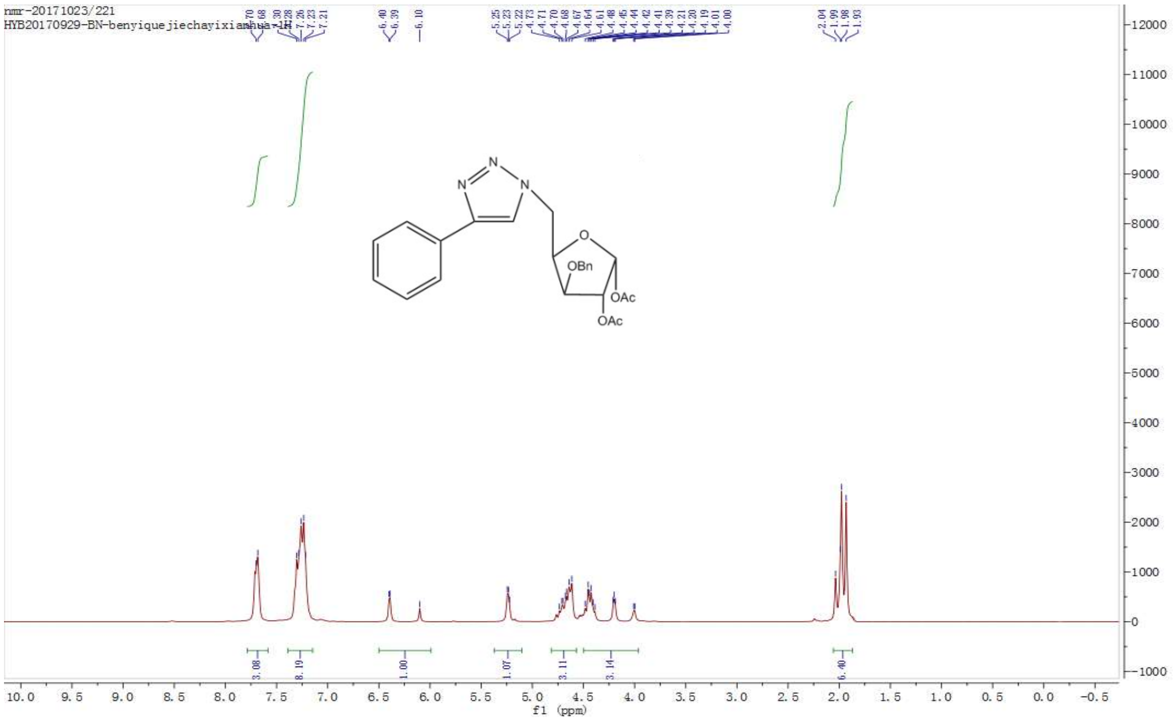

^{13}C NMR spectrum of compound 6-a

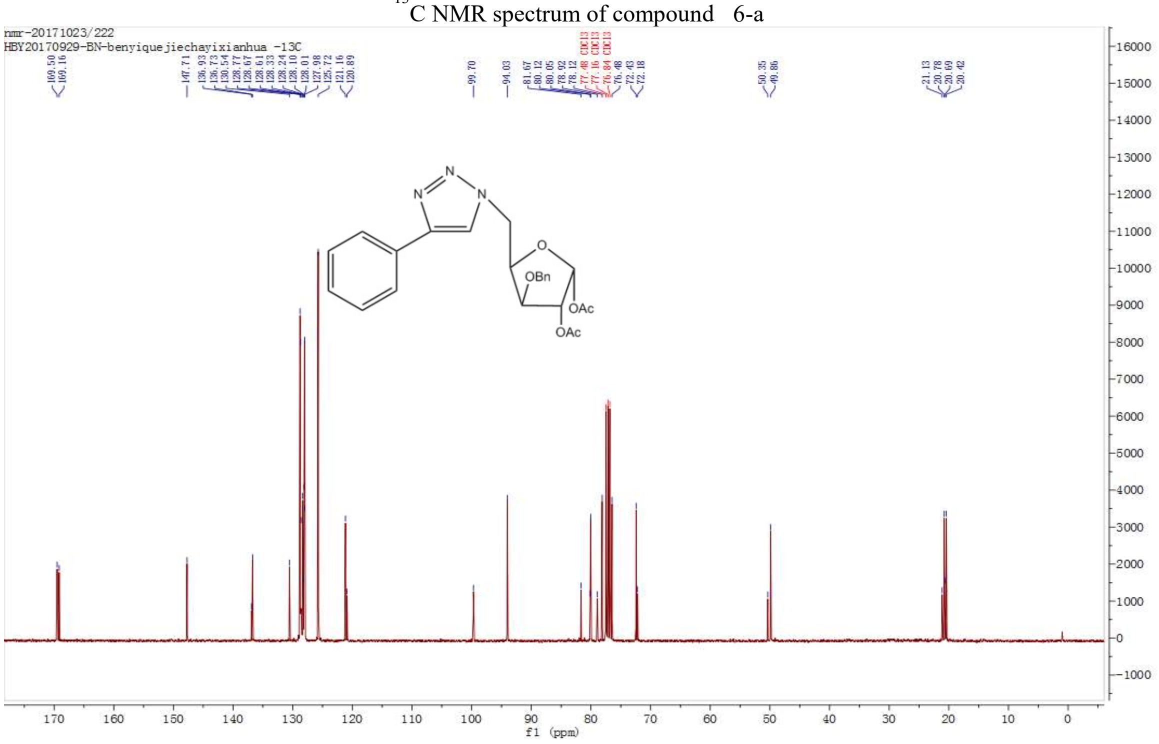

HRMS spectrum of compound 6-a

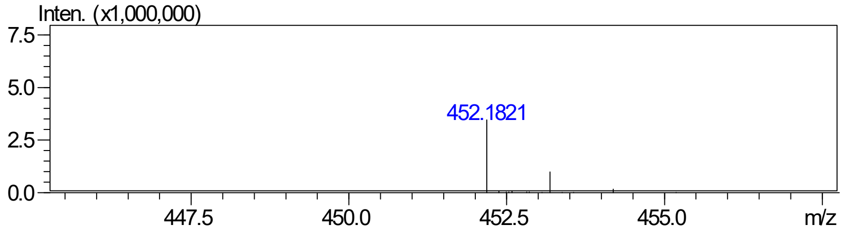

^{1}H NMR spectrum of compound 6-b

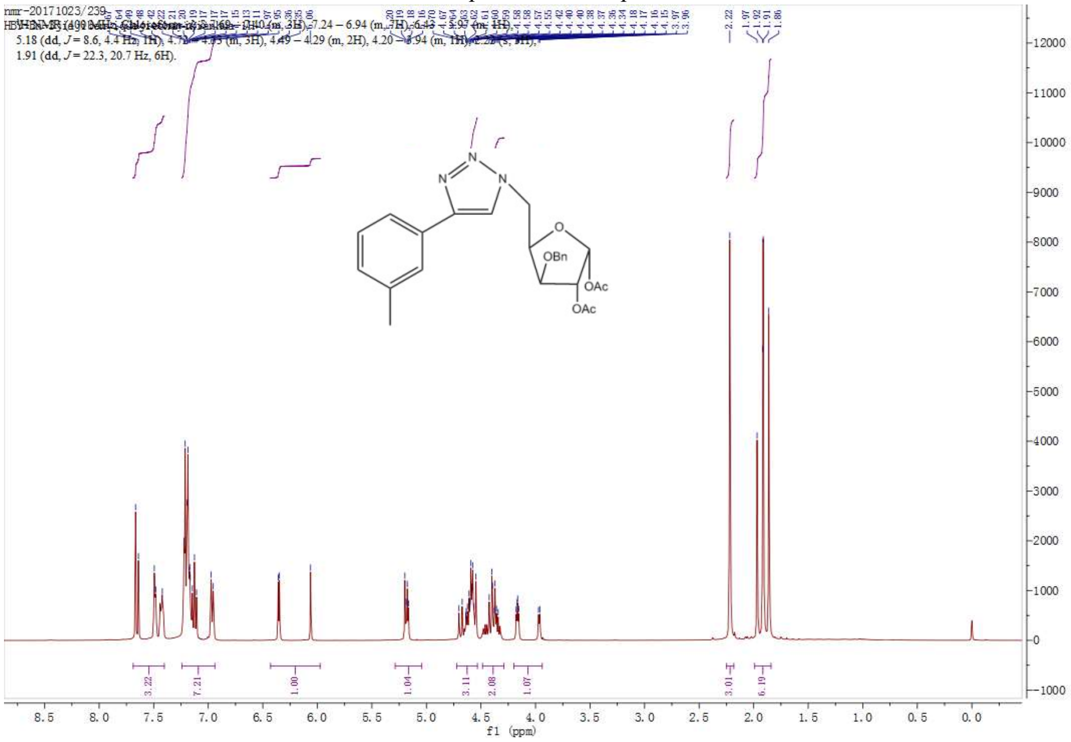

^{13}C NMR spectrum of compound 6-b

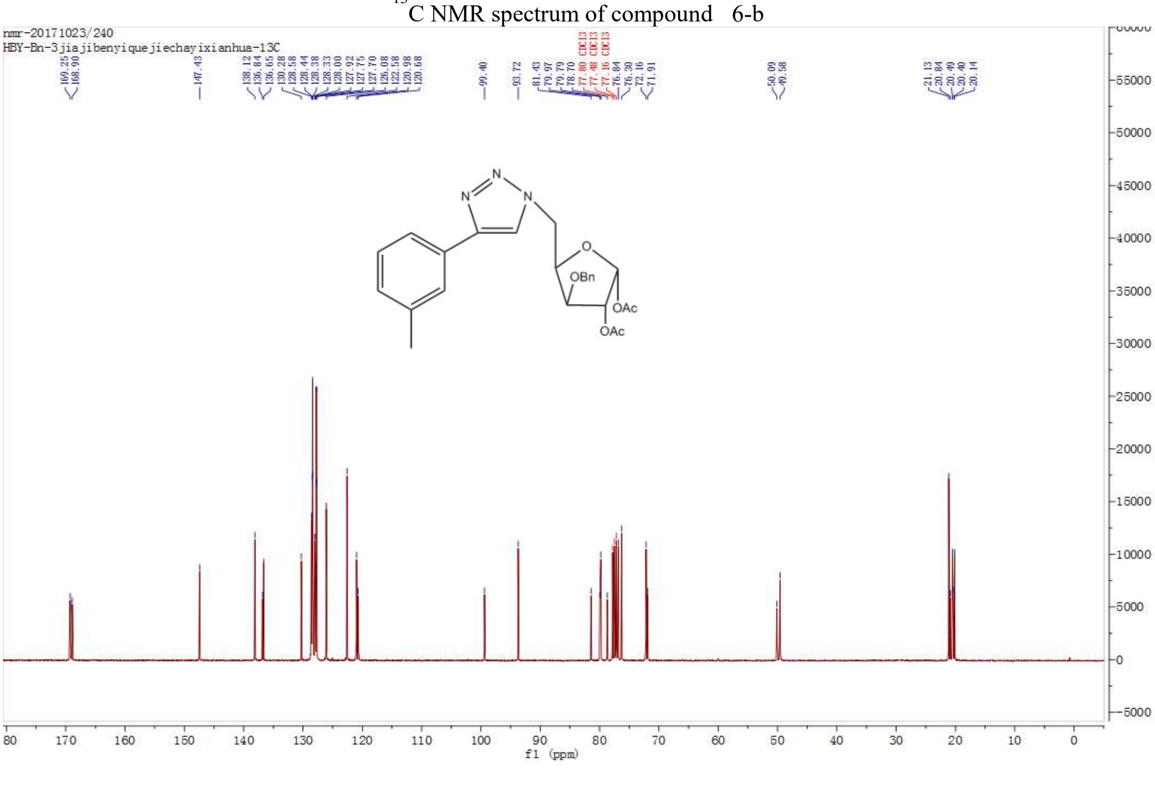

HRMS spectrum of compound 6-b

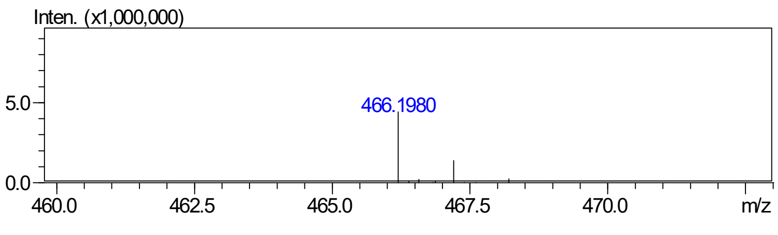

^{1}H NMR spectrum of compound 6-c

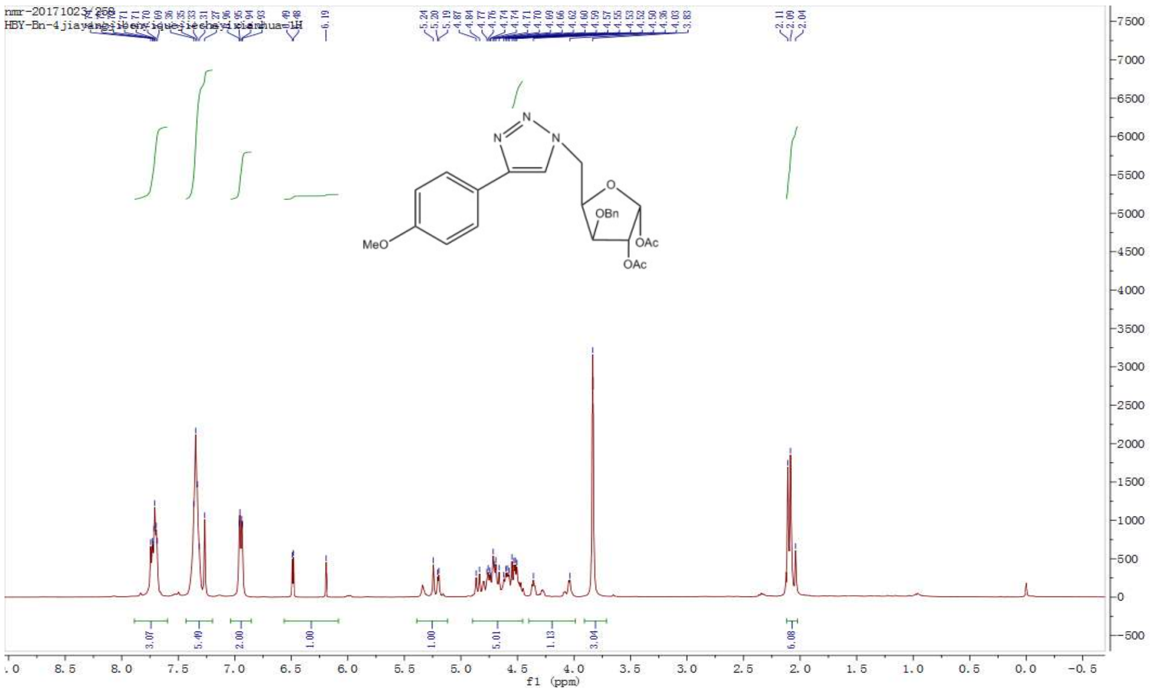

^{13}C NMR spectrum of compound 6-c

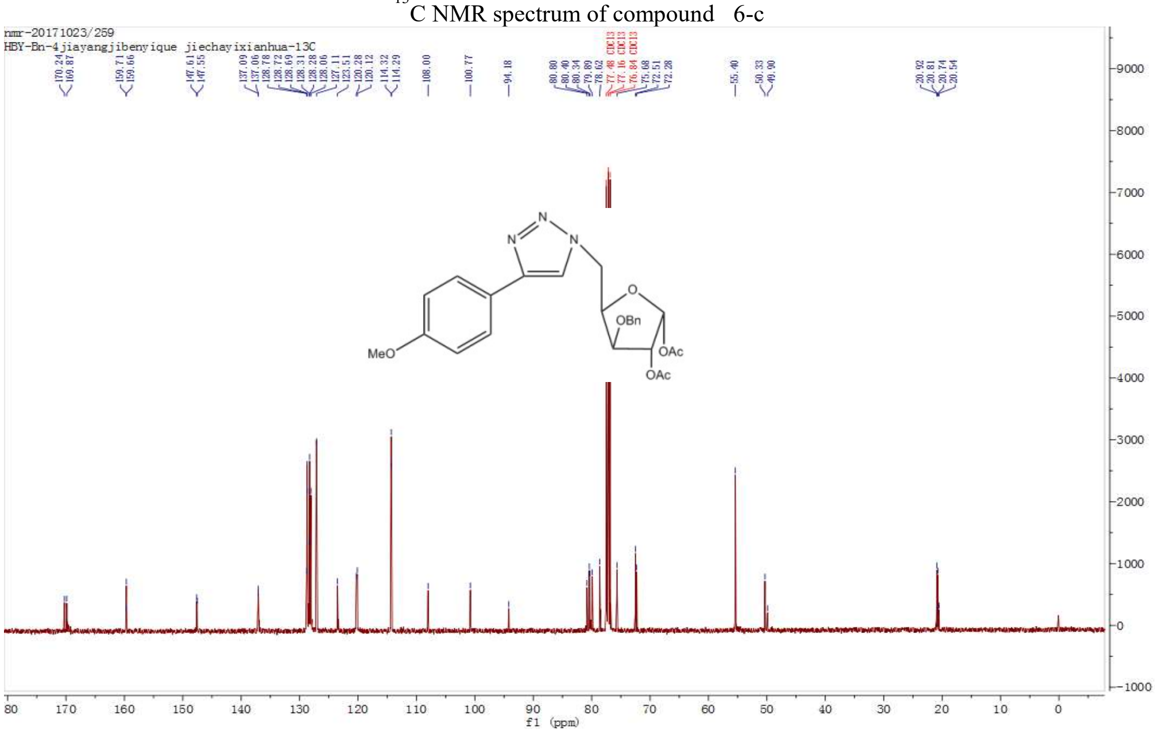

HRMS spectrum of compound 6-c

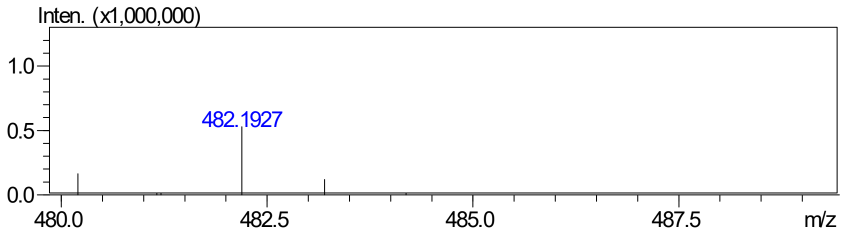

^{1}H NMR spectrum of compound 6-d

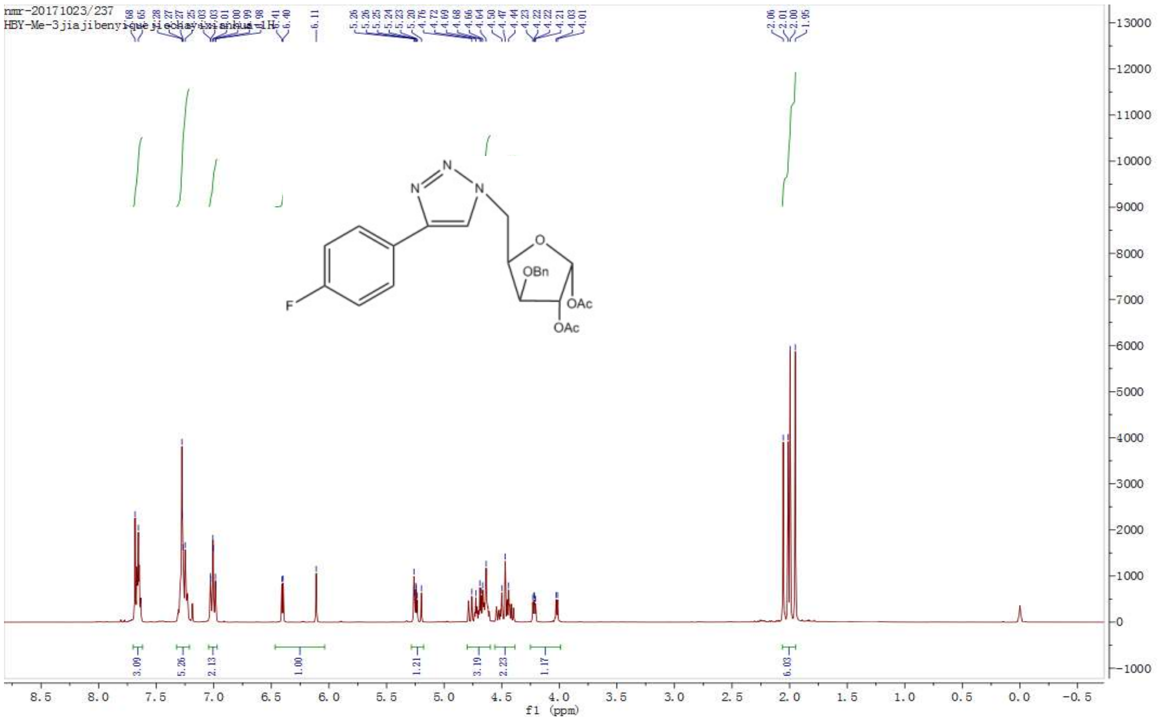

^{13}C NMR spectrum of compound 6-d

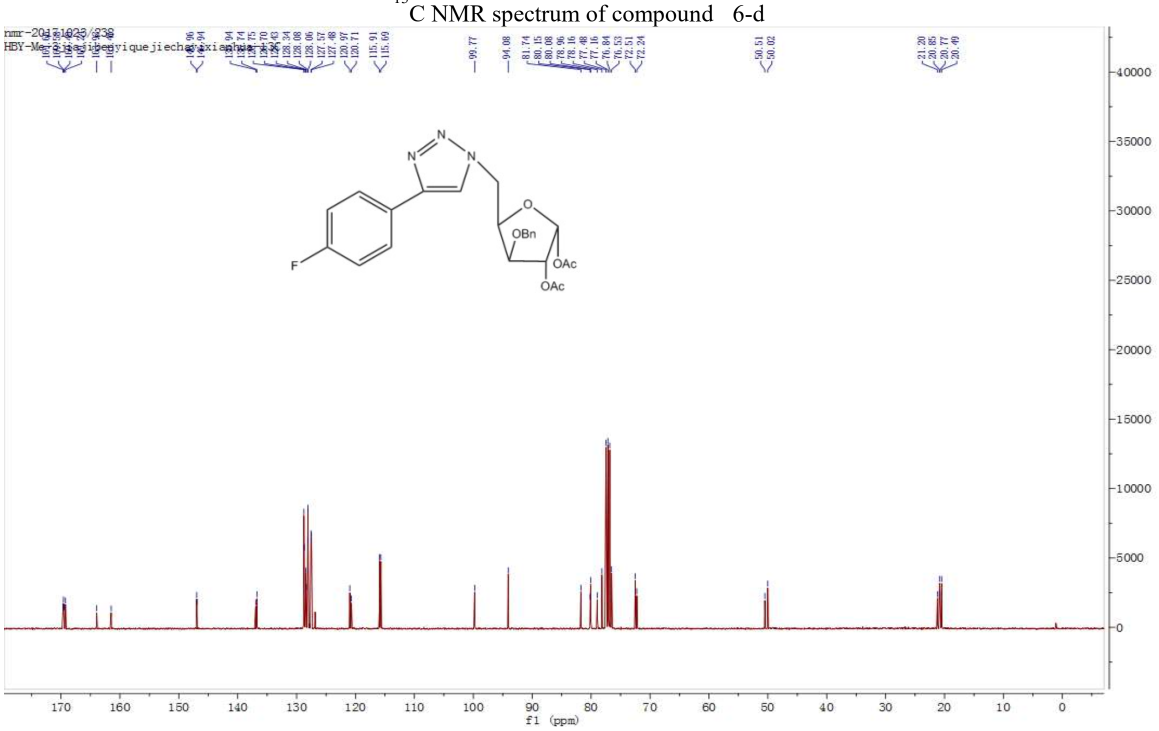

HRMS spectrum of compound 6-d

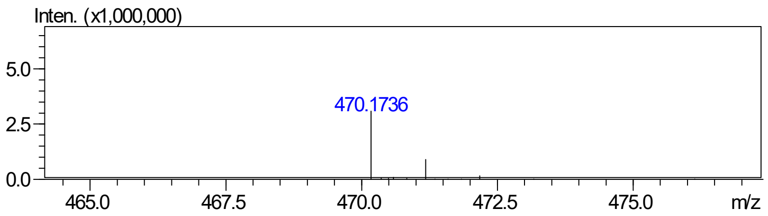

^{1}H NMR spectrum of compound 6-e

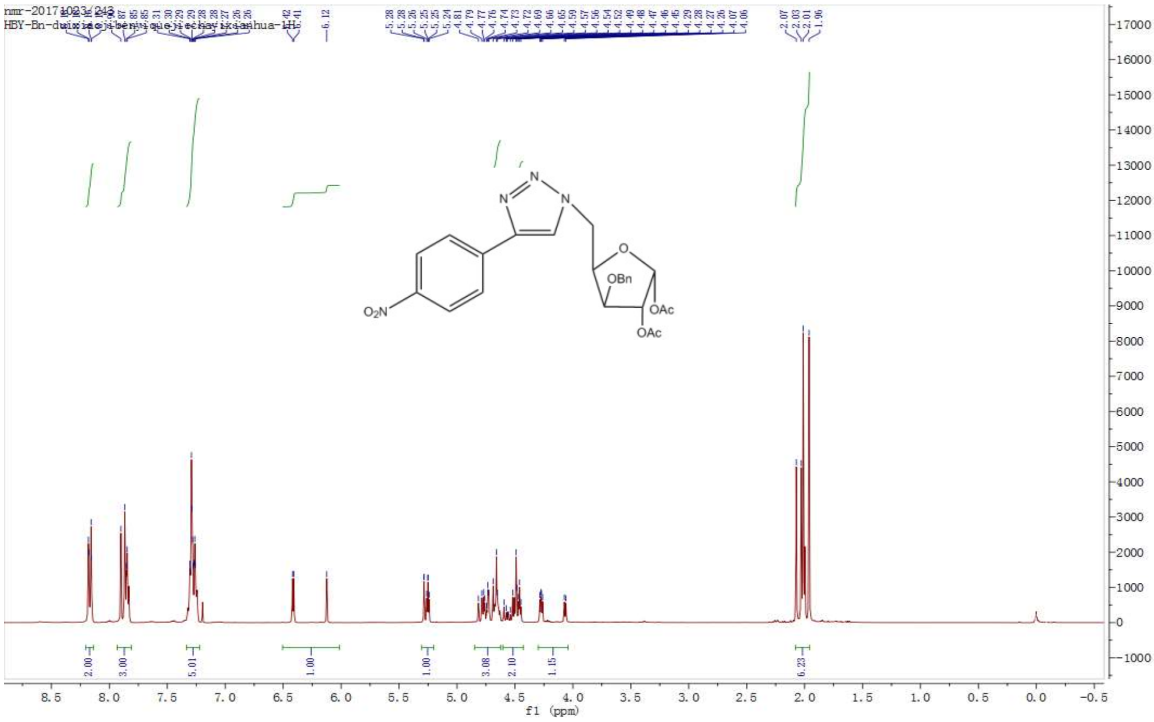

^{13}C NMR spectrum of compound 6-e

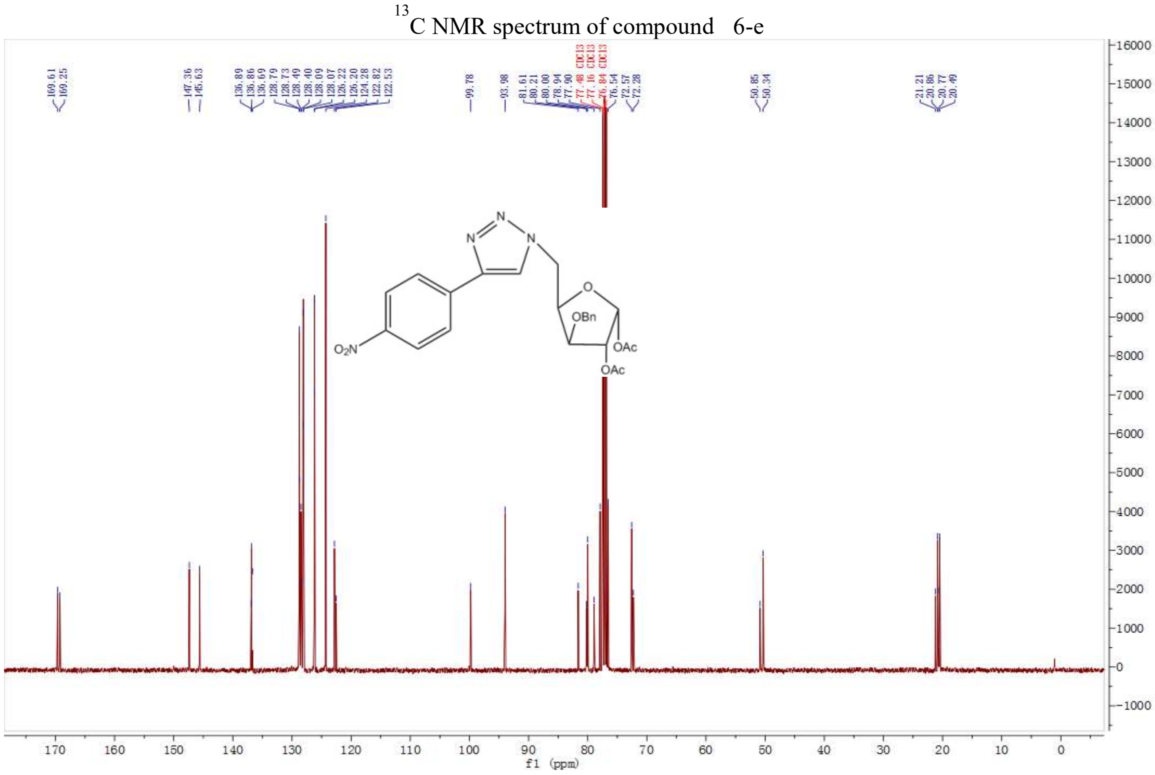

HRMS spectrum of compound 6-e

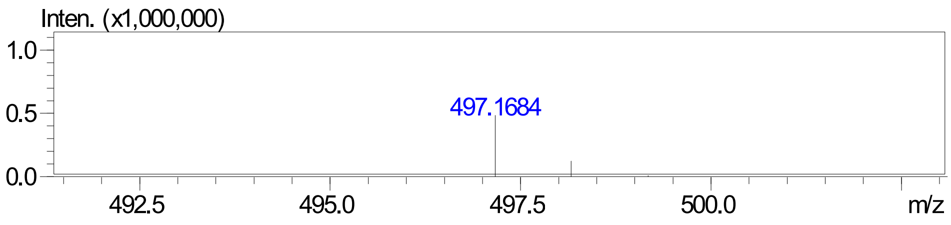

^{1}H NMR spectrum of compound 6-f

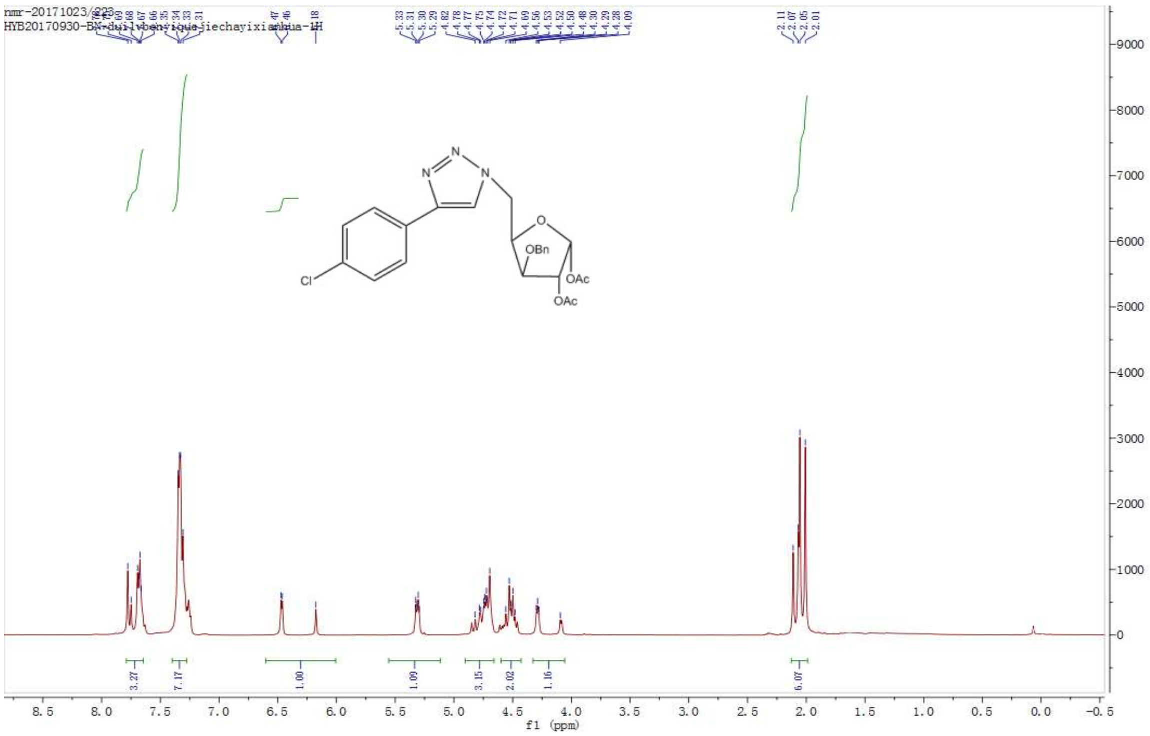

^{13}C NMR spectrum of compound 6-f

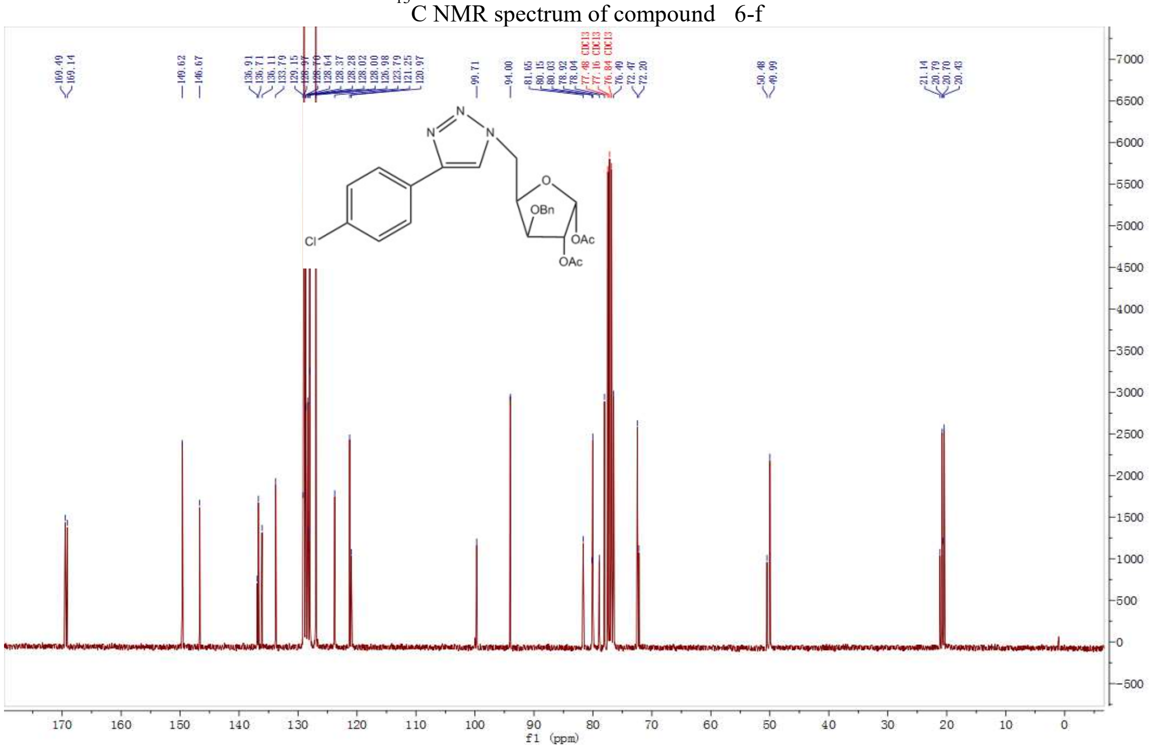

HRMS spectrum of compound 6-f

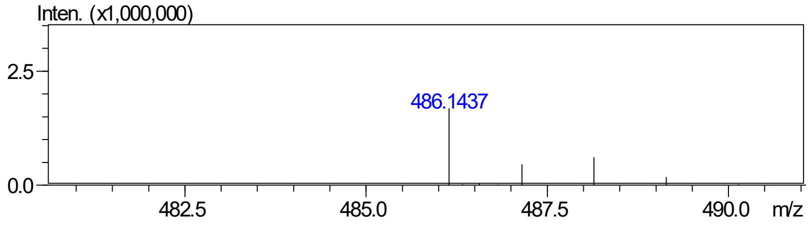

Supplement: Supplementary File 1 [file molecules-23-00709-s001.pdf]
